# Supplementary material for: A novel eukaryotic RdRP-dependent small RNA pathway represses antiviral immunity by controlling an ERK pathway component in the black-legged tick
Source: PLoS One. 2023 Mar 30;18(3):e0281195. doi: 10.1371/journal.pone.0281195 (PMC10062562; doi:10.1371/journal.pone.0281195)
Supplement: S1 Data — On the Normalized and Relative levels pages, normalized read counts (RPM) and relative levels compared to the control (dsGFP) library were used. TEs with more than 50RPM and Coding Genes with more than 3.5RPM on average in KD libraries are included in this website. For viral sequences, reference sequences were generated by assembling sRNA sequences (See the Analysis of sRNAseq data section in Materials and methods.) and size distributions of sRNA reads mapped to each contig are shown. Reads were first grouped by their 5’ nucleotides, and reads of each length were counted. Full tables including TEs and Coding genes with fewer reads are in the “FullTable” folder. (ZIP) [file pone.0281195.s017.zip › SupplementaryData/Links/PDFs/18-30_TE_readcount_libs.pdf]

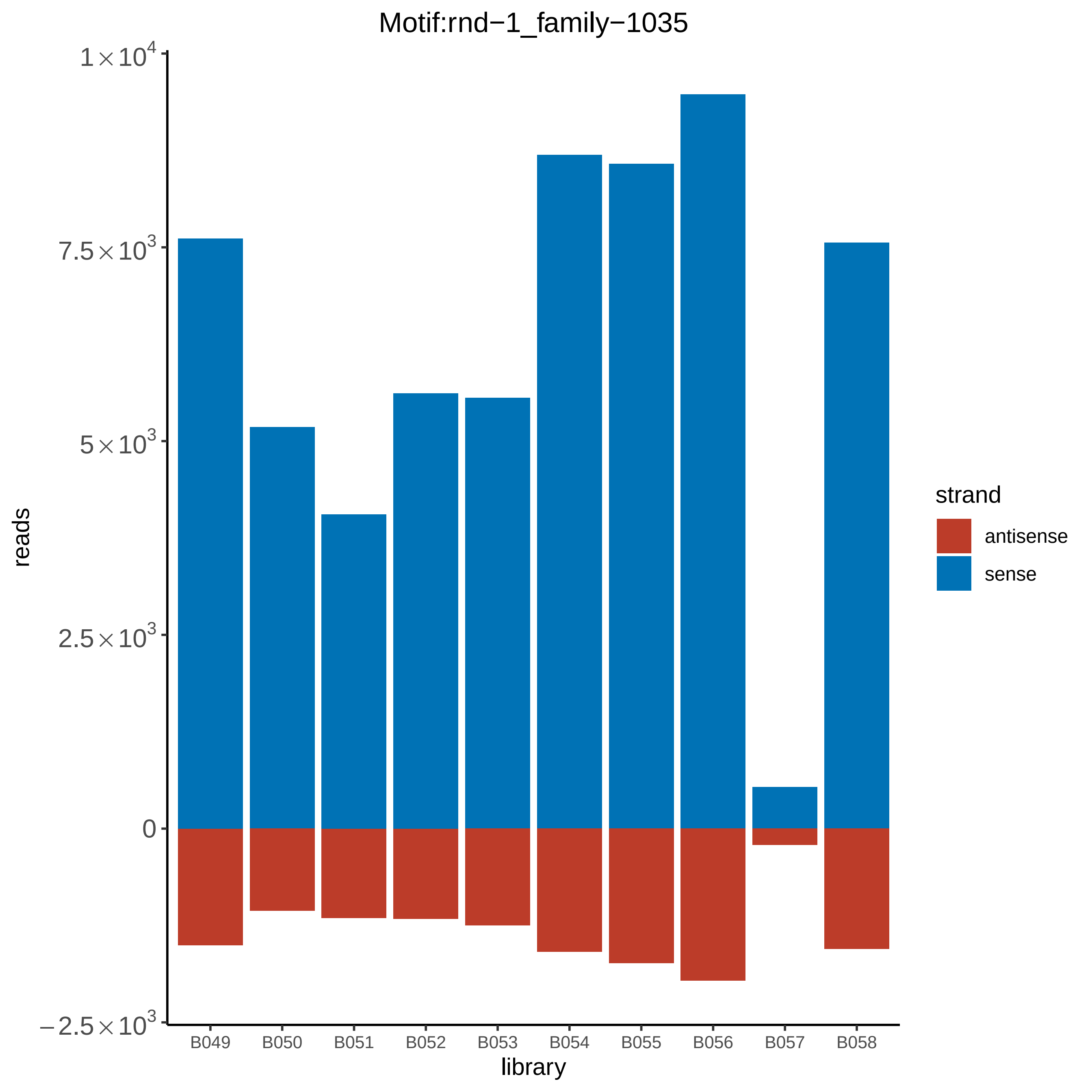

Motif:rnd−1_family−1035
1×10^4
7.5×10^3
5×10^3
2.5×10^3
0
-2.5×10^3
reads
B049
B050
B051
B052
B053
B054
B055
B056
B057
B058
library
strand
antisense
sense

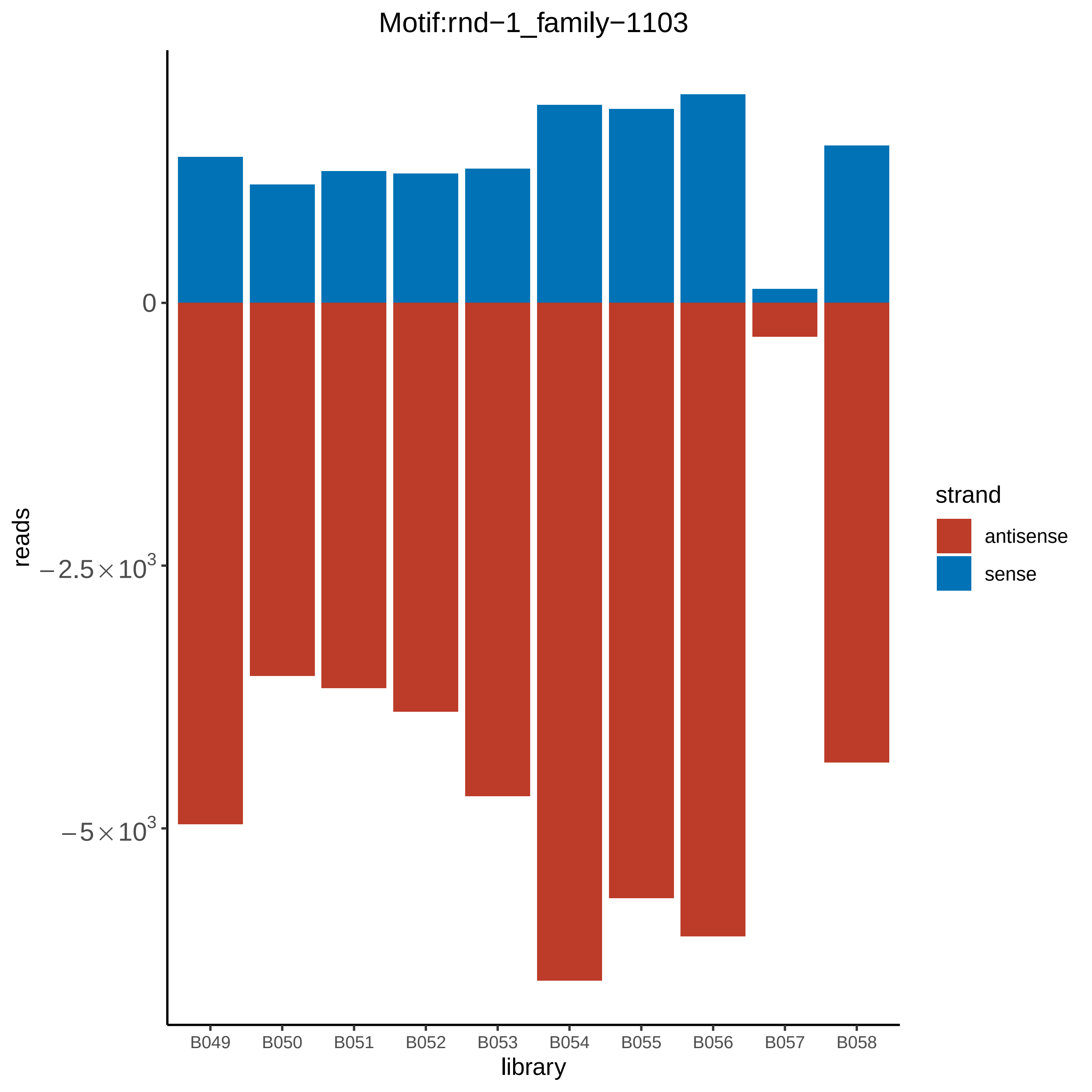
Motif:rnd−1_family−1103
reads
0
−2.5×10^3
−5×10^3
library
B049
B050
B051
B052
B053
B054
B055
B056
B057
B058
strand
antisense
sense

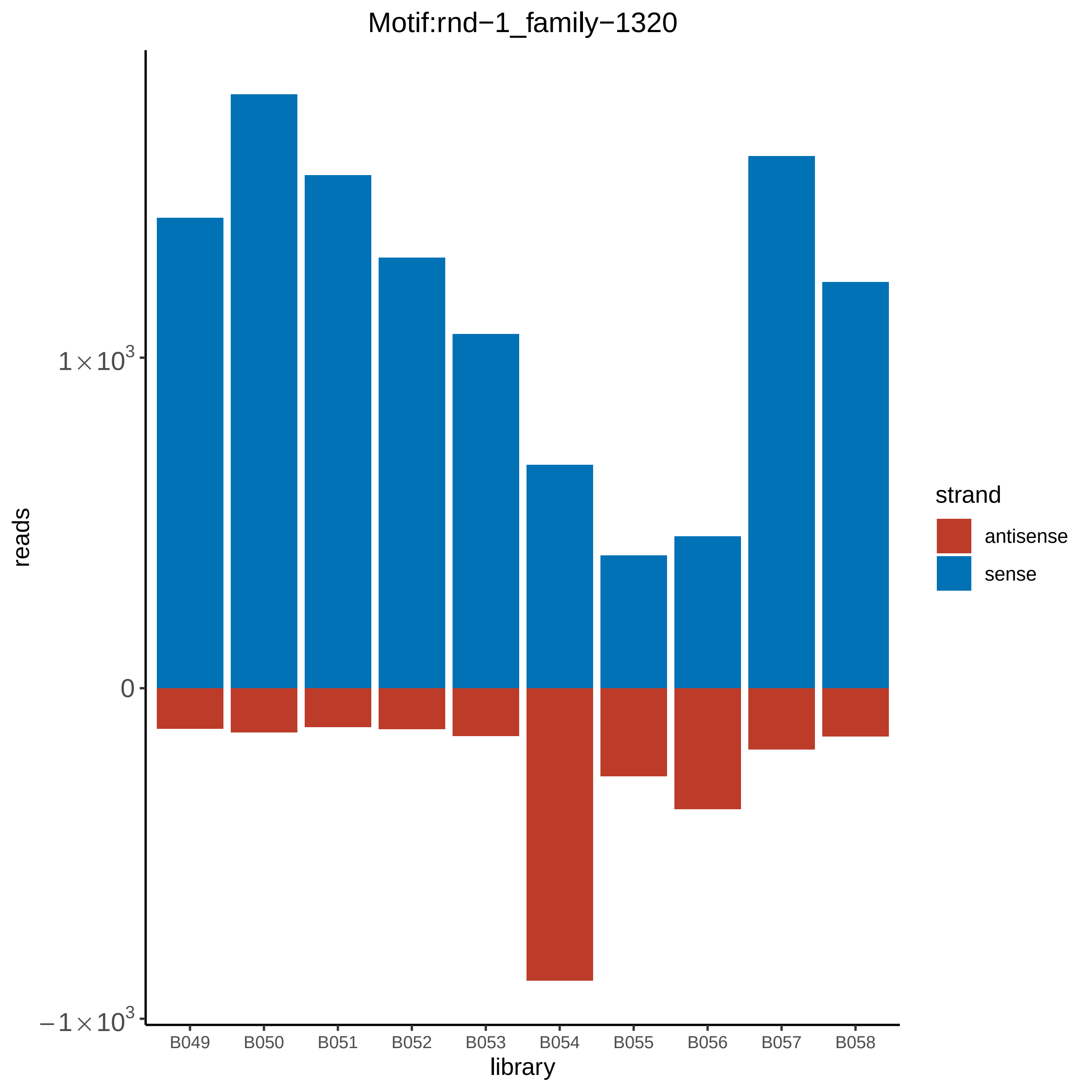

Motif:rnd−1_family−1320
reads
1×10^3
0
−1×10^3
B049
B050
B051
B052
B053
B054
B055
B056
B057
B058
library
strand
antisense
sense

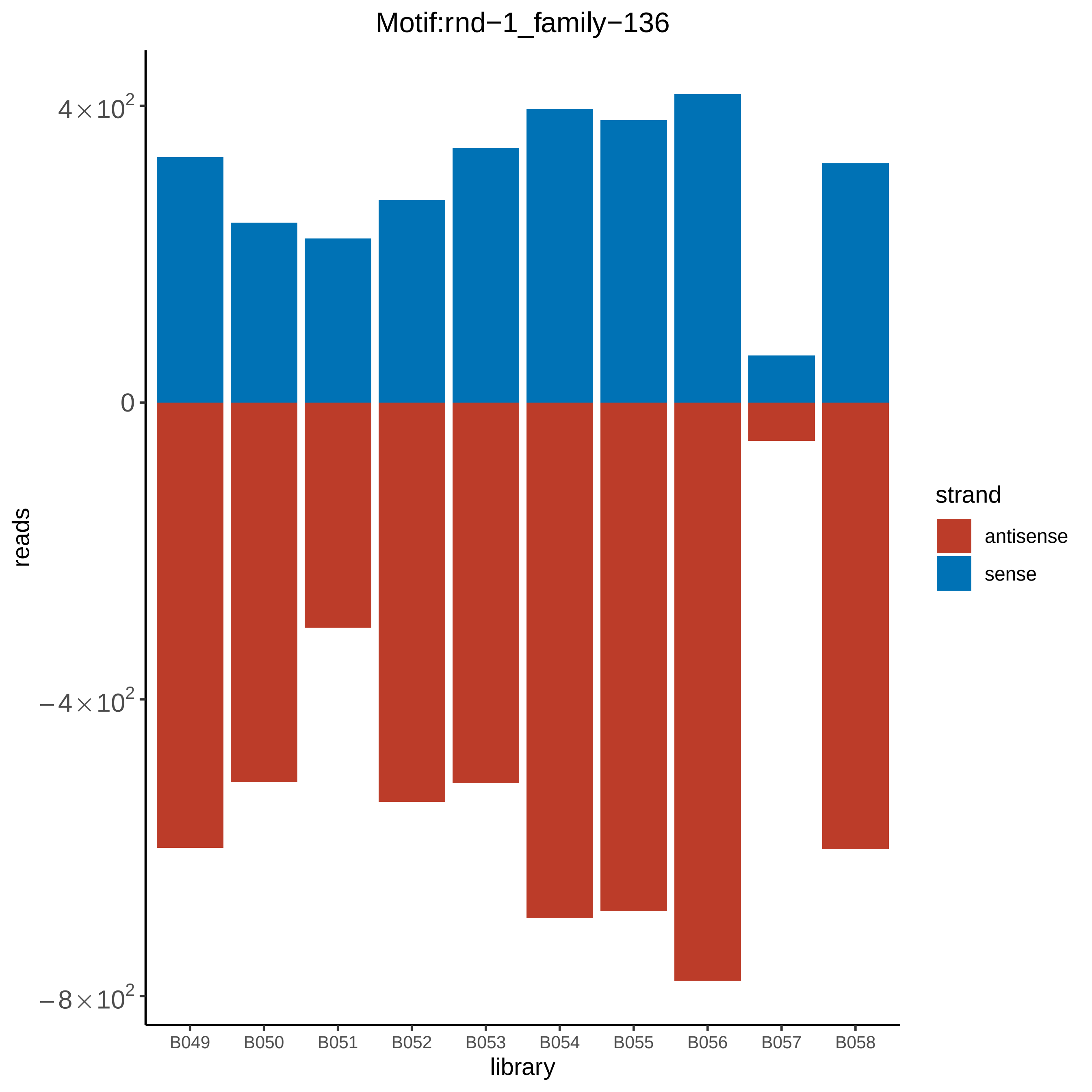
Motif:rnd−1_family−136
reads
4×10^2
0
-4×10^2
-8×10^2
B049
B050
B051
B052
B053
B054
B055
B056
B057
B058
library
strand
antisense
sense

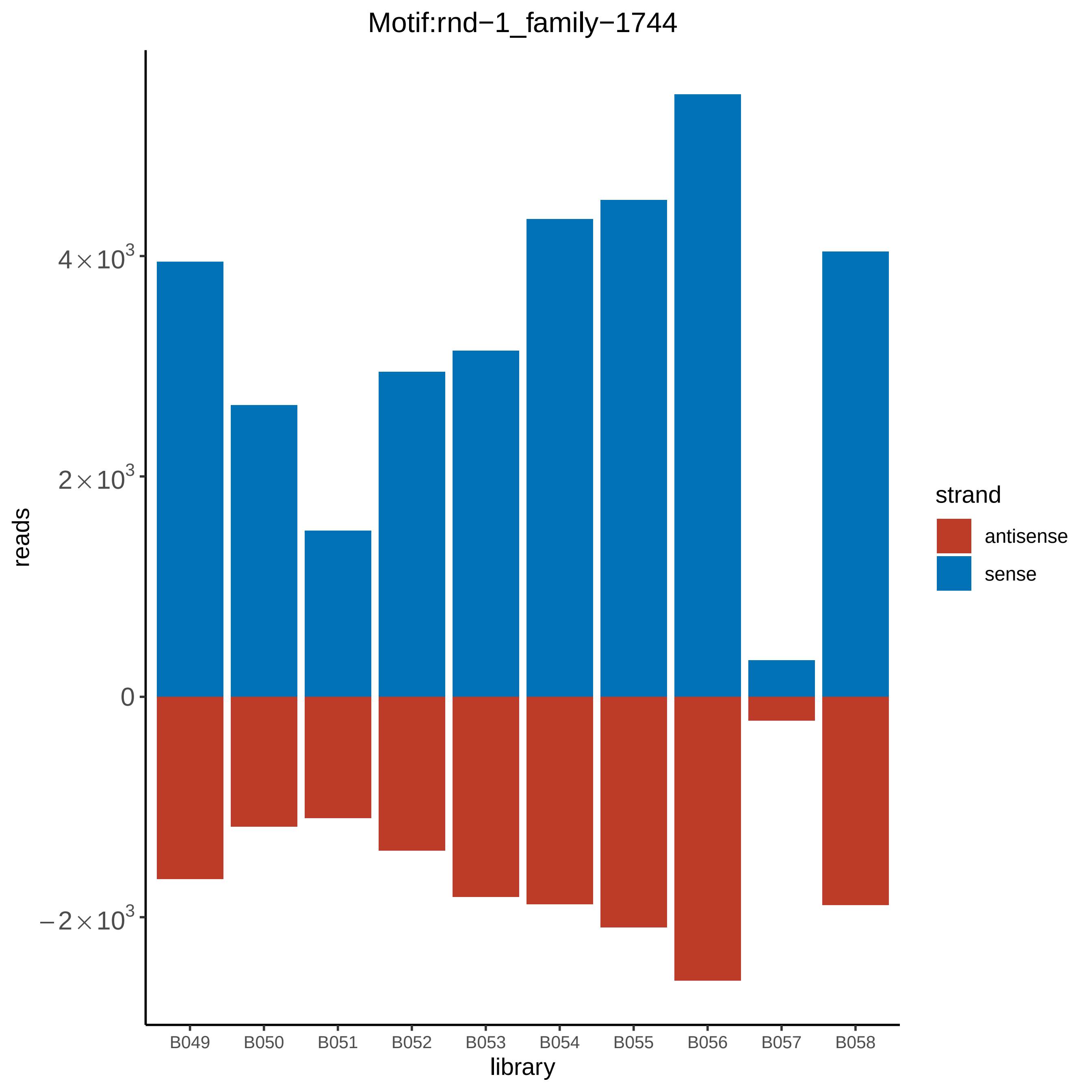
Motif:rnd−1_family−1744
reads
4×10^3
2×10^3
0
-2×10^3
library
B049
B050
B051
B052
B053
B054
B055
B056
B057
B058
strand
antisense
sense

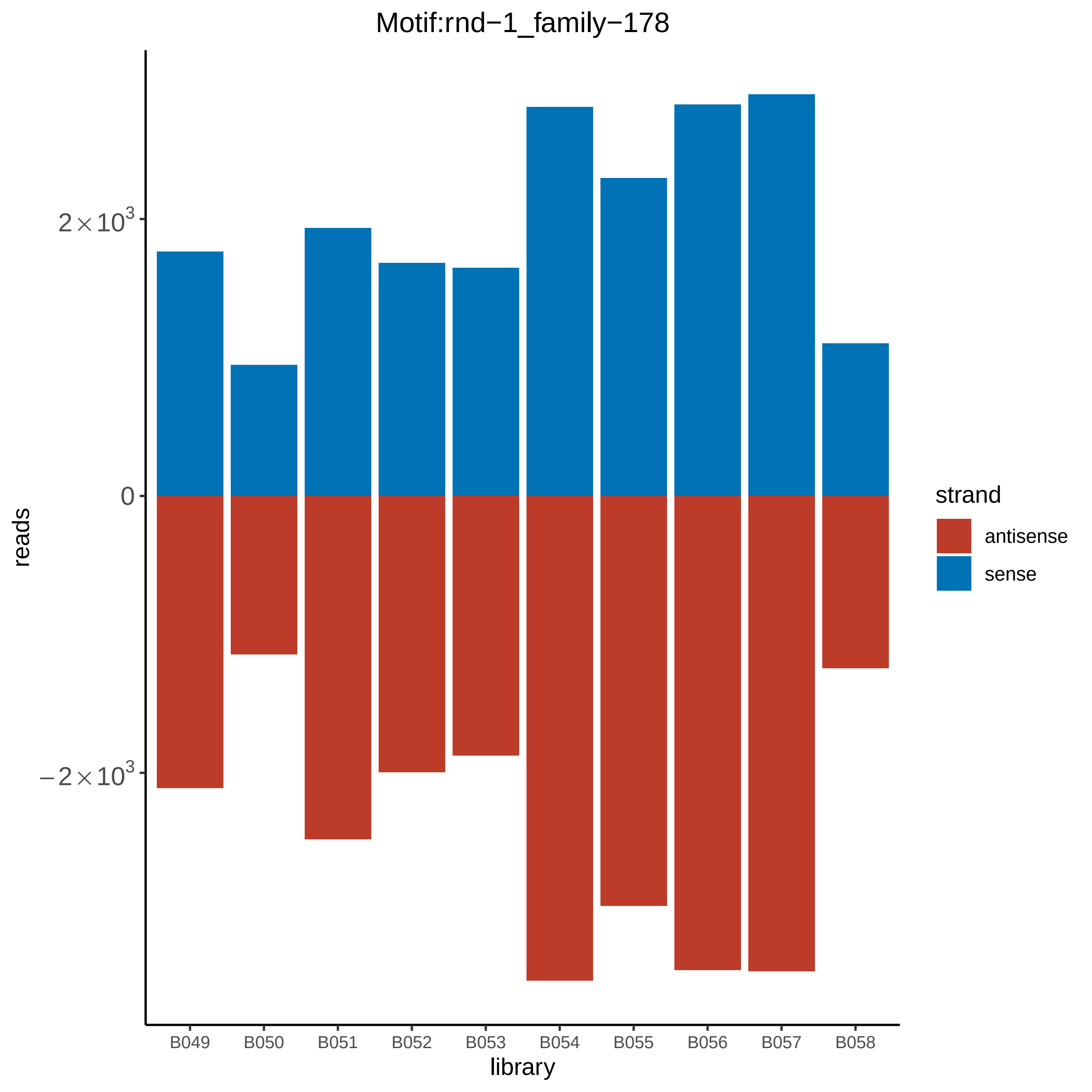

Motif:rnd−1_family−178
2×10^3
0
-2×10^3
reads
B049
B050
B051
B052
B053
B054
B055
B056
B057
B058
library
strand
antisense
sense

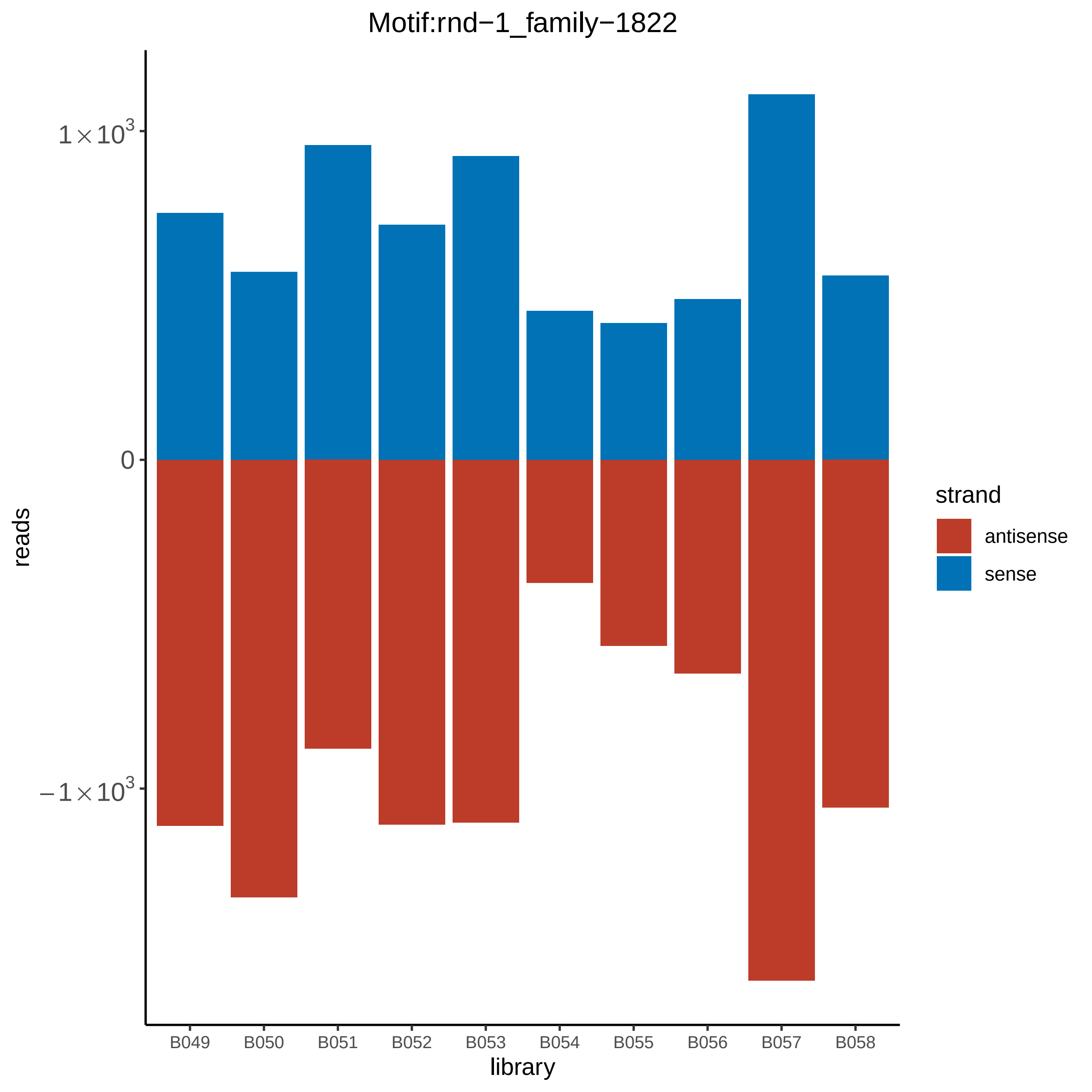

Motif:rnd−1_family−1822
1×10^3
0
-1×10^3
reads
B049
B050
B051
B052
B053
B054
B055
B056
B057
B058
library
strand
antisense
sense

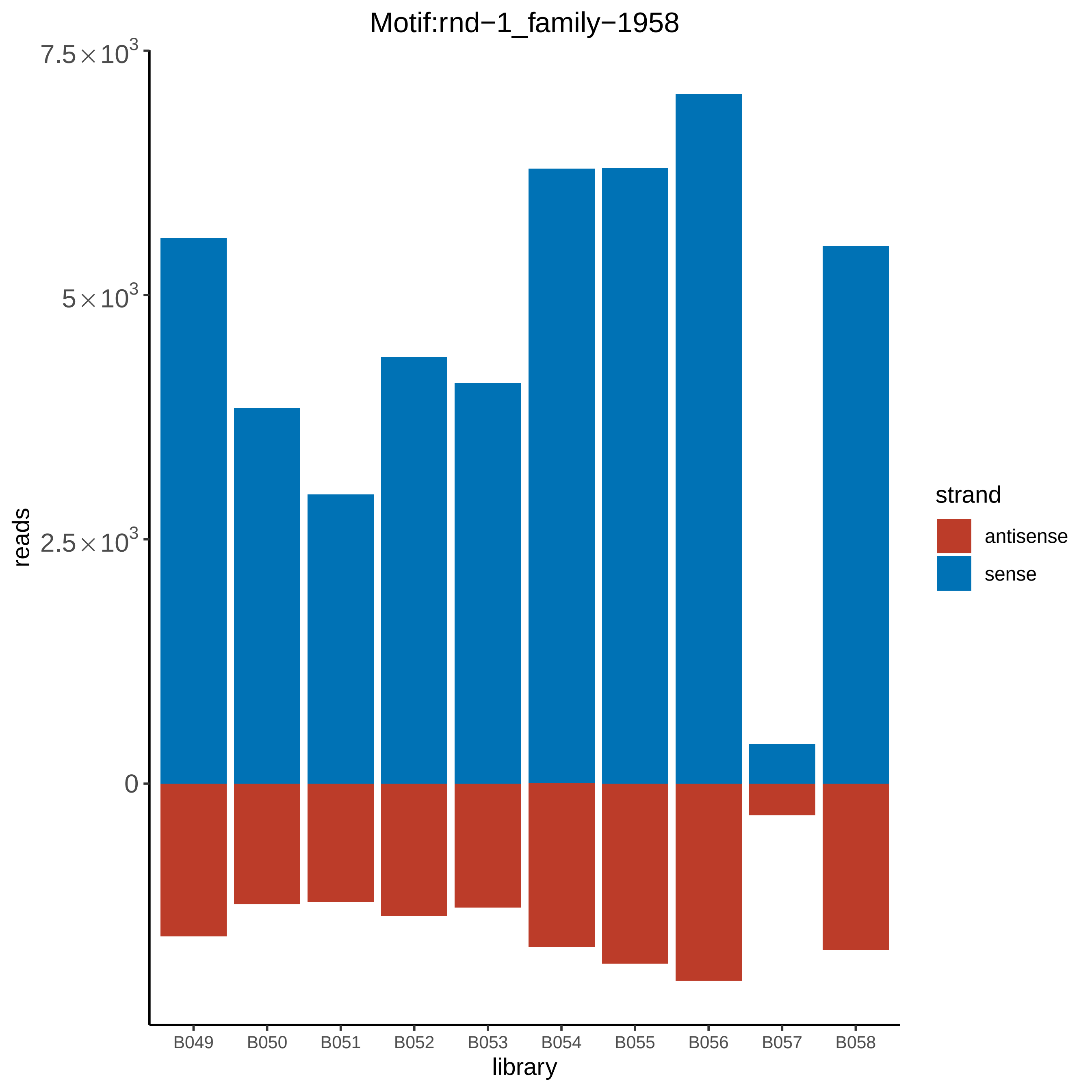

Motif:rnd−1_family−1958
7.5×10^3
5×10^3
2.5×10^3
0
reads
B049
B050
B051
B052
B053
B054
B055
B056
B057
B058
library
strand
antisense
sense

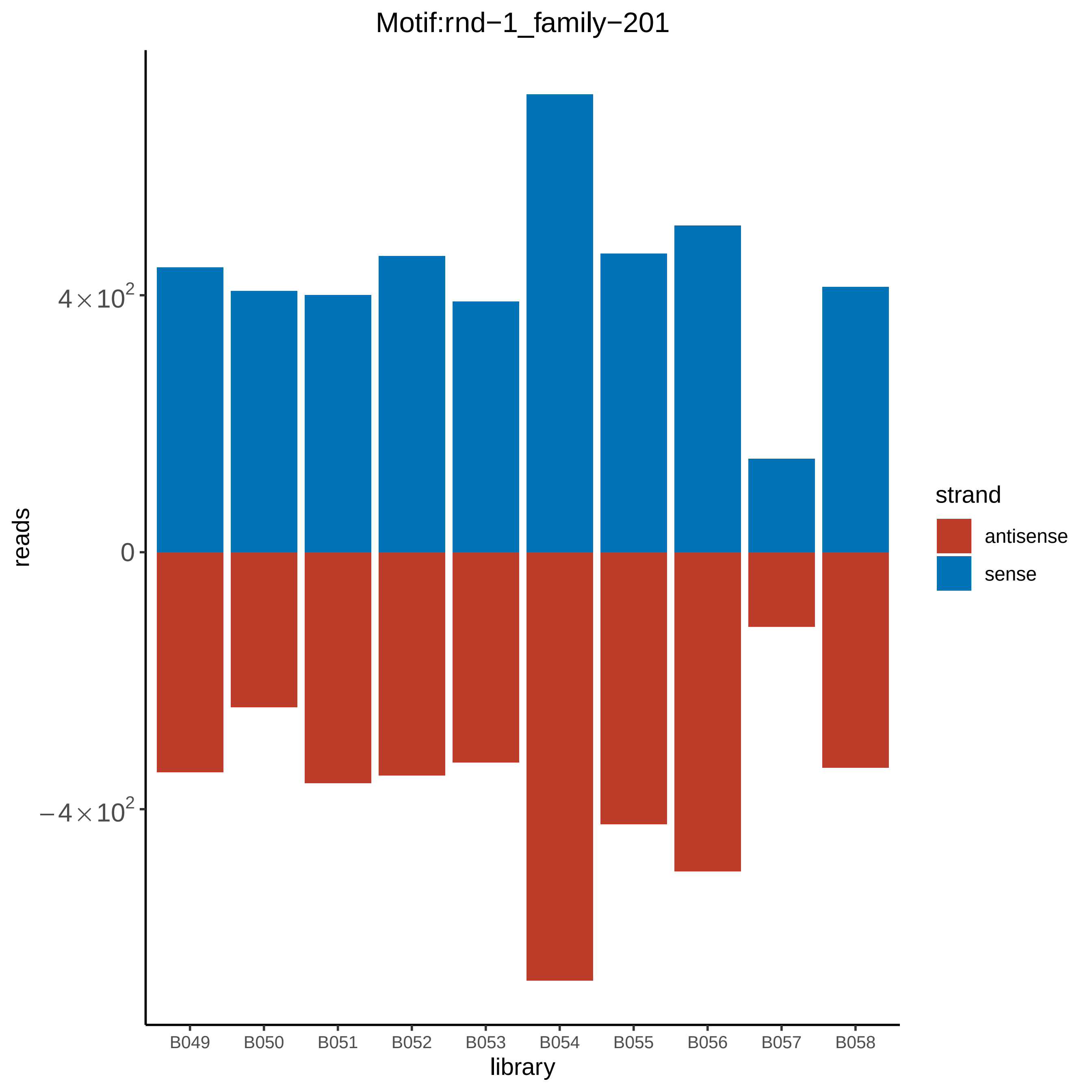

Motif:rnd−1_family−201
reads
4×10^2
0
−4×10^2
library
B049
B050
B051
B052
B053
B054
B055
B056
B057
B058
strand
antisense
sense

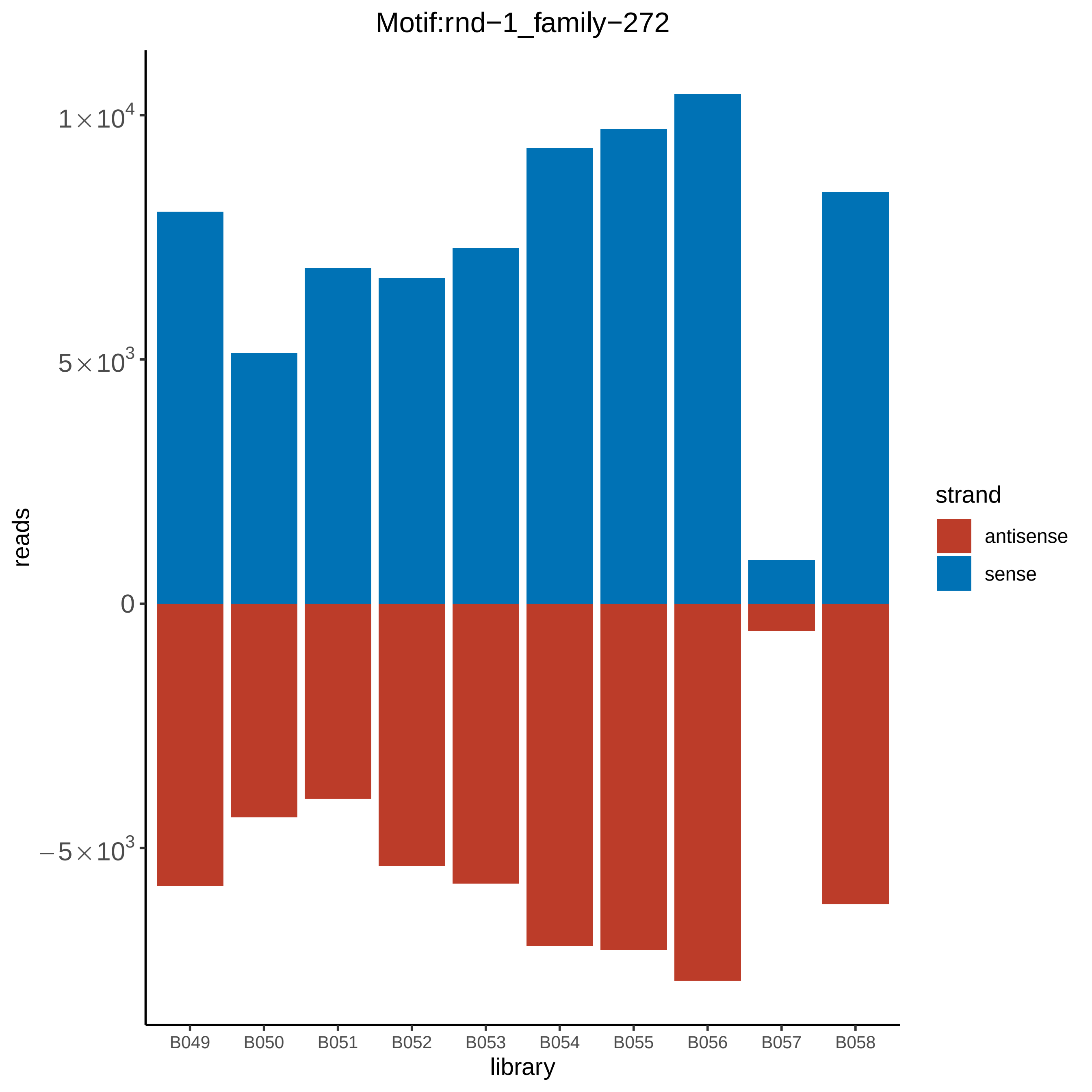
Motif:rnd−1_family−272
reads
library
strand
antisense
sense
1×10^4
5×10^3
0
−5×10^3
B049
B050
B051
B052
B053
B054
B055
B056
B057
B058

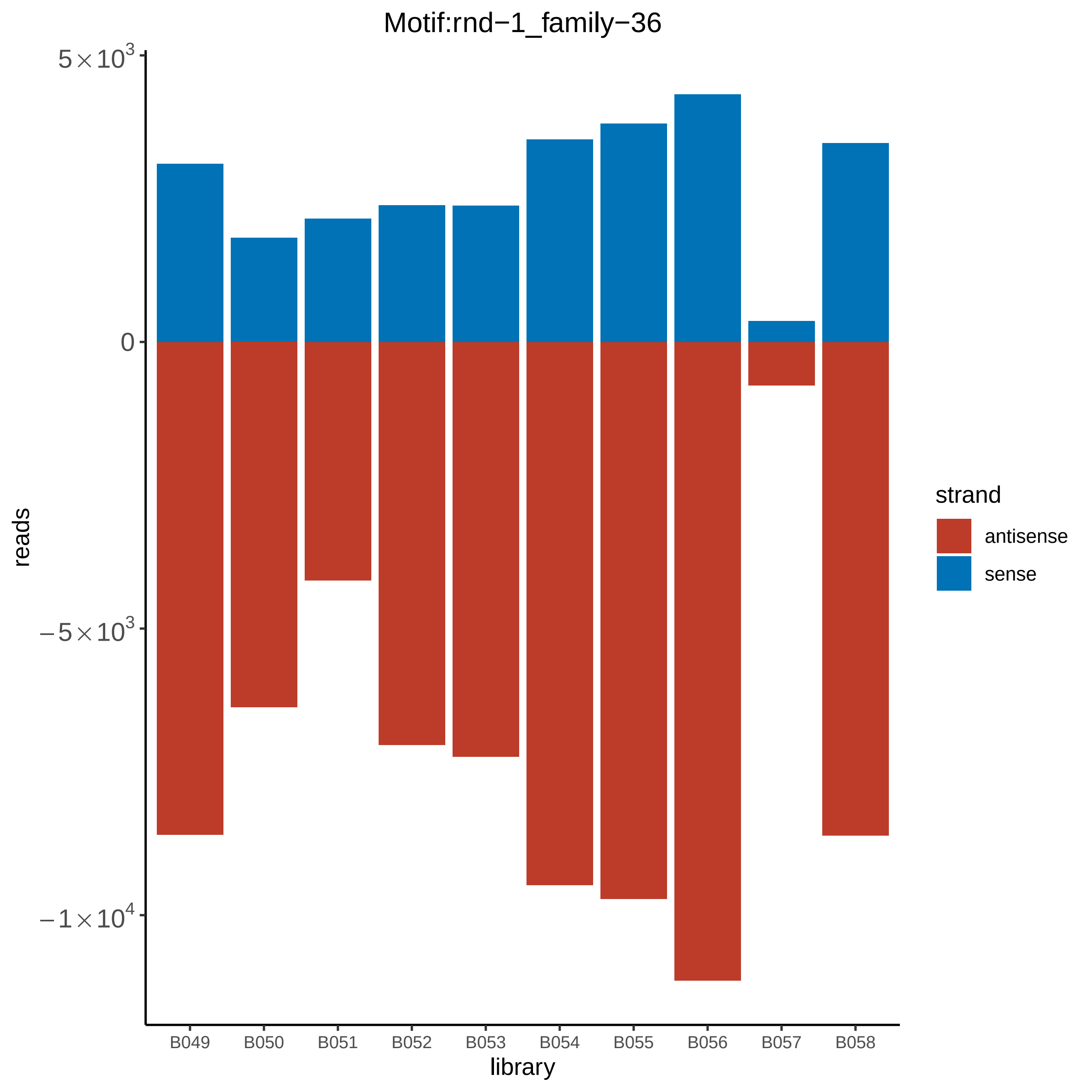

Motif:rnd−1_family−36
5×10^3
0
-5×10^3
-1×10^4
reads
B049
B050
B051
B052
B053
B054
B055
B056
B057
B058
library
strand
antisense
sense

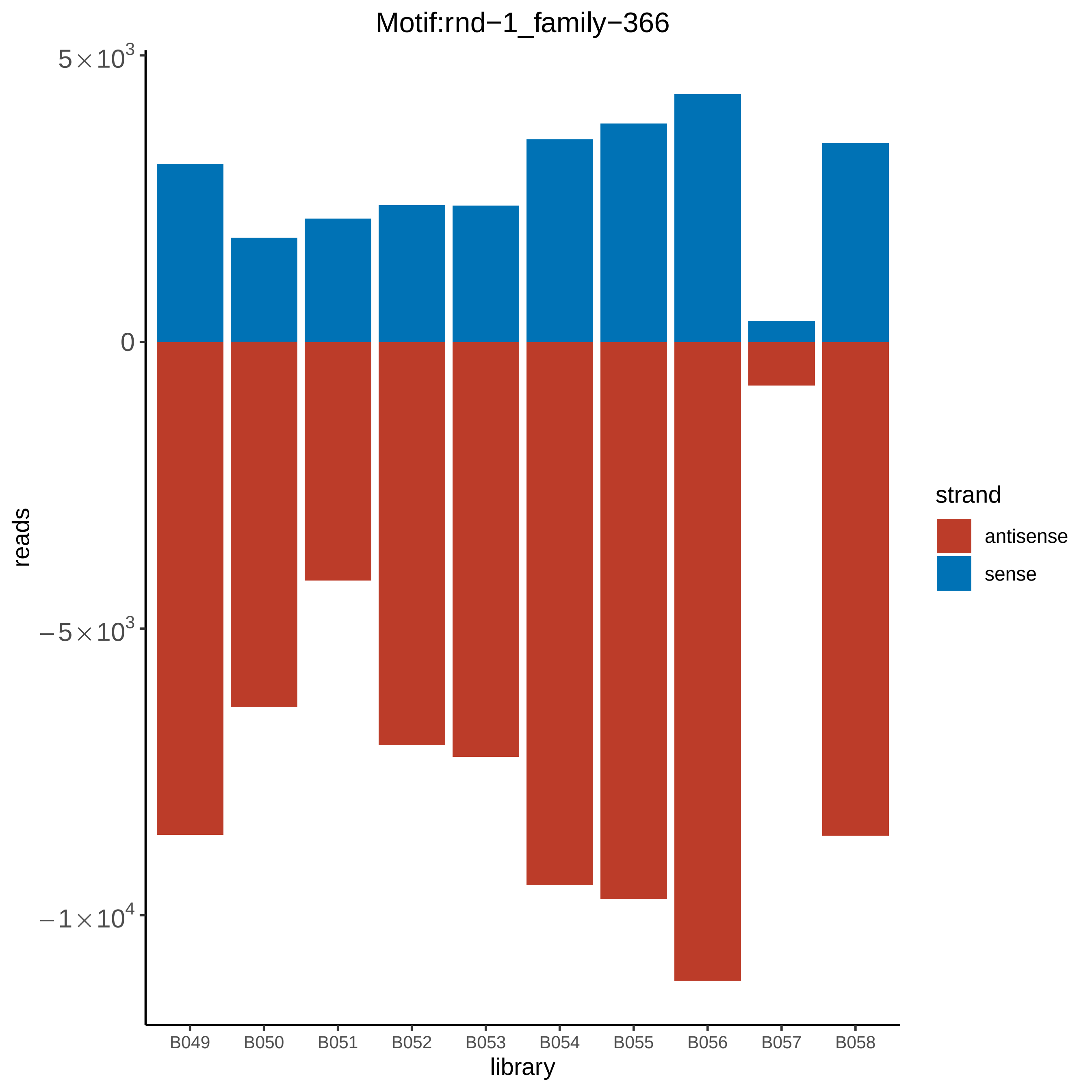

Motif:rnd−1_family−366
5×10^3
0
-5×10^3
-1×10^4
reads
B049
B050
B051
B052
B053
B054
B055
B056
B057
B058
library
strand
antisense
sense

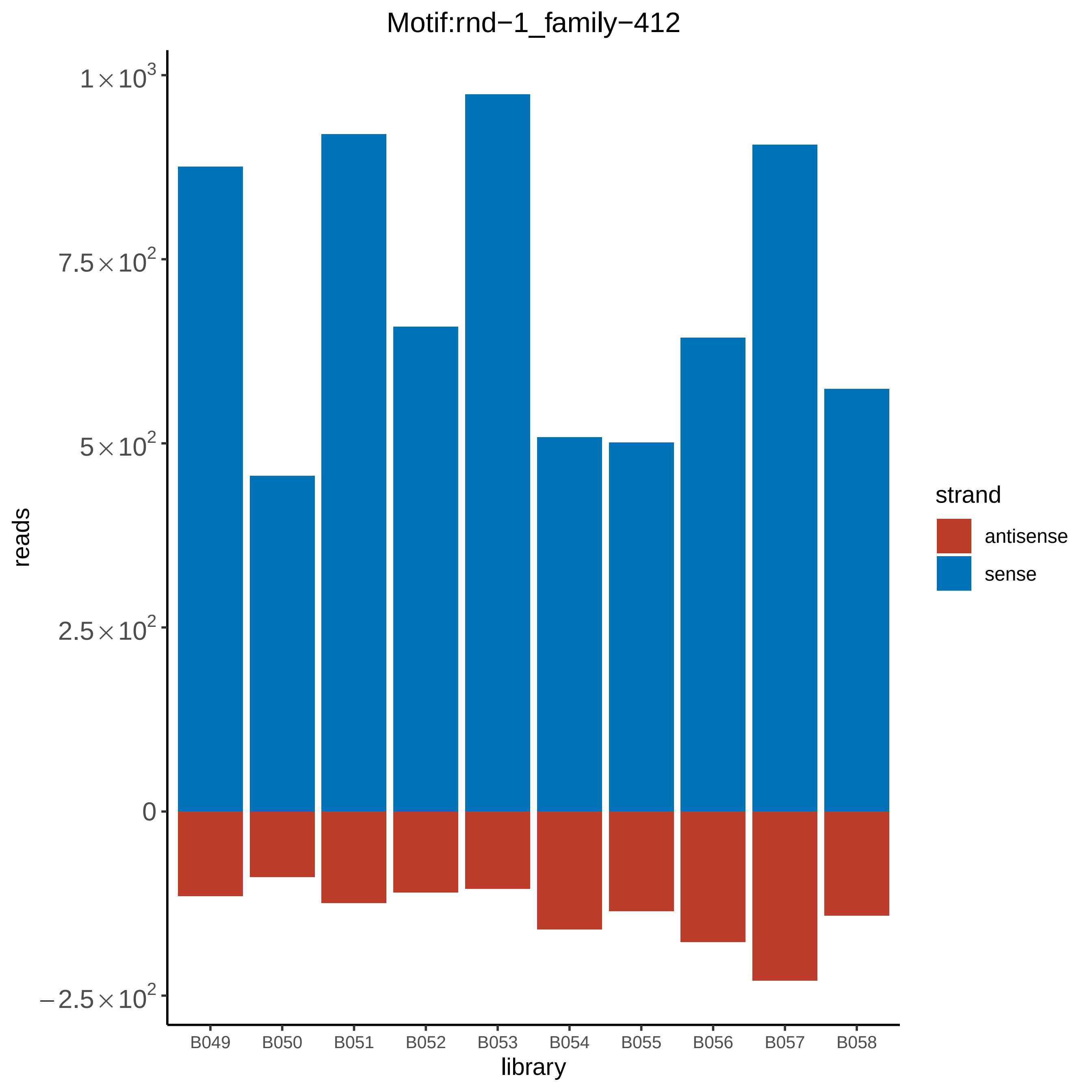

Motif:rnd−1_family−412
reads
library
1×10^3
7.5×10^2
5×10^2
2.5×10^2
0
−2.5×10^2
B049
B050
B051
B052
B053
B054
B055
B056
B057
B058
strand
antisense
sense

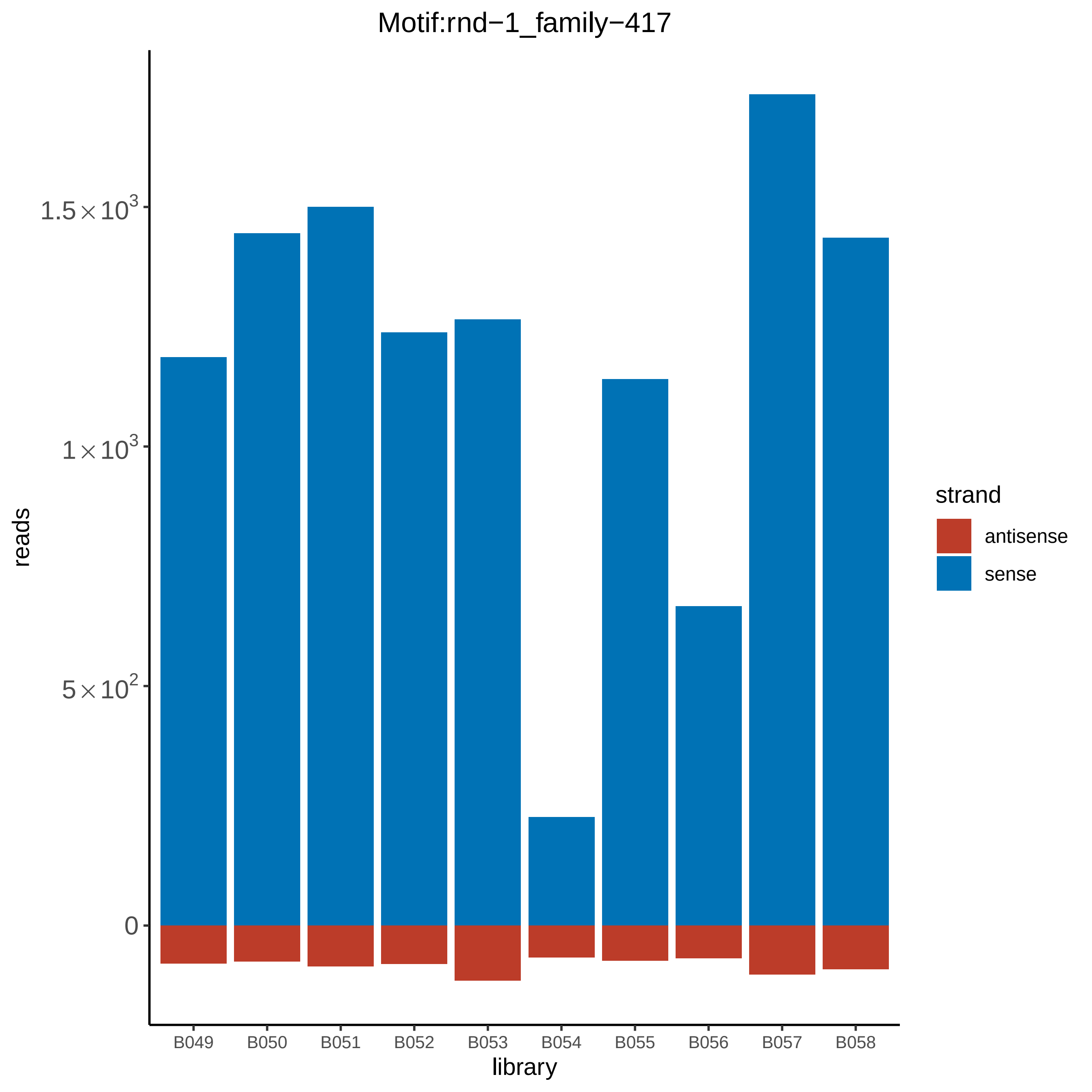
Motif:rnd−1_family−417
reads
1.5×10^3
1×10^3
5×10^2
0
B049
B050
B051
B052
B053
B054
B055
B056
B057
B058
library
strand
antisense
sense

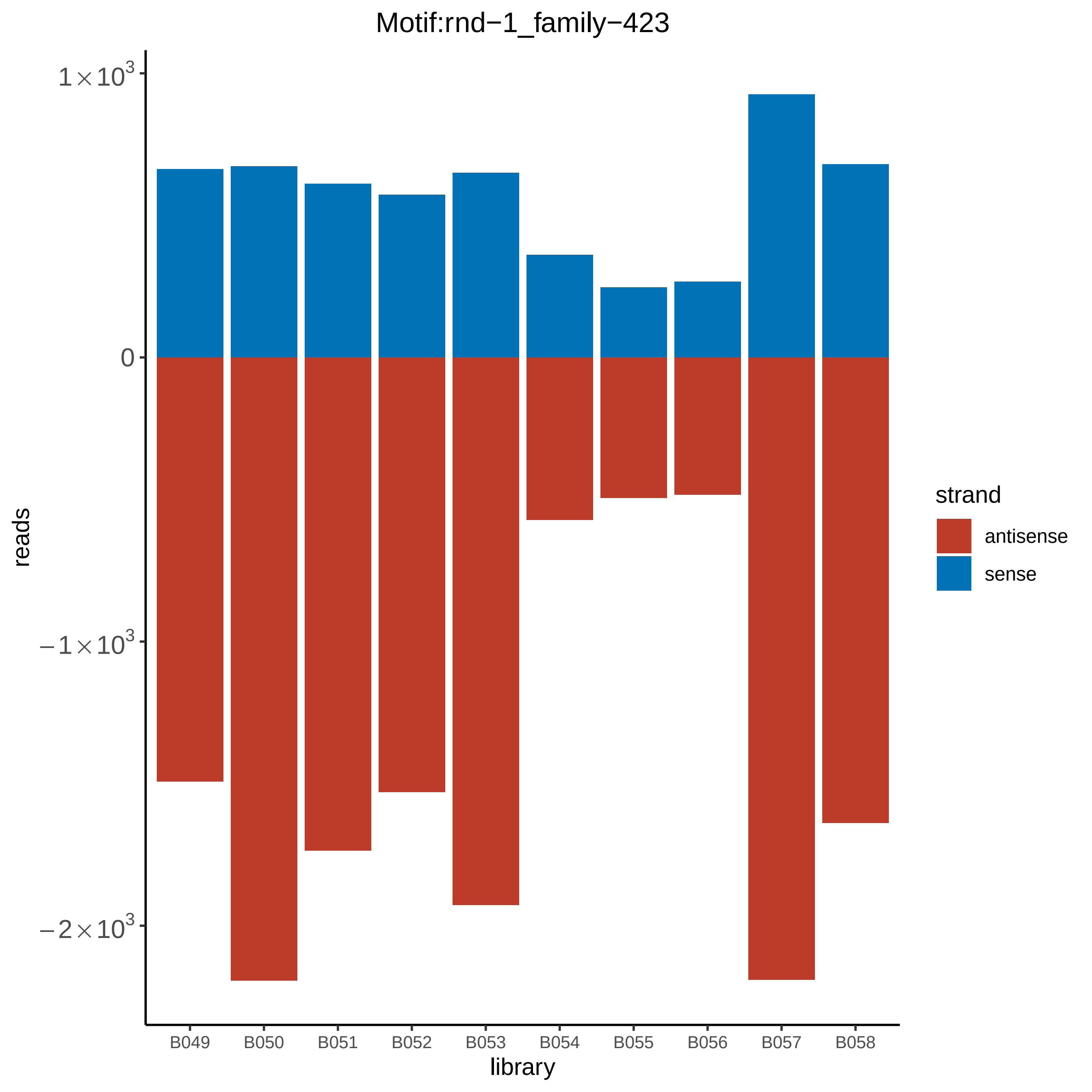

Motif:rnd−1_family−423
reads
1×10^3
0
−1×10^3
−2×10^3
B049
B050
B051
B052
B053
B054
B055
B056
B057
B058
library
strand
antisense
sense

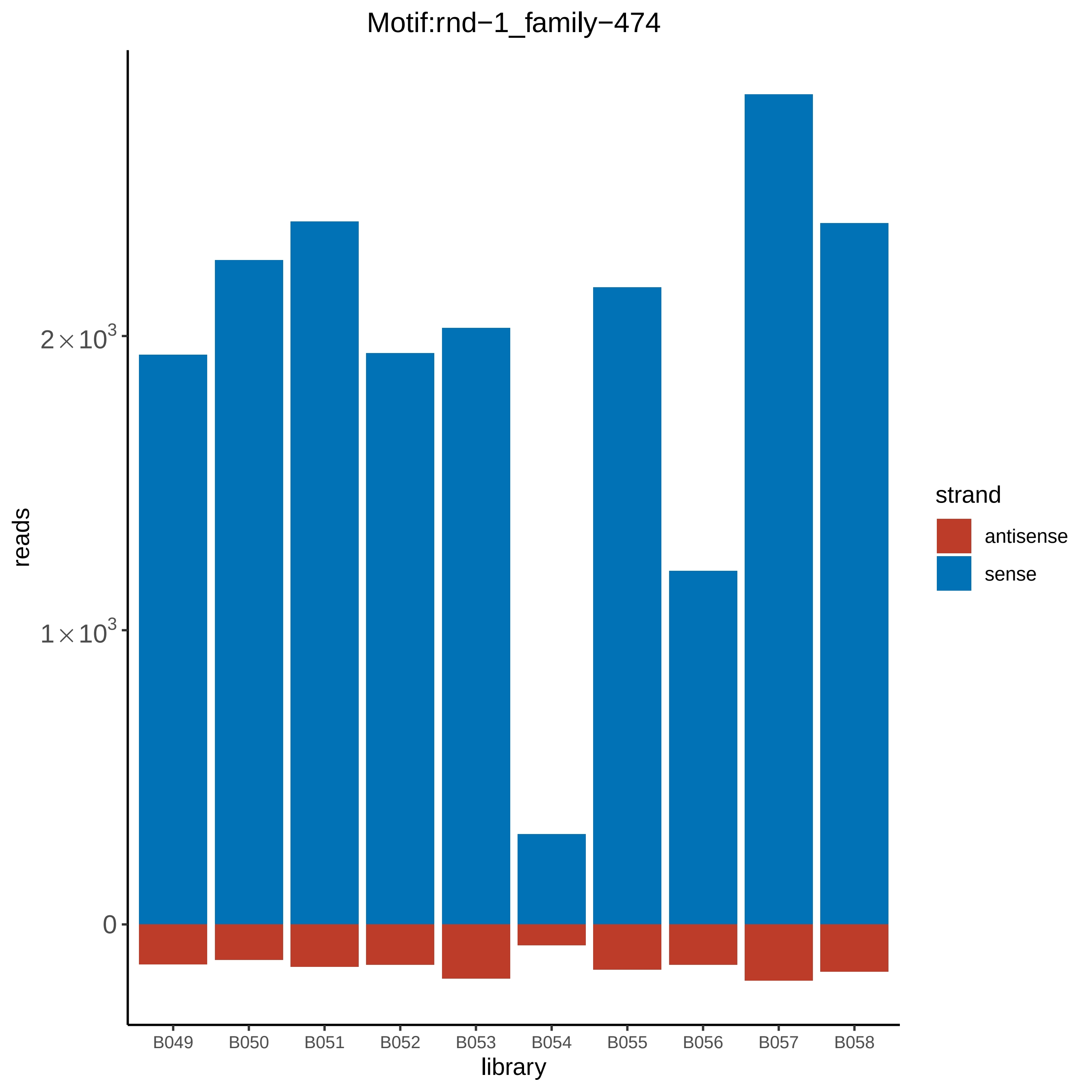

Motif:rnd−1_family−474
reads
2×10^3
1×10^3
0
library
B049
B050
B051
B052
B053
B054
B055
B056
B057
B058
strand
antisense
sense

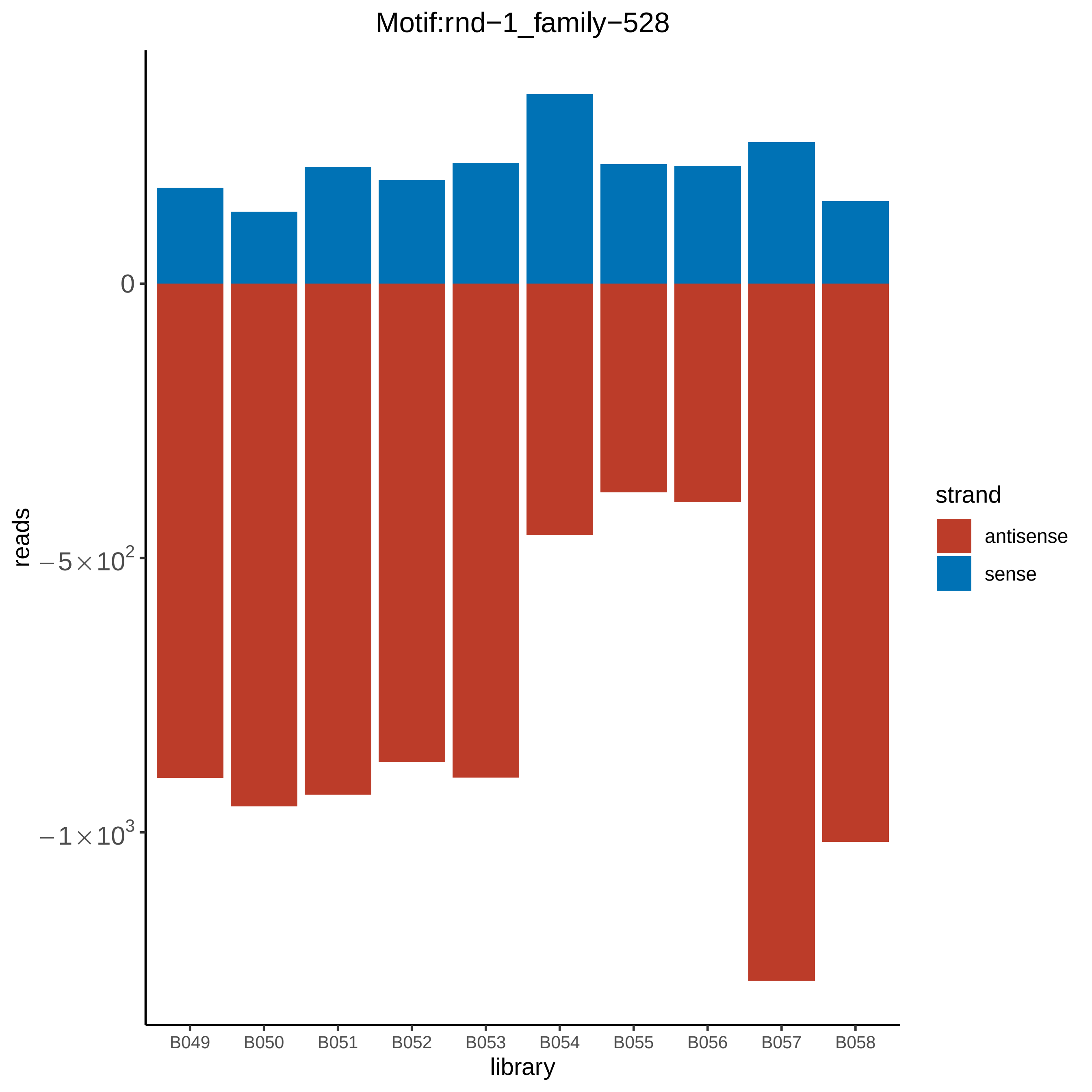

Motif:rnd−1_family−528
reads
0
−5×10^2
−1×10^3
library
B049
B050
B051
B052
B053
B054
B055
B056
B057
B058
strand
antisense
sense

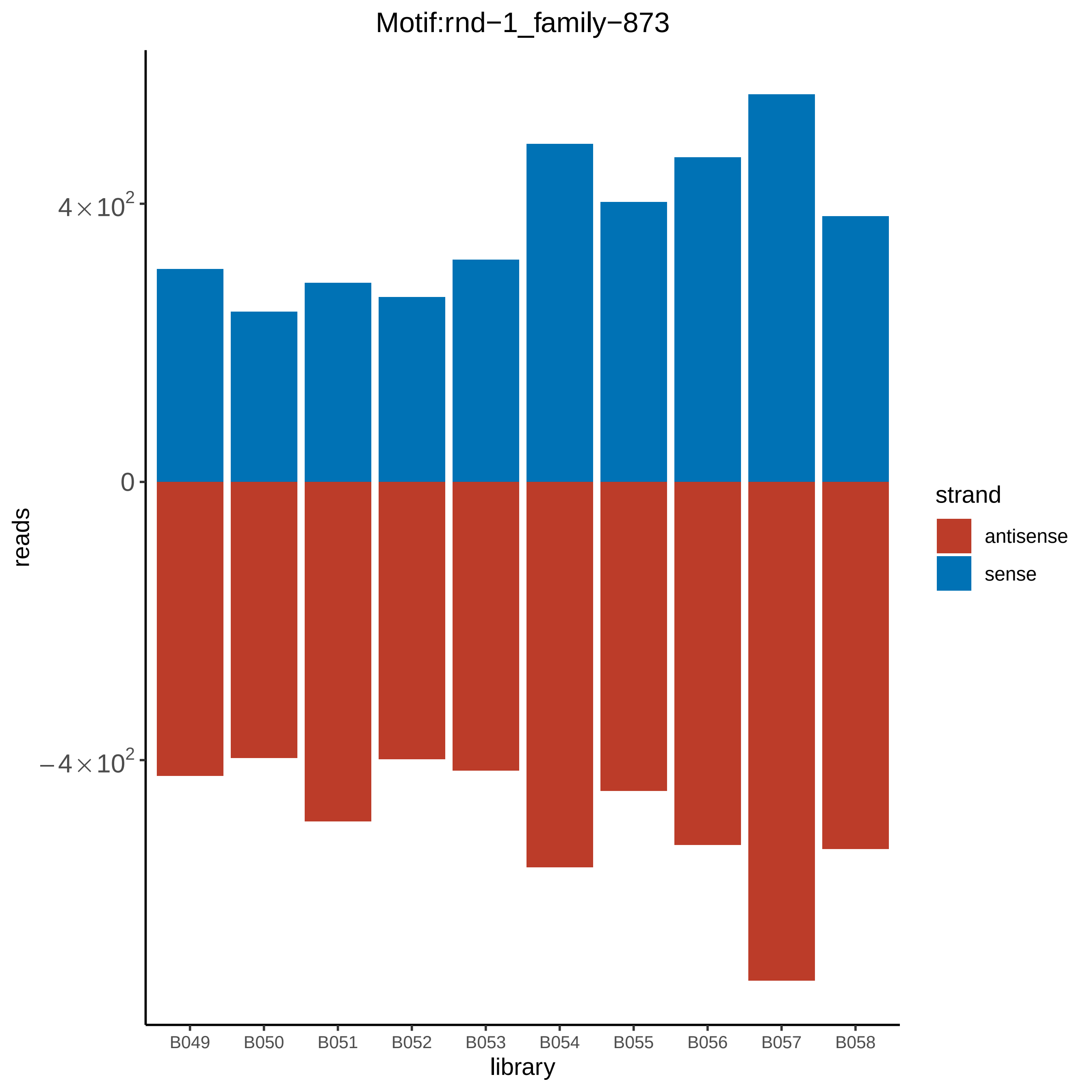

Motif:rnd−1_family−873
reads
4×10^2
0
-4×10^2
B049
B050
B051
B052
B053
B054
B055
B056
B057
B058
library
strand
antisense
sense

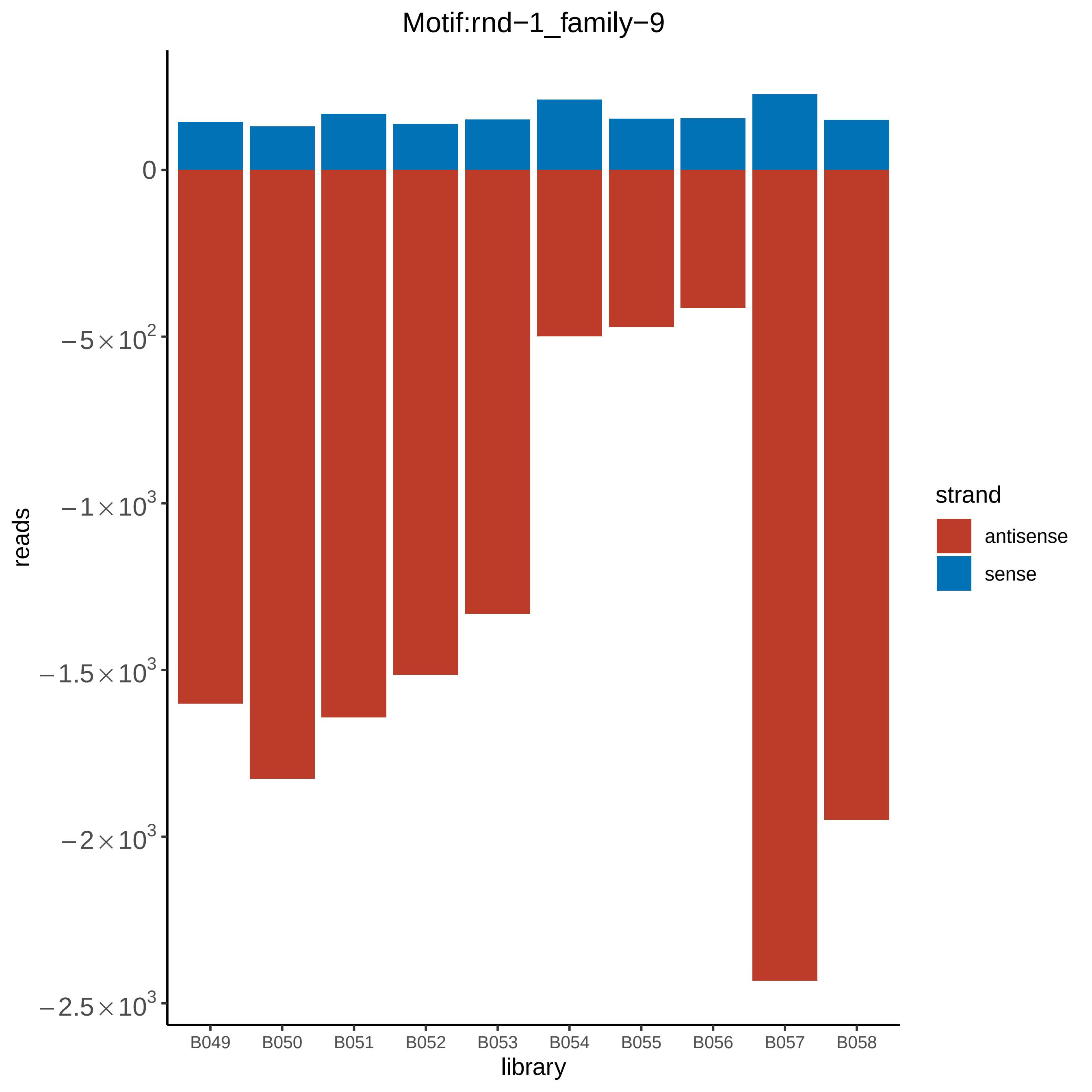

Motif:rnd−1_family−9
0
−5×10^2
−1×10^3
−1.5×10^3
−2×10^3
−2.5×10^3
reads
B049
B050
B051
B052
B053
B054
B055
B056
B057
B058
library
strand
antisense
sense

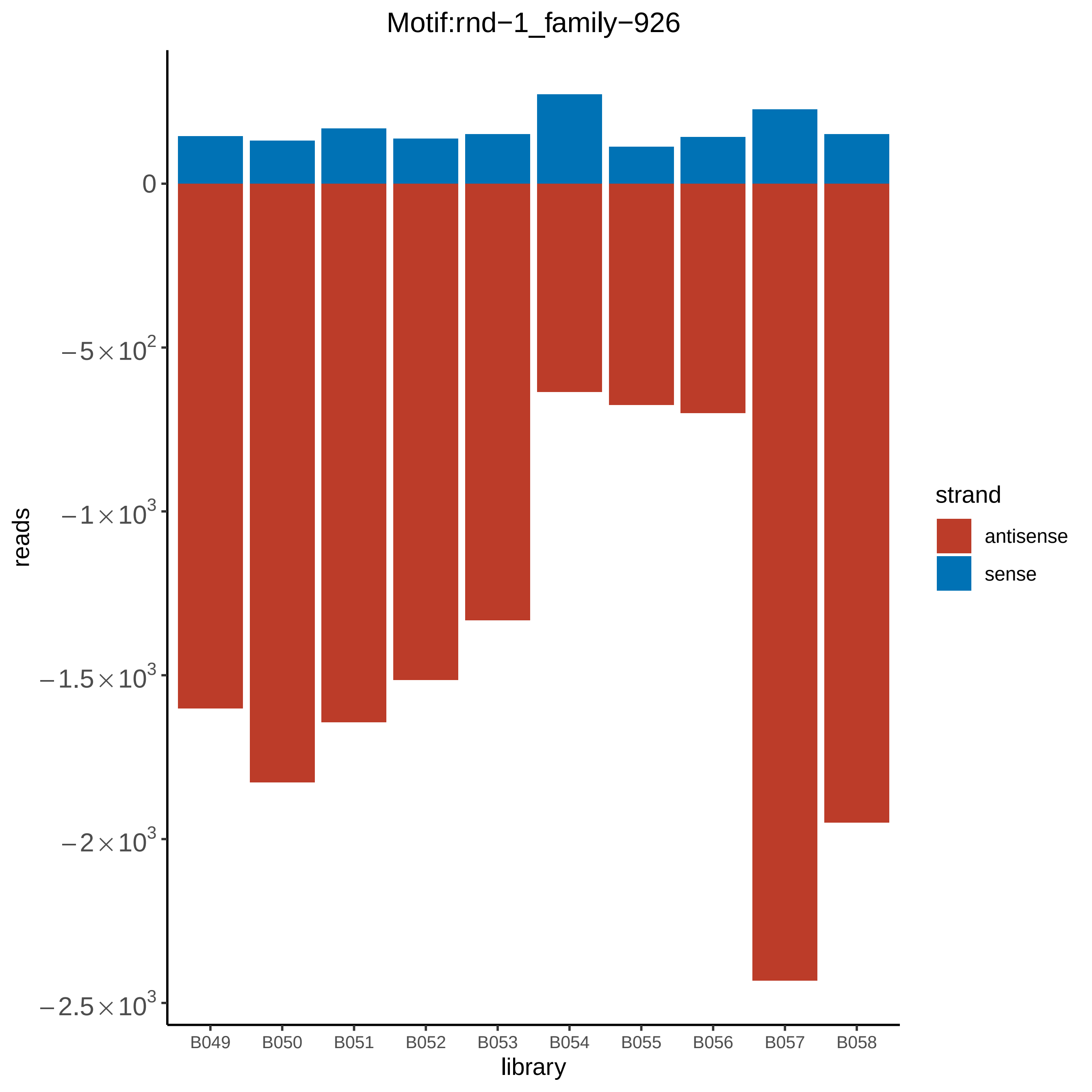
Motif:rnd−1_family−926
0
−5×10^2
−1×10^3
−1.5×10^3
−2×10^3
−2.5×10^3
reads
B049
B050
B051
B052
B053
B054
B055
B056
B057
B058
library
strand
antisense
sense

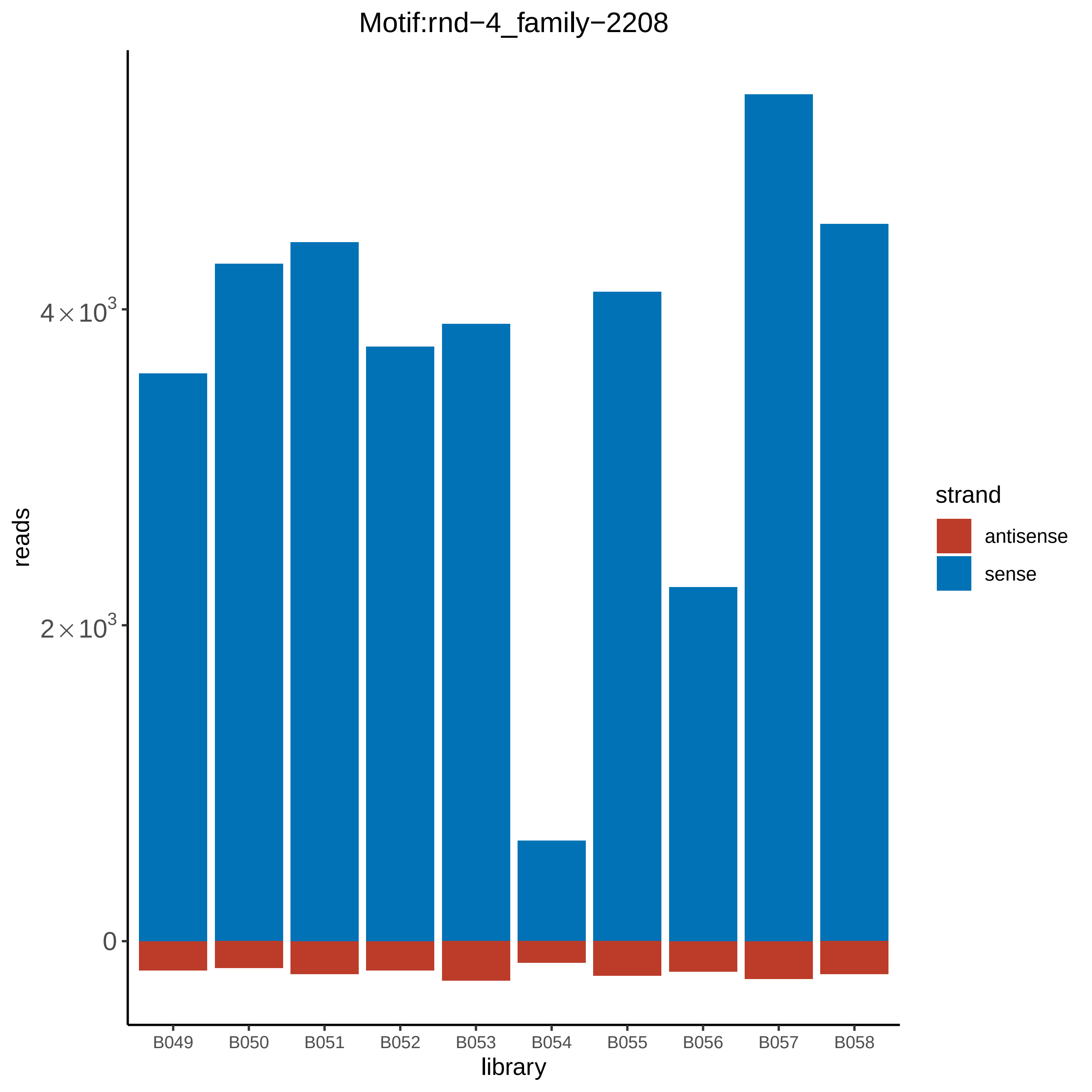

Motif:rnd−4_family−2208
reads
4×10^3
2×10^3
0
library
B049
B050
B051
B052
B053
B054
B055
B056
B057
B058
strand
antisense
sense

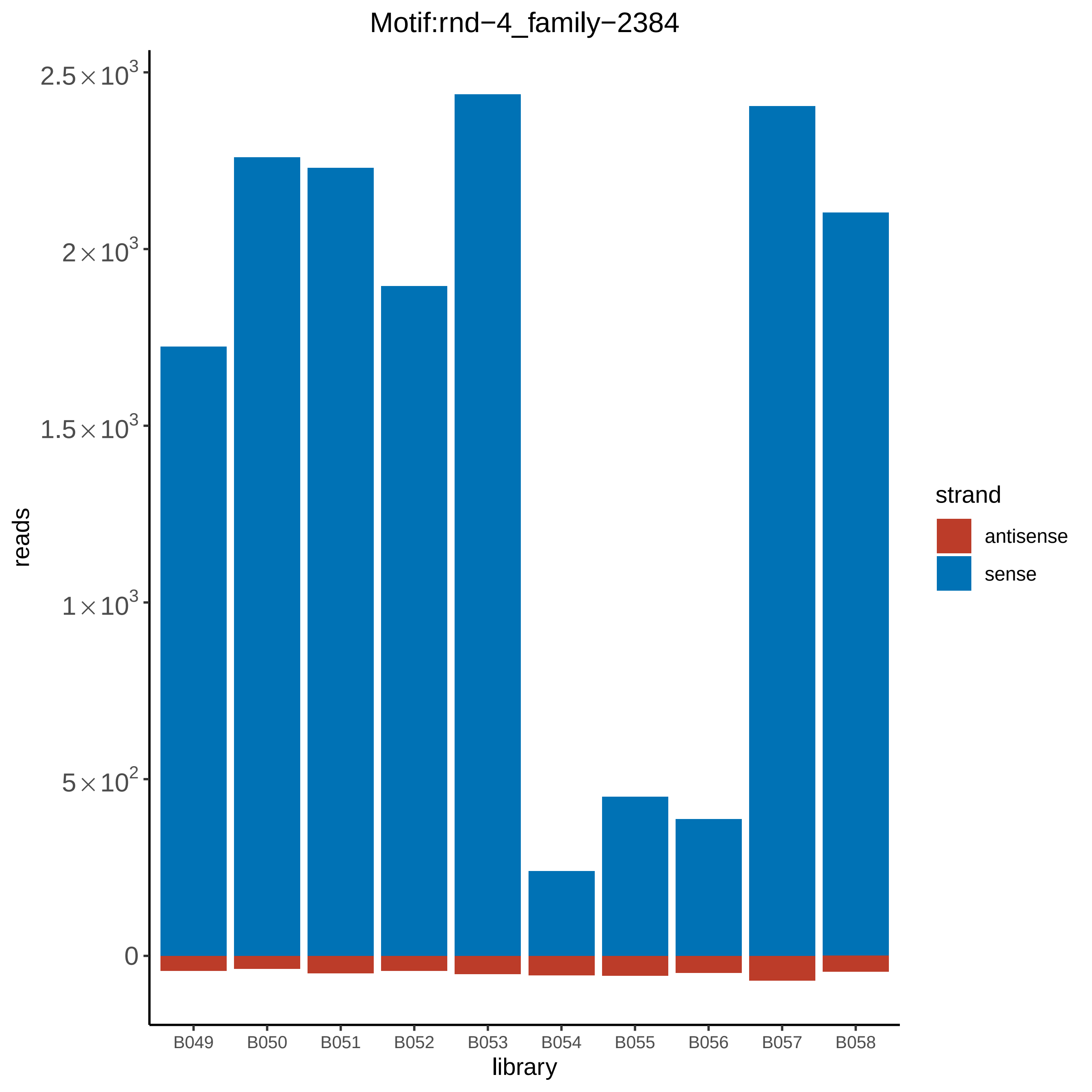

Motif:rnd−4_family−2384
2.5×10^3
2×10^3
1.5×10^3
1×10^3
5×10^2
0
reads
B049
B050
B051
B052
B053
B054
B055
B056
B057
B058
library
strand
antisense
sense

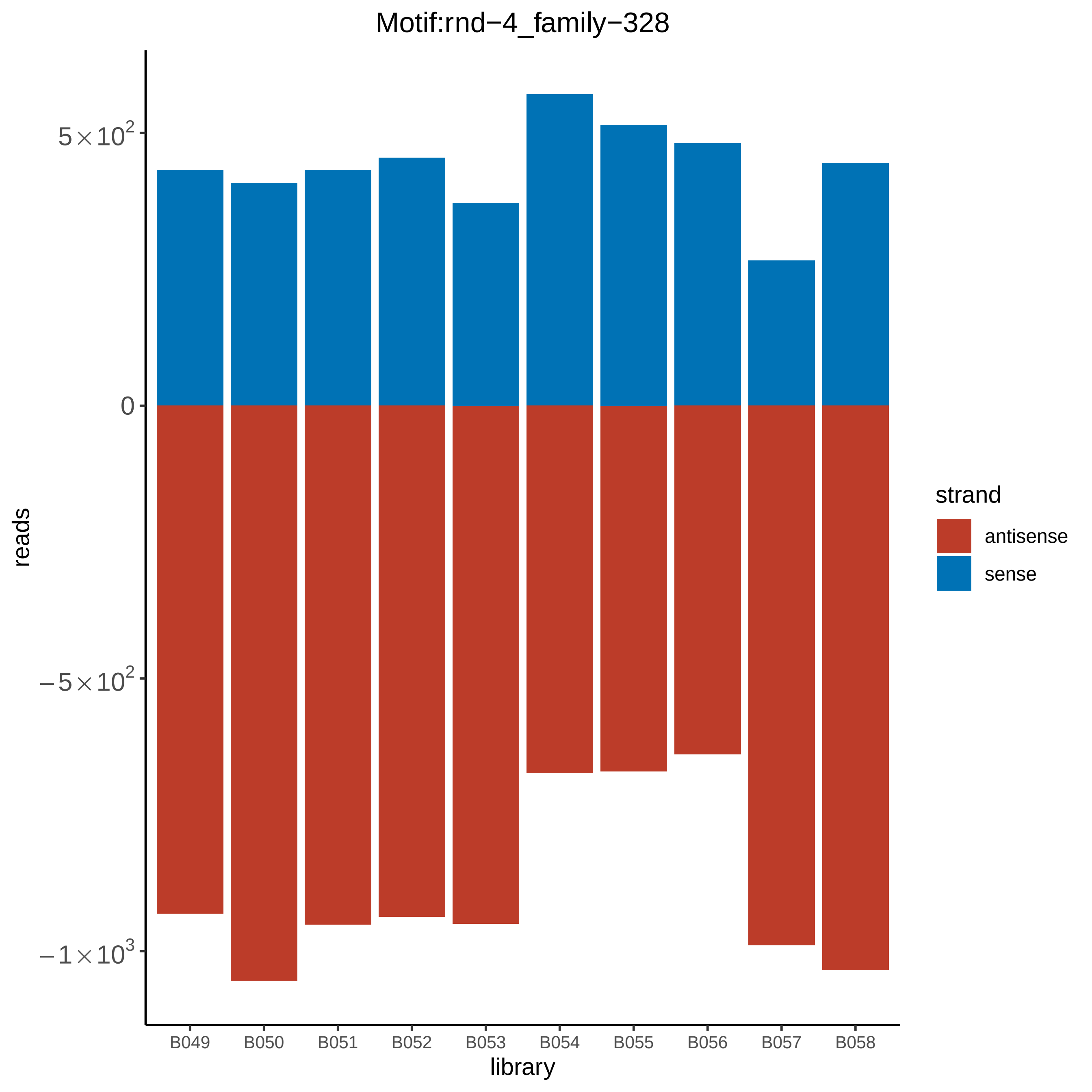
Motif:rnd−4_family−328
5×10^2
0
−5×10^2
−1×10^3
reads
B049
B050
B051
B052
B053
B054
B055
B056
B057
B058
library
strand
antisense
sense

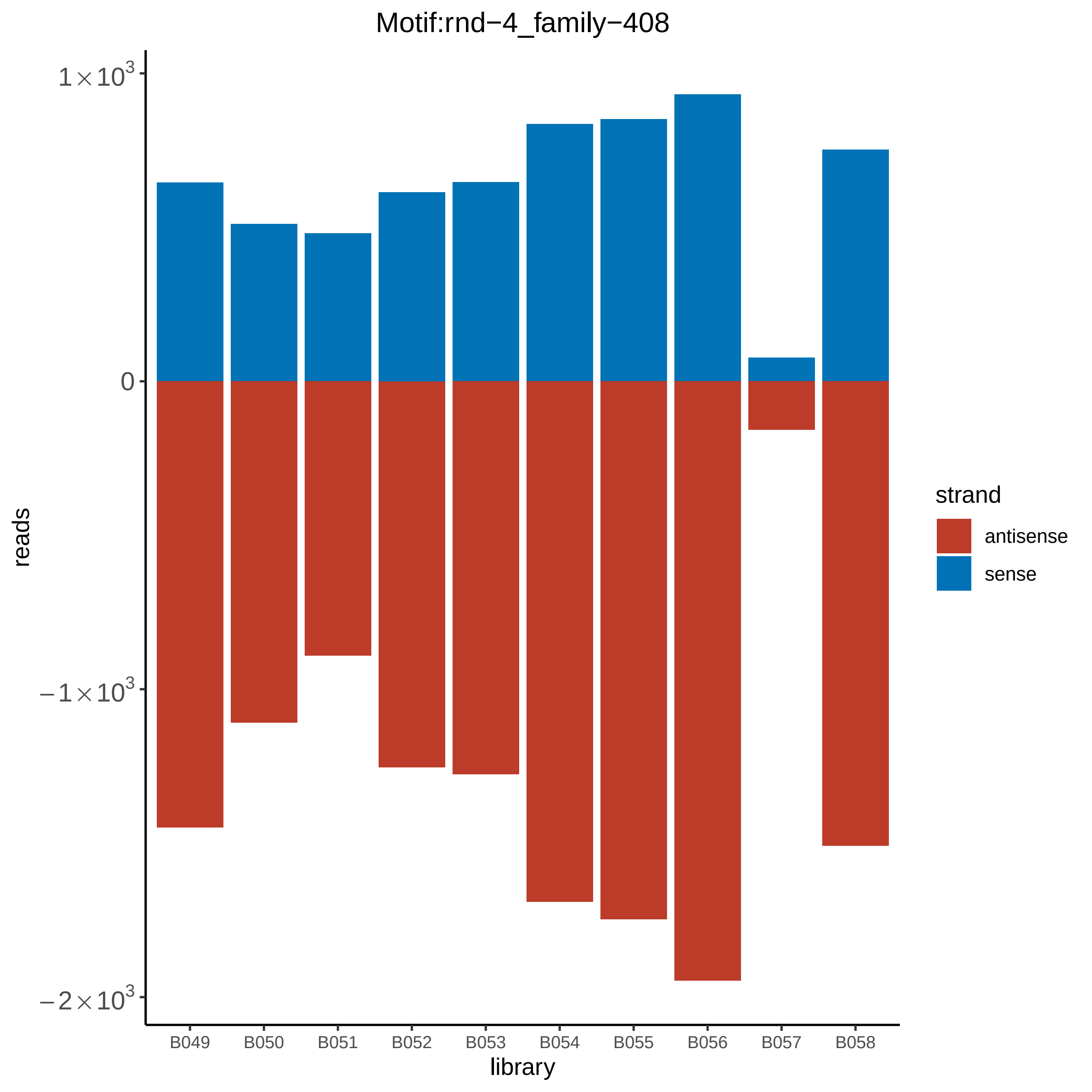
Motif:rnd−4_family−408
reads
1×10^3
0
−1×10^3
−2×10^3
library
B049
B050
B051
B052
B053
B054
B055
B056
B057
B058
strand
antisense
sense

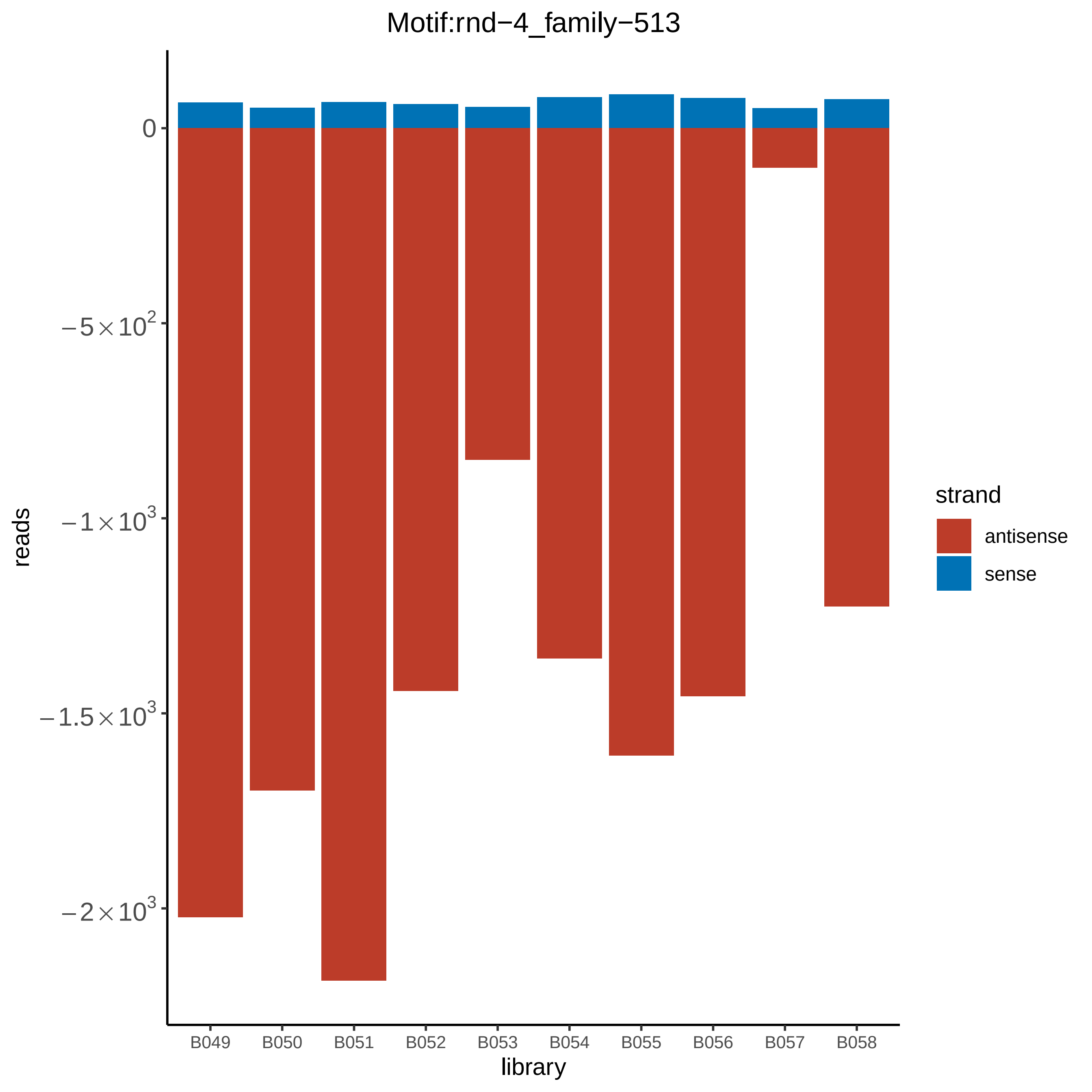

Motif:rnd−4_family−513
0
-5×10^2
-1×10^3
-1.5×10^3
-2×10^3
reads
B049
B050
B051
B052
B053
B054
B055
B056
B057
B058
library
strand
antisense
sense

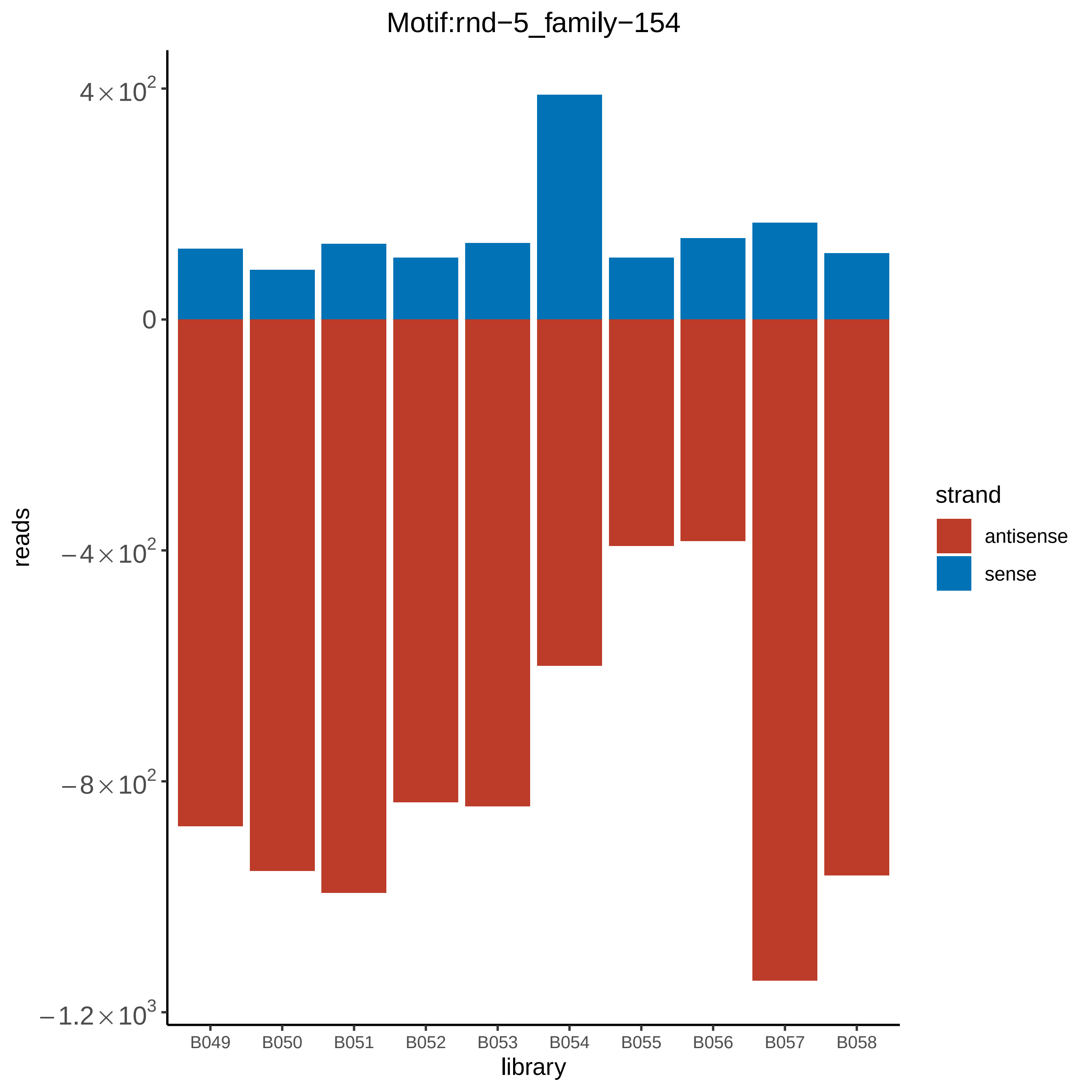
Motif:rnd−5_family−154
reads
library
strand
antisense
sense
4×10^2
0
-4×10^2
-8×10^2
-1.2×10^3
B049
B050
B051
B052
B053
B054
B055
B056
B057
B058

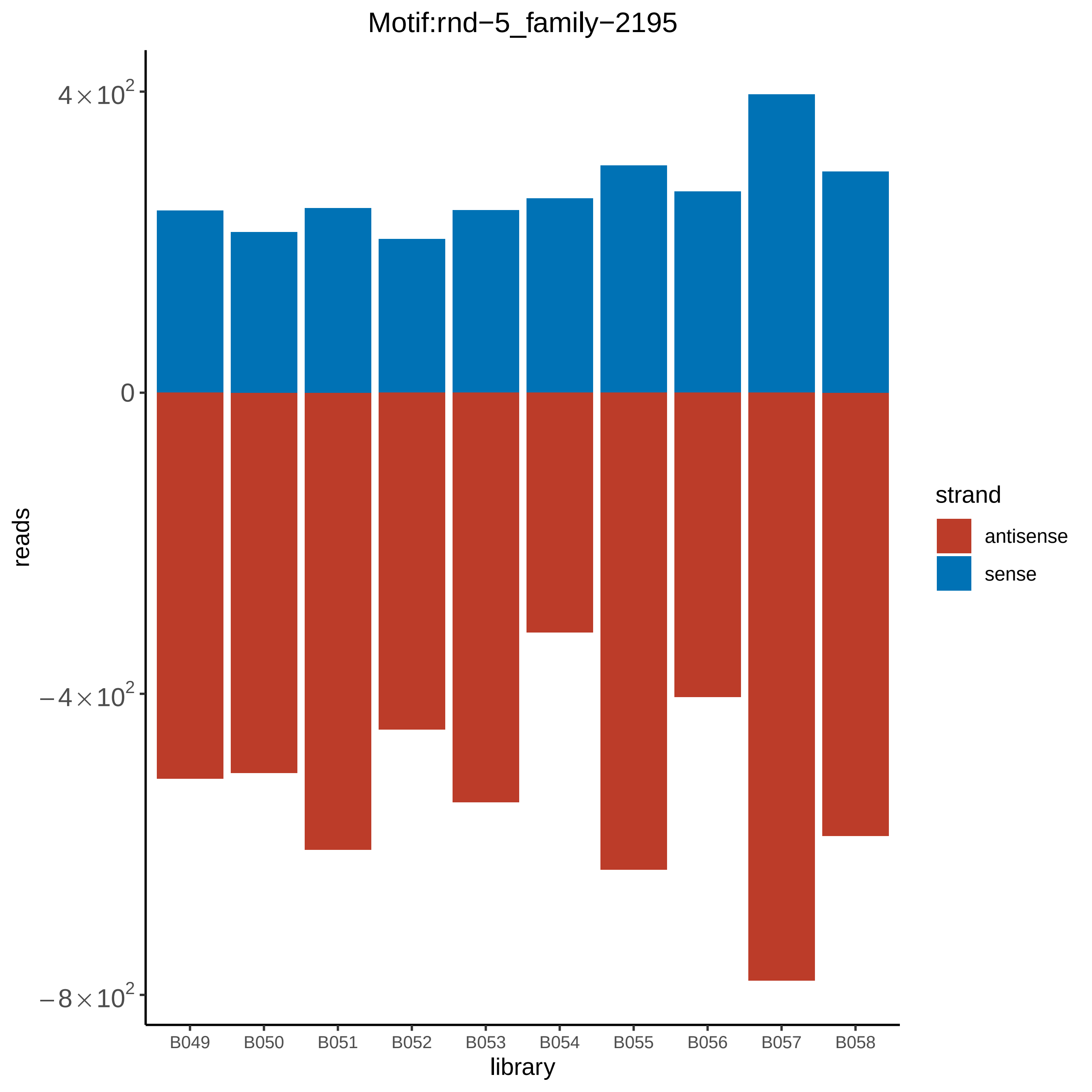
Motif:rnd−5_family−2195
4×10^2
0
−4×10^2
−8×10^2
reads
B049
B050
B051
B052
B053
B054
B055
B056
B057
B058
library
strand
antisense
sense

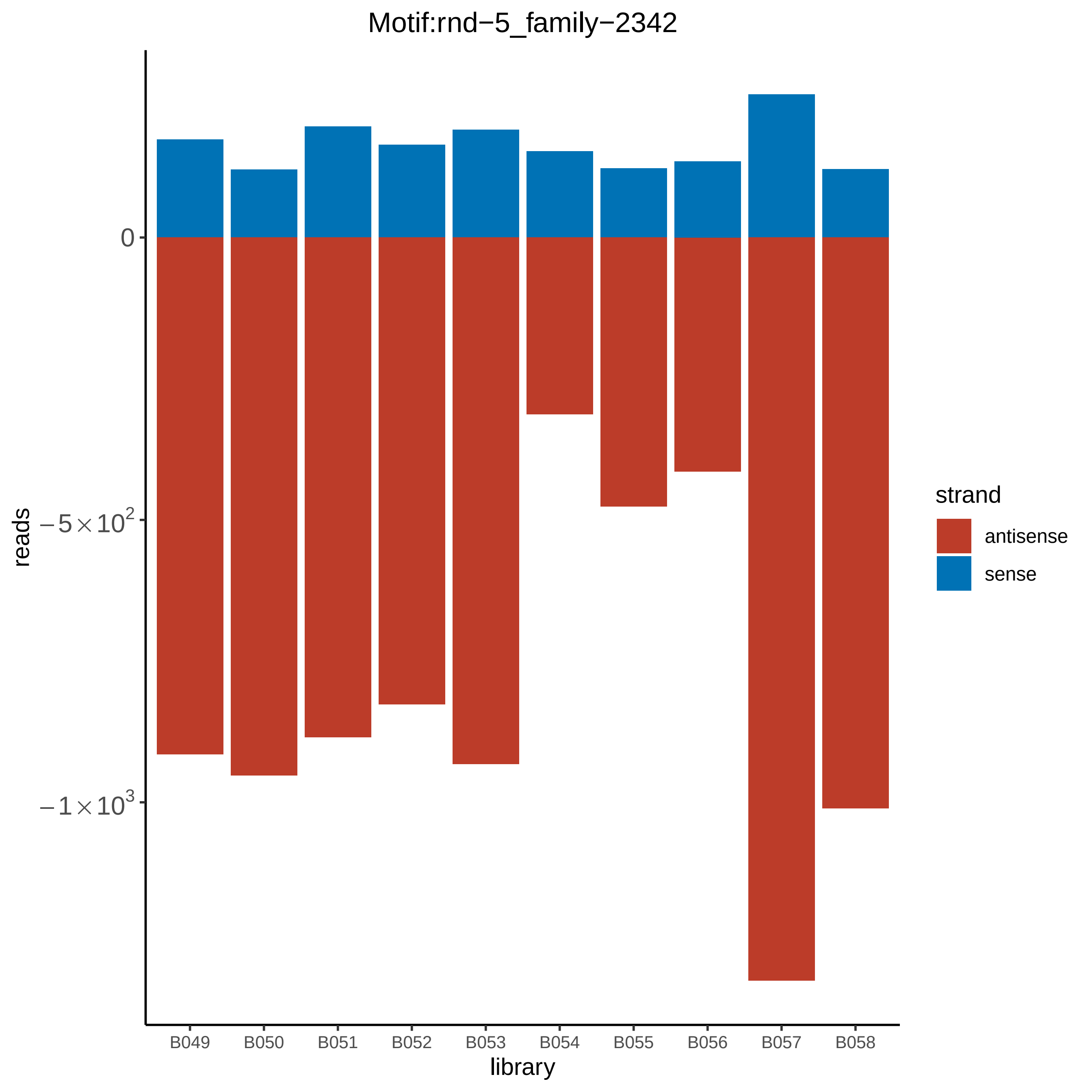

Motif:rnd−5_family−2342
reads
0
-5×10^2
-1×10^3
library
B049
B050
B051
B052
B053
B054
B055
B056
B057
B058
strand
antisense
sense

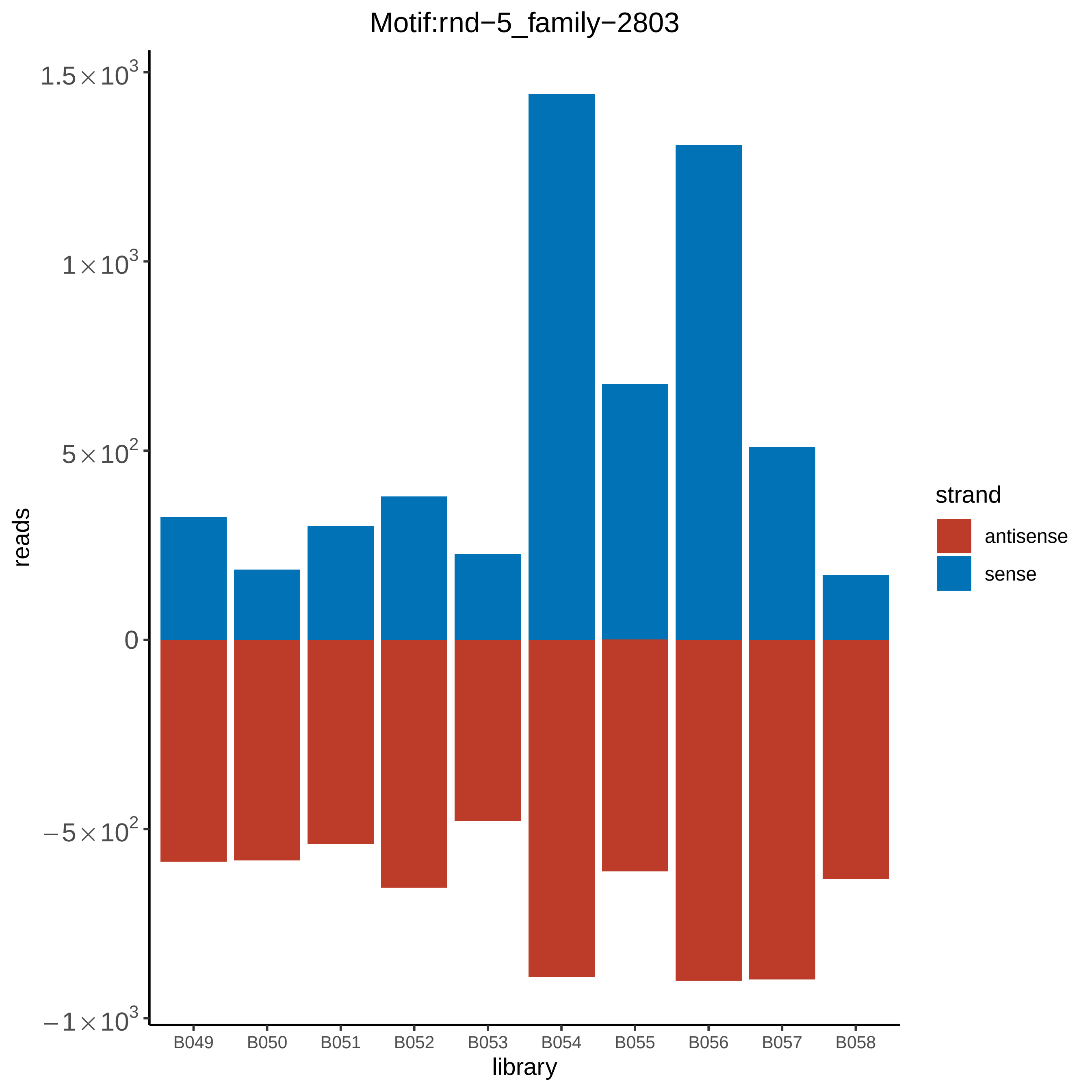

Motif:rnd−5_family−2803
reads
1.5×10^3
1×10^3
5×10^2
0
−5×10^2
−1×10^3
library
B049
B050
B051
B052
B053
B054
B055
B056
B057
B058
strand
antisense
sense

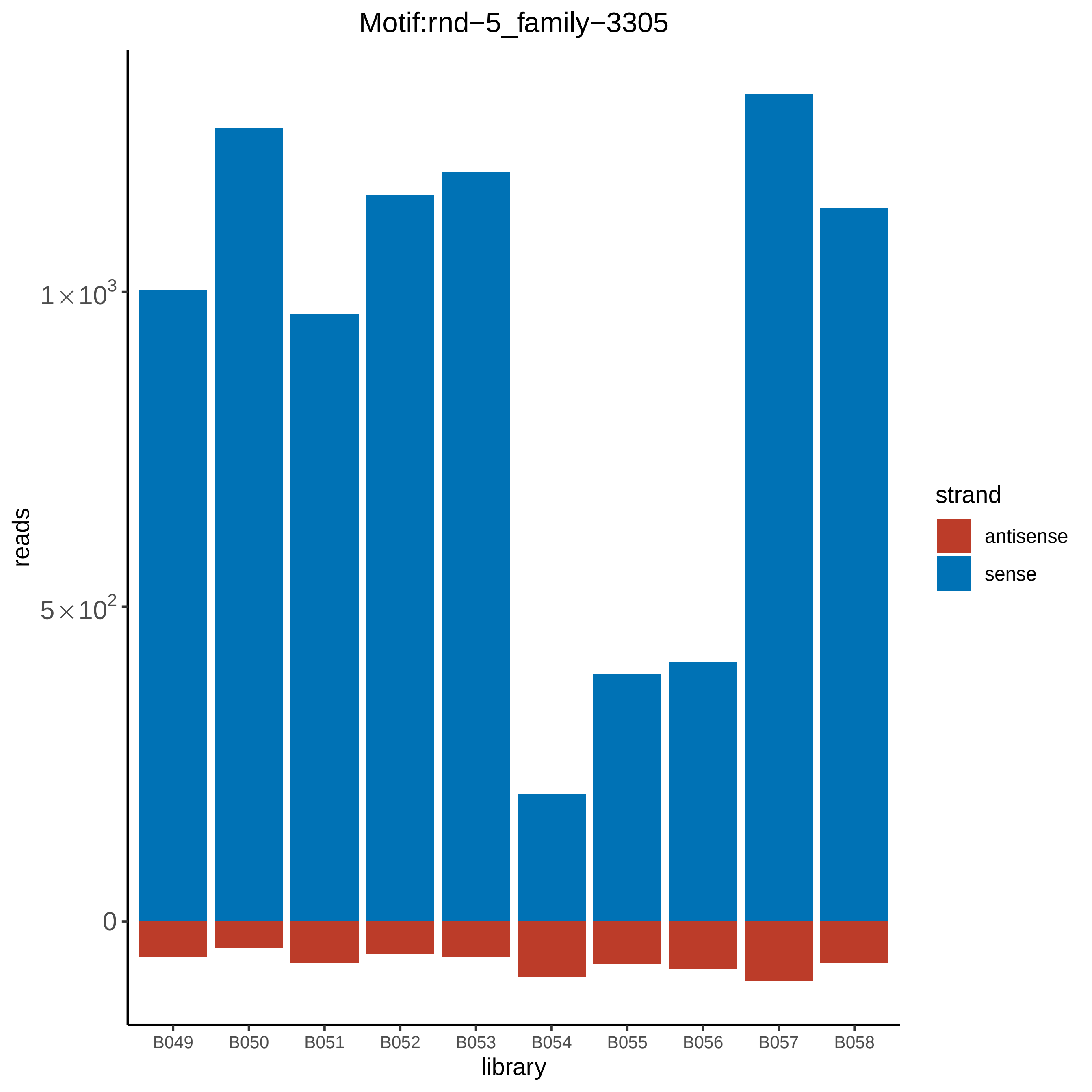

Motif:rnd−5_family−3305
reads
1×10^3
5×10^2
0
library
B049
B050
B051
B052
B053
B054
B055
B056
B057
B058
strand
antisense
sense

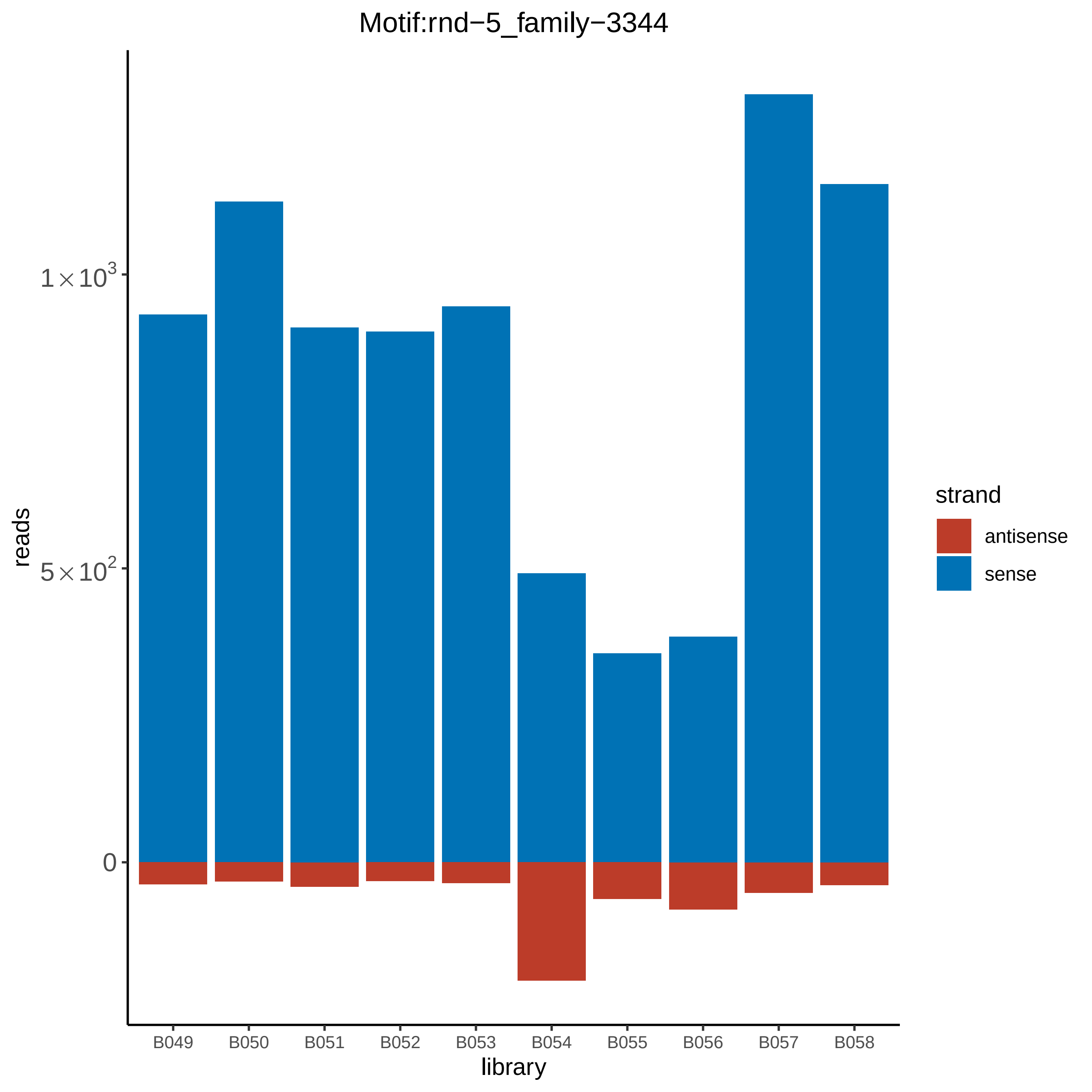

Motif:rnd−5_family−3344
reads
1×10^3
5×10^2
0
library
B049
B050
B051
B052
B053
B054
B055
B056
B057
B058
strand
antisense
sense

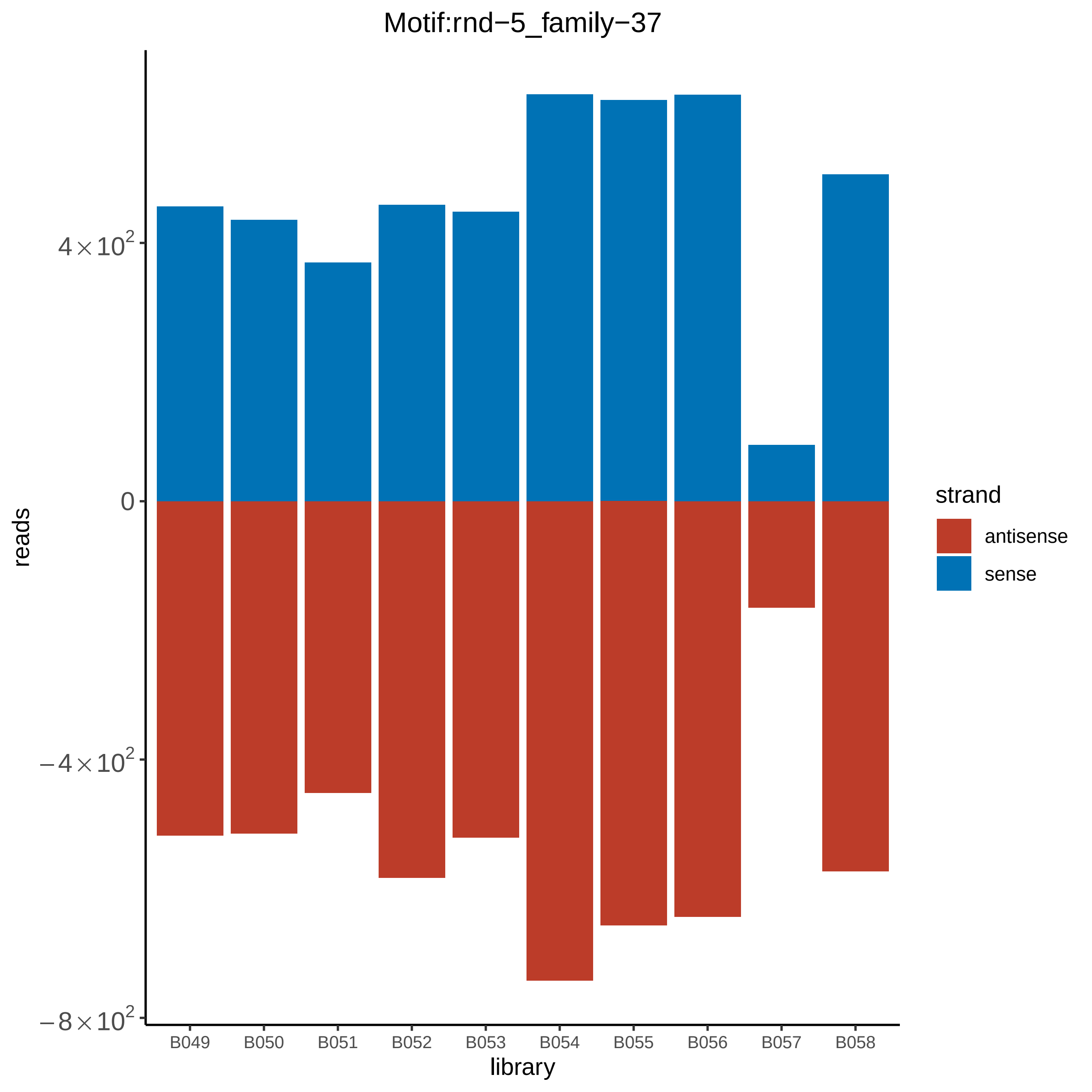

Motif:rnd−5_family−37
4×10^2
0
−4×10^2
−8×10^2
reads
B049
B050
B051
B052
B053
B054
B055
B056
B057
B058
library
strand
antisense
sense

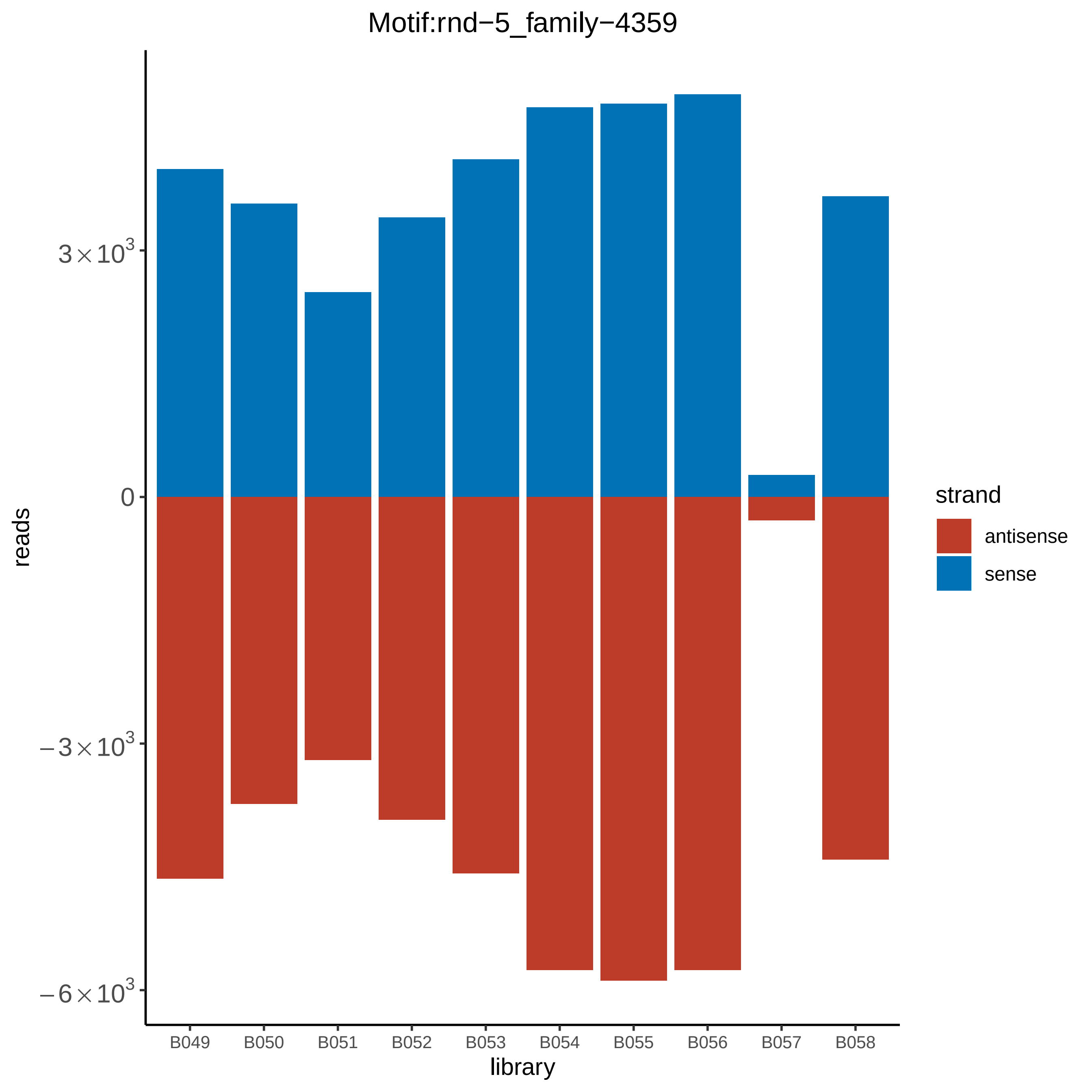
Motif:rnd−5_family−4359
reads
3×10^3
0
-3×10^3
-6×10^3
B049
B050
B051
B052
B053
B054
B055
B056
B057
B058
library
strand
antisense
sense

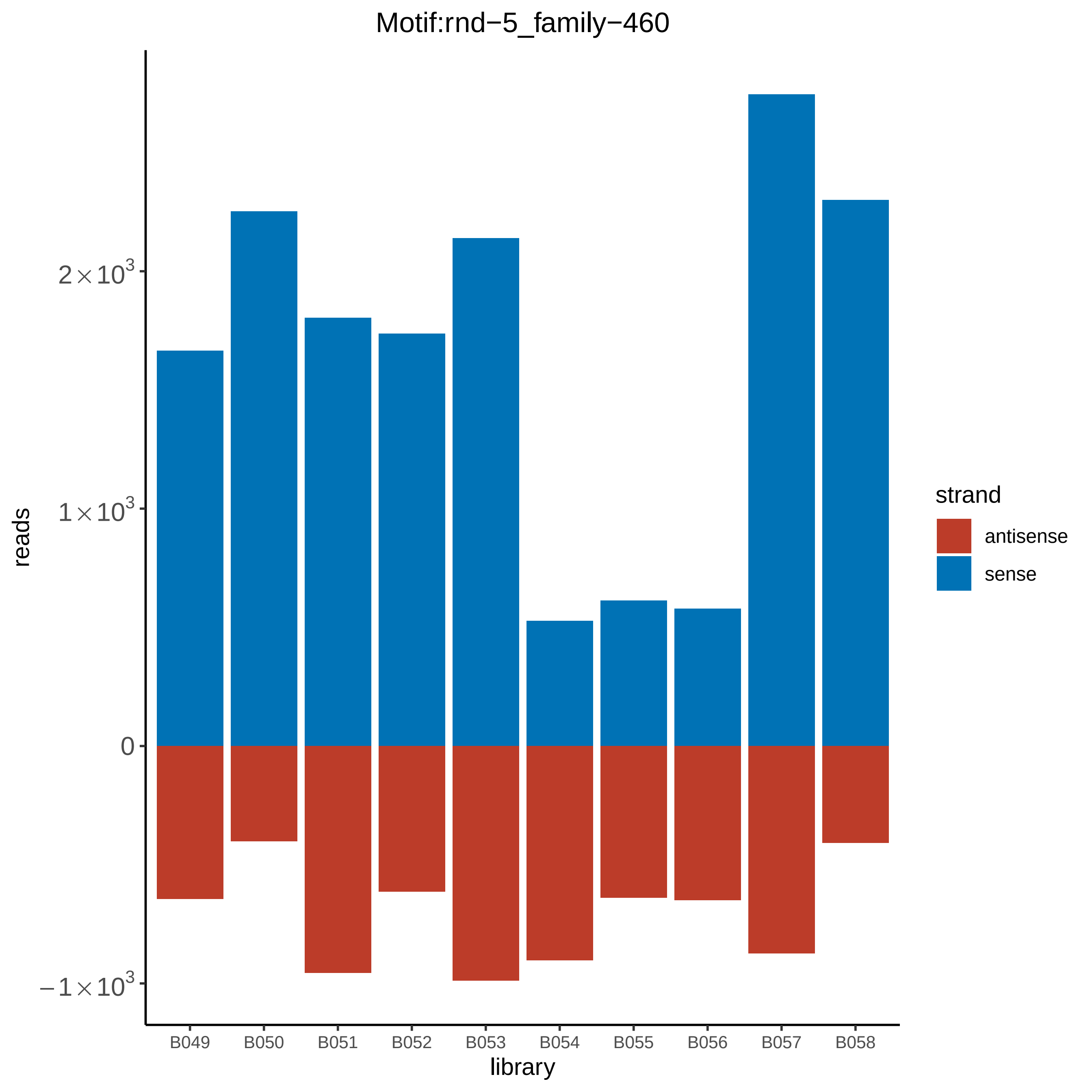

Motif:rnd−5_family−460
reads
2×10^3
1×10^3
0
-1×10^3
B049
B050
B051
B052
B053
B054
B055
B056
B057
B058
library
strand
antisense
sense

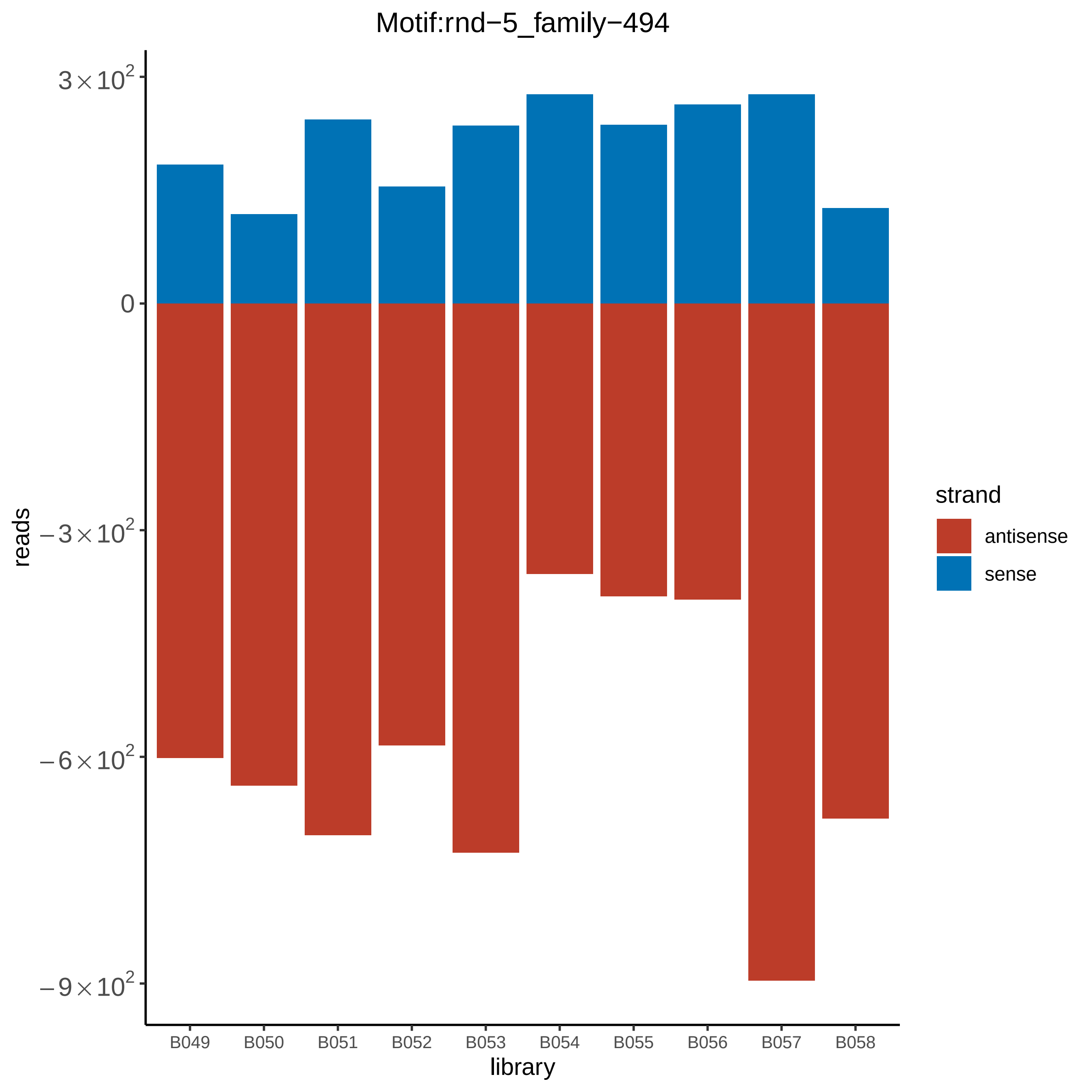

Motif:rnd−5_family−494
3×10^2
0
−3×10^2
−6×10^2
−9×10^2
reads
B049
B050
B051
B052
B053
B054
B055
B056
B057
B058
library
strand
antisense
sense

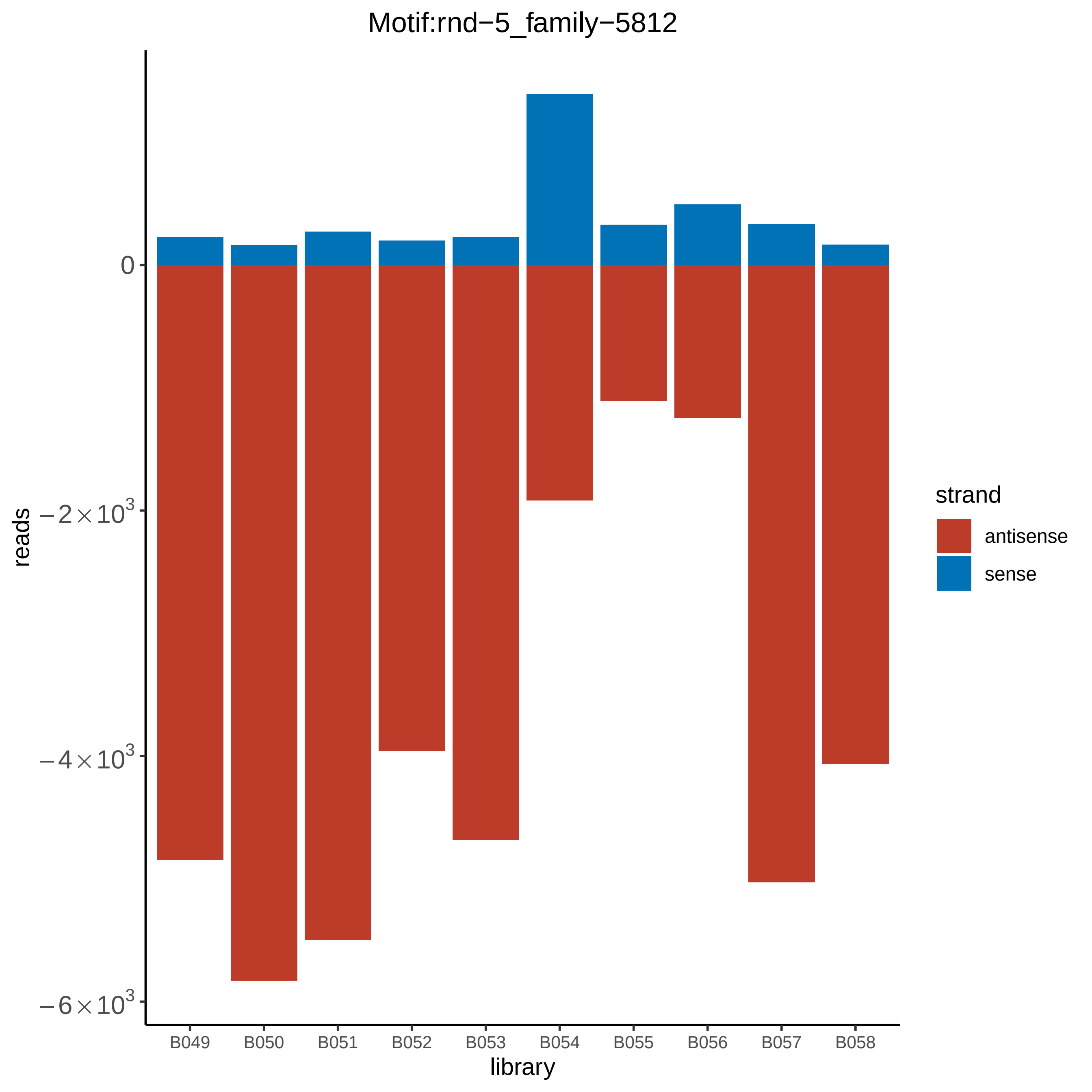

Motif:rnd−5_family−5812
reads
0
−2×10^3
−4×10^3
−6×10^3
library
B049
B050
B051
B052
B053
B054
B055
B056
B057
B058
strand
antisense
sense

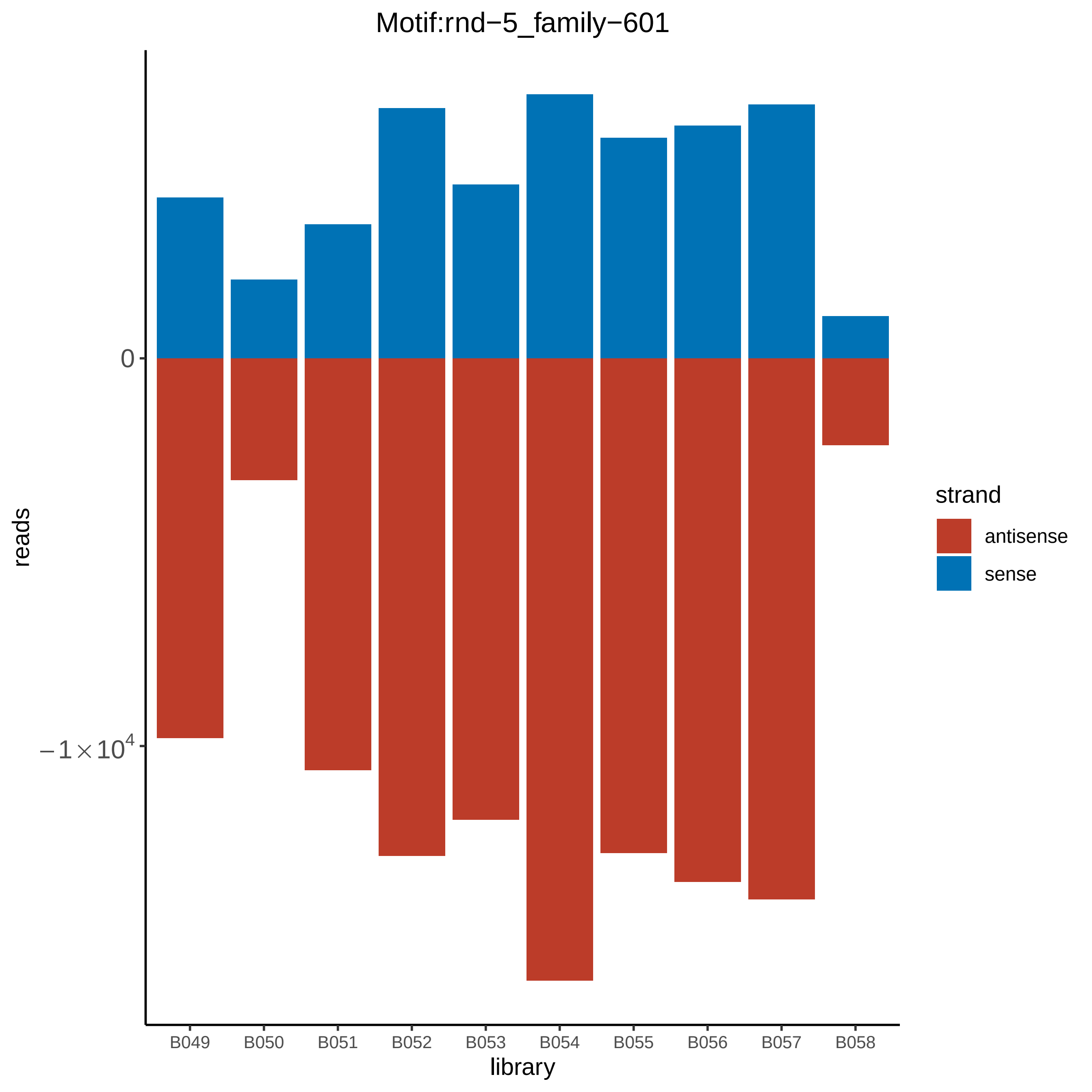
Motif:rnd−5_family−601
reads
0
-1×10^4
library
B049
B050
B051
B052
B053
B054
B055
B056
B057
B058
strand
antisense
sense

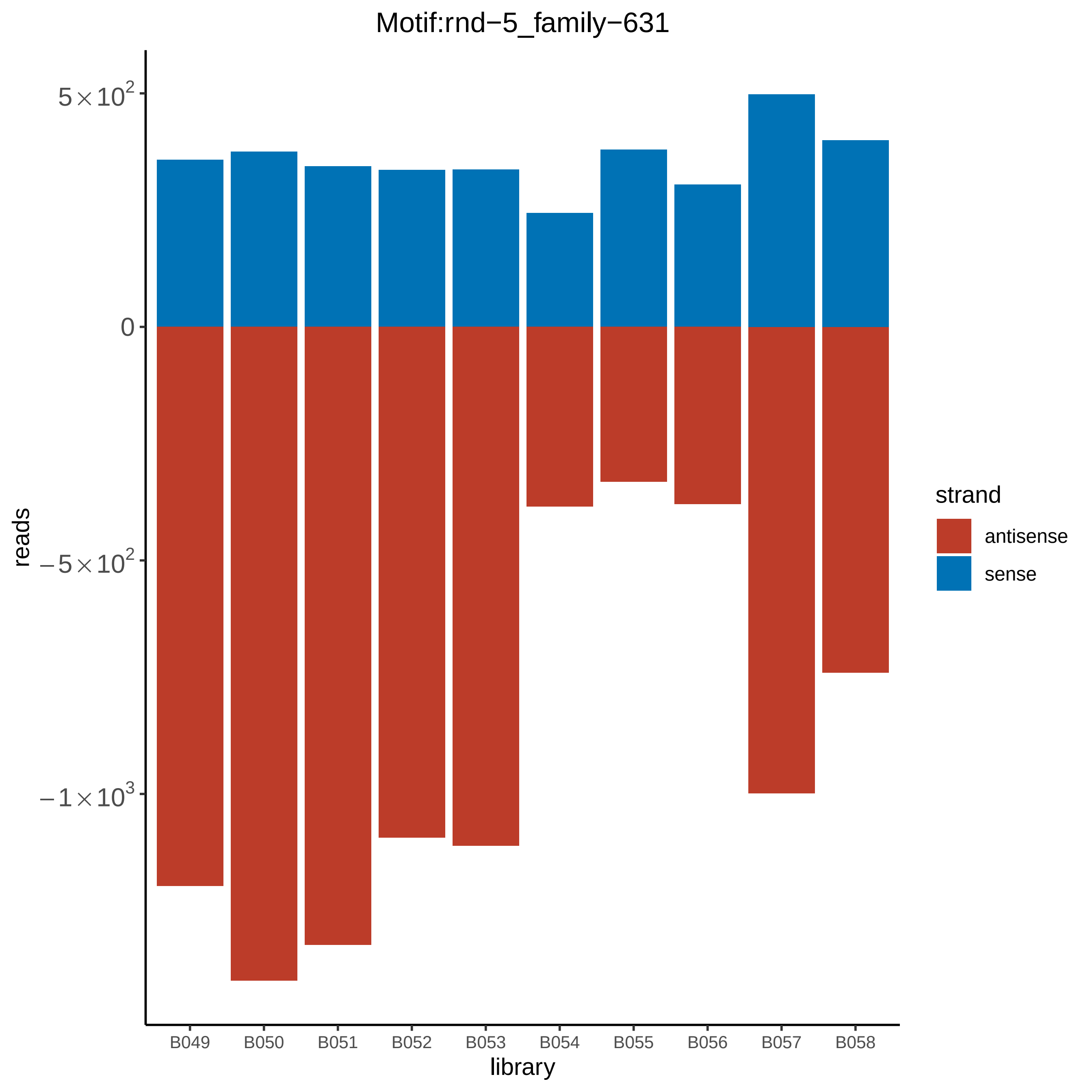

Motif:rnd−5_family−631
reads
5×10^2
0
-5×10^2
-1×10^3
B049
B050
B051
B052
B053
B054
B055
B056
B057
B058
library
strand
antisense
sense

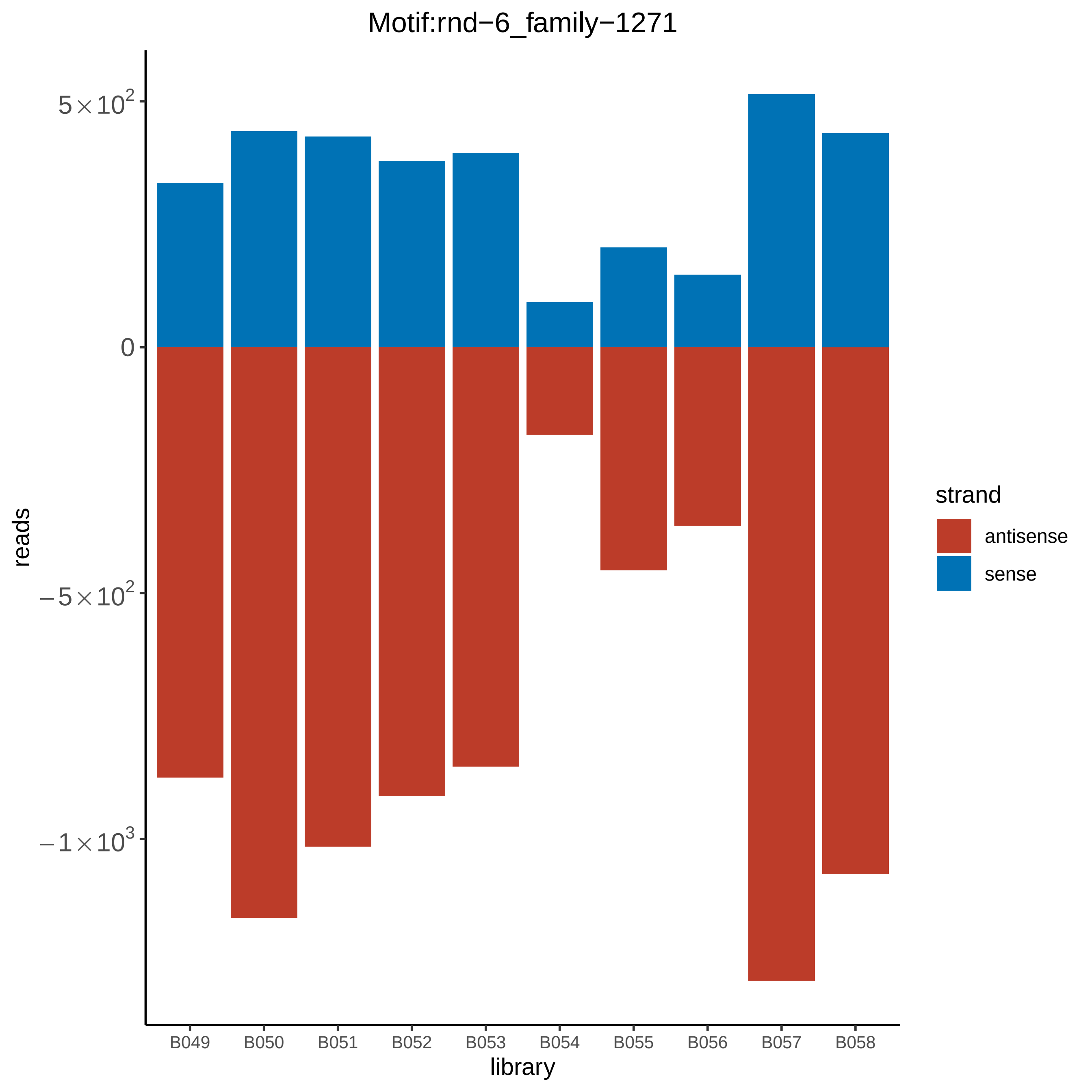

Motif:rnd−6_family−1271
5×10^2
0
−5×10^2
−1×10^3
reads
B049
B050
B051
B052
B053
B054
B055
B056
B057
B058
library
strand
antisense
sense

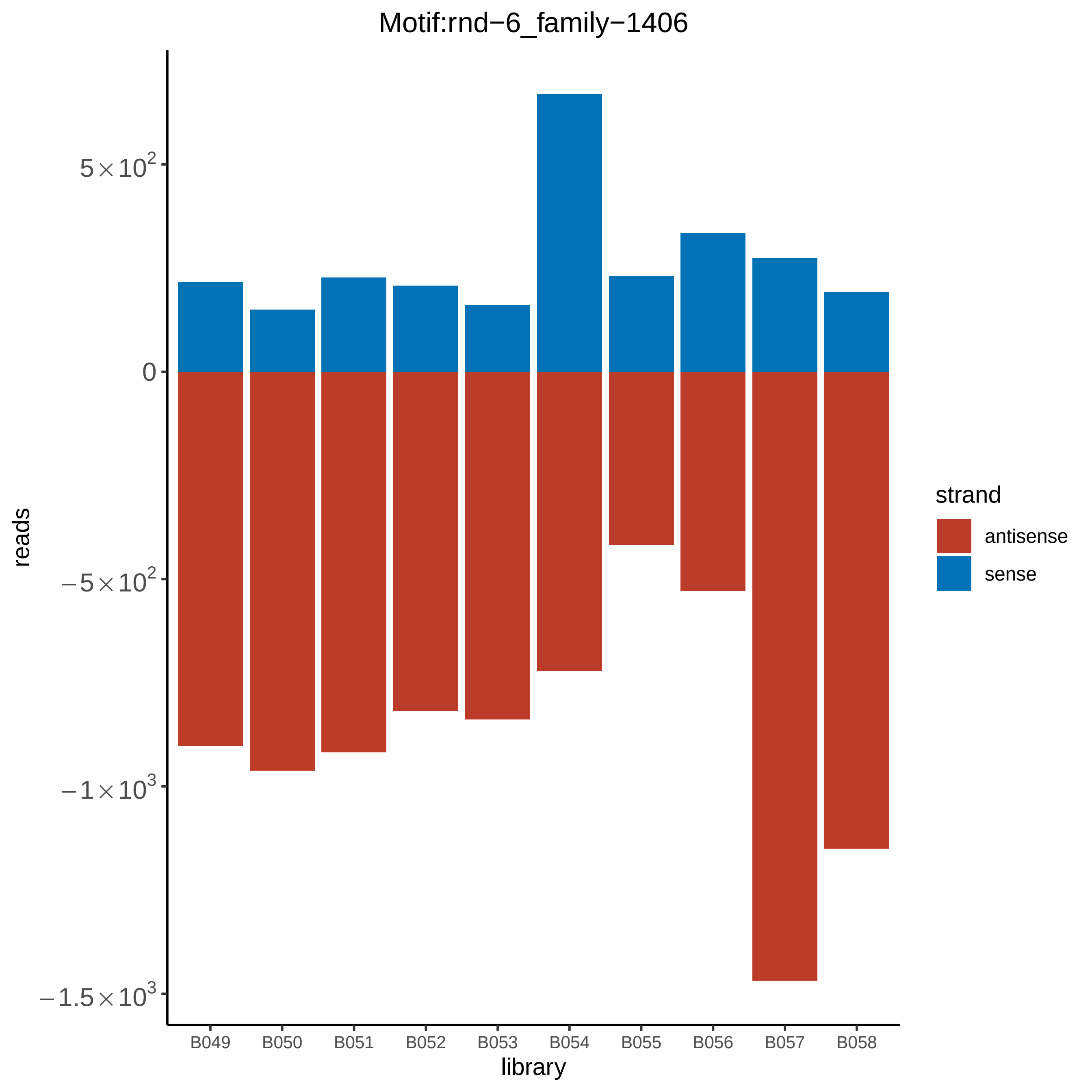

Motif:rnd−6_family−1406
reads
5×10^2
0
−5×10^2
−1×10^3
−1.5×10^3
B049
B050
B051
B052
B053
B054
B055
B056
B057
B058
library
strand
antisense
sense

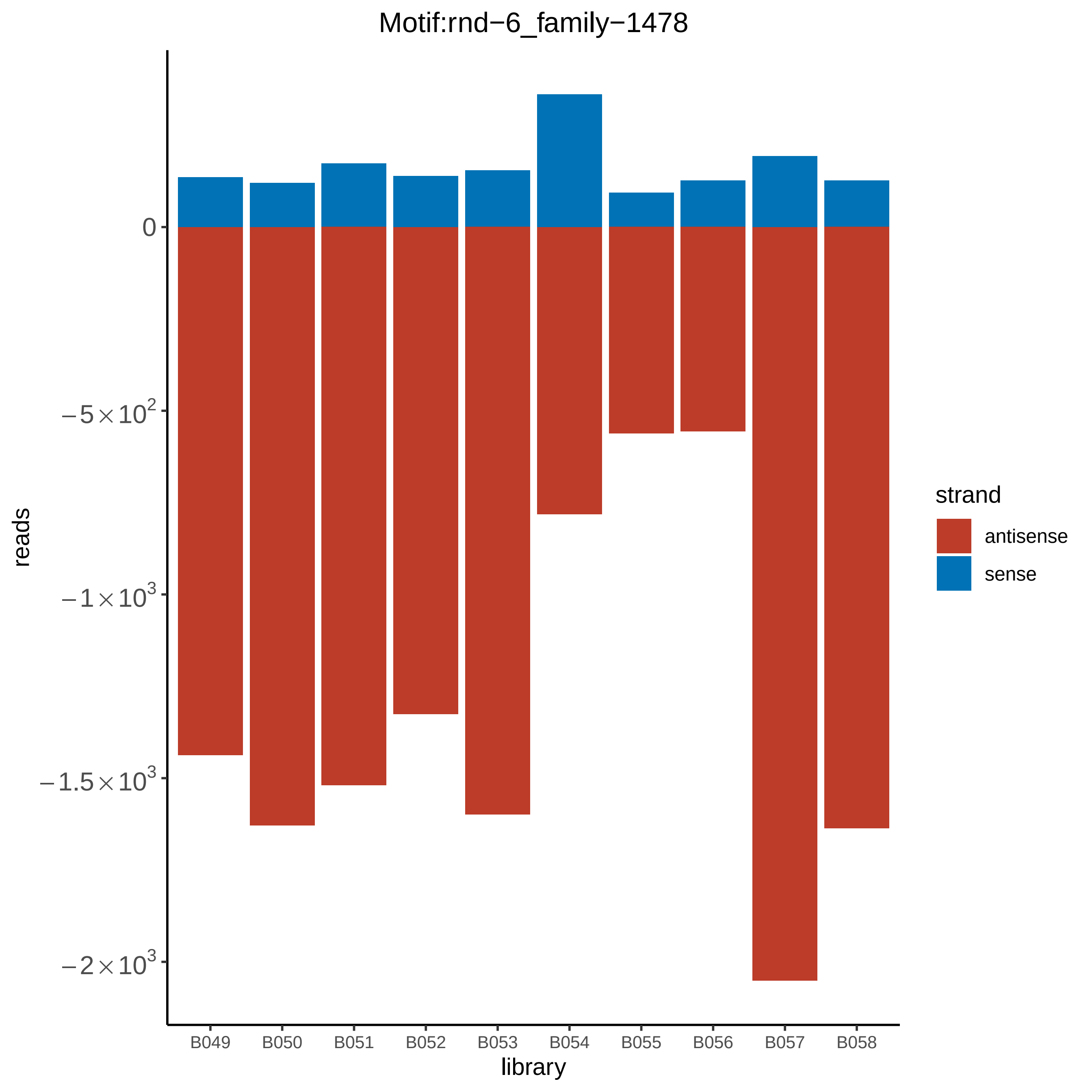

Motif:rnd−6_family−1478
0
−5×10^2
−1×10^3
−1.5×10^3
−2×10^3
reads
B049
B050
B051
B052
B053
B054
B055
B056
B057
B058
library
strand
antisense
sense

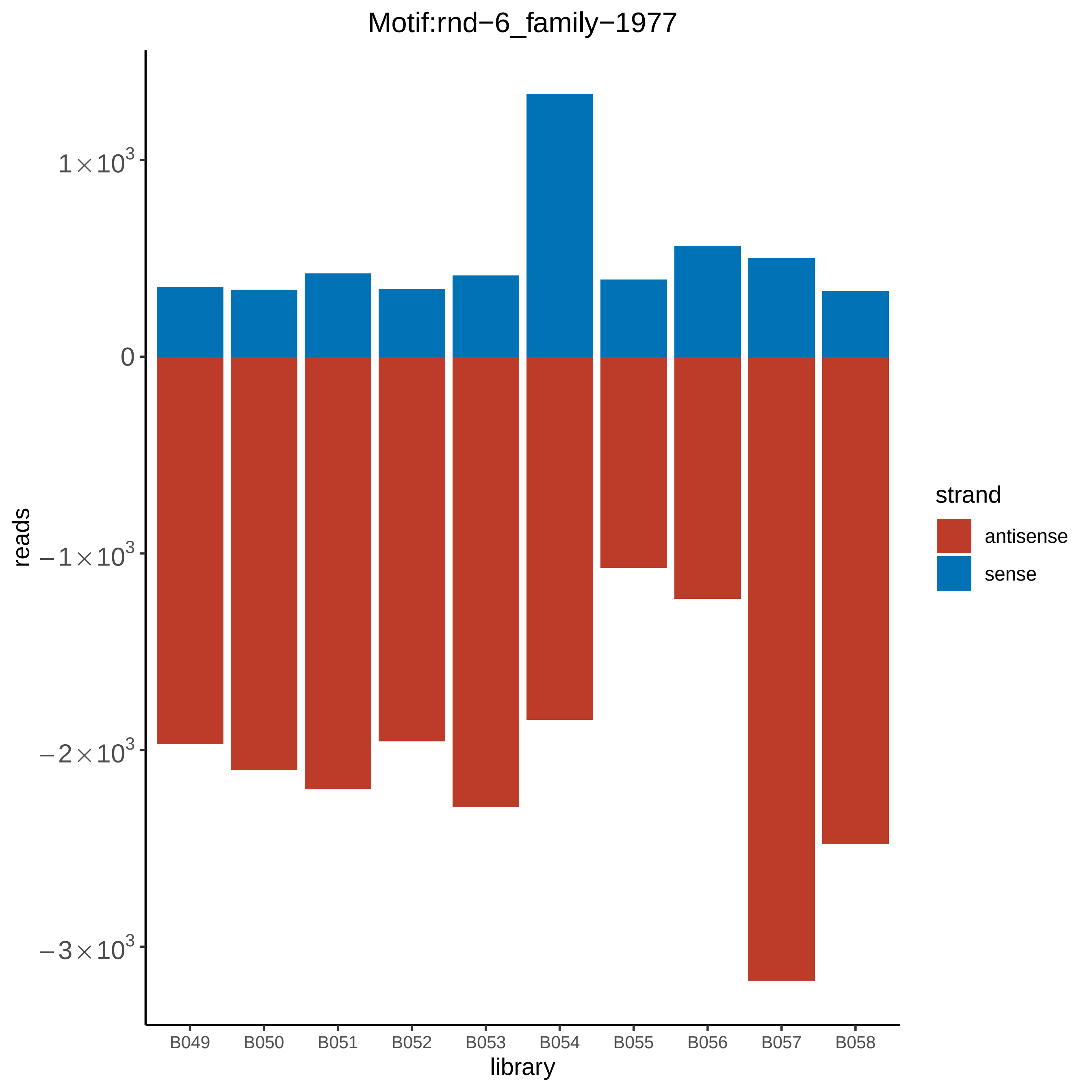

Motif:rnd−6_family−1977
1×10^3
0
-1×10^3
-2×10^3
-3×10^3
reads
B049
B050
B051
B052
B053
B054
B055
B056
B057
B058
library
strand
antisense
sense

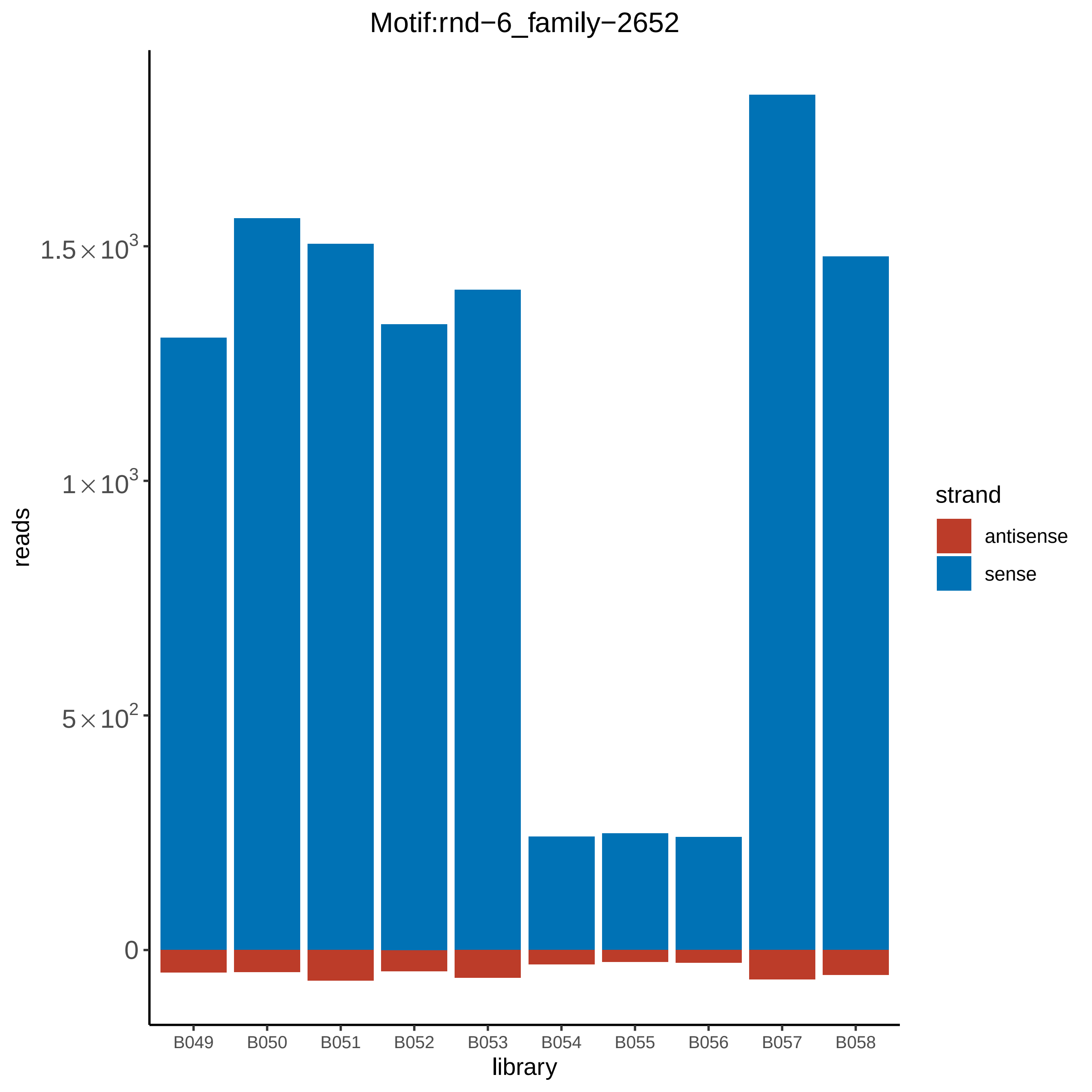

Motif:rnd−6_family−2652
reads
1.5×10^3
1×10^3
5×10^2
0
library
B049
B050
B051
B052
B053
B054
B055
B056
B057
B058
strand
antisense
sense

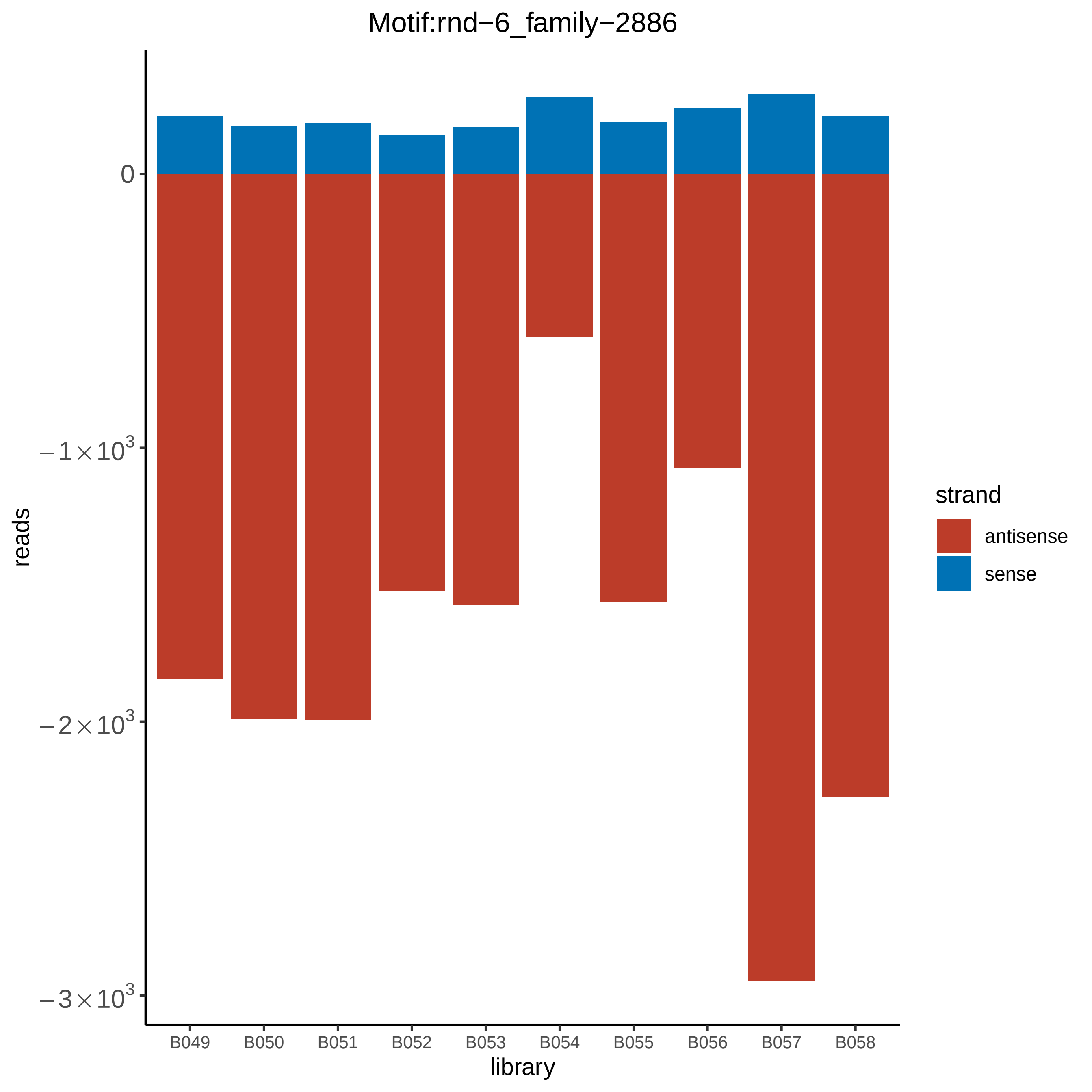
Motif:rnd−6_family−2886
0
-1×10^3
-2×10^3
-3×10^3
reads
B049
B050
B051
B052
B053
B054
B055
B056
B057
B058
library
strand
antisense
sense

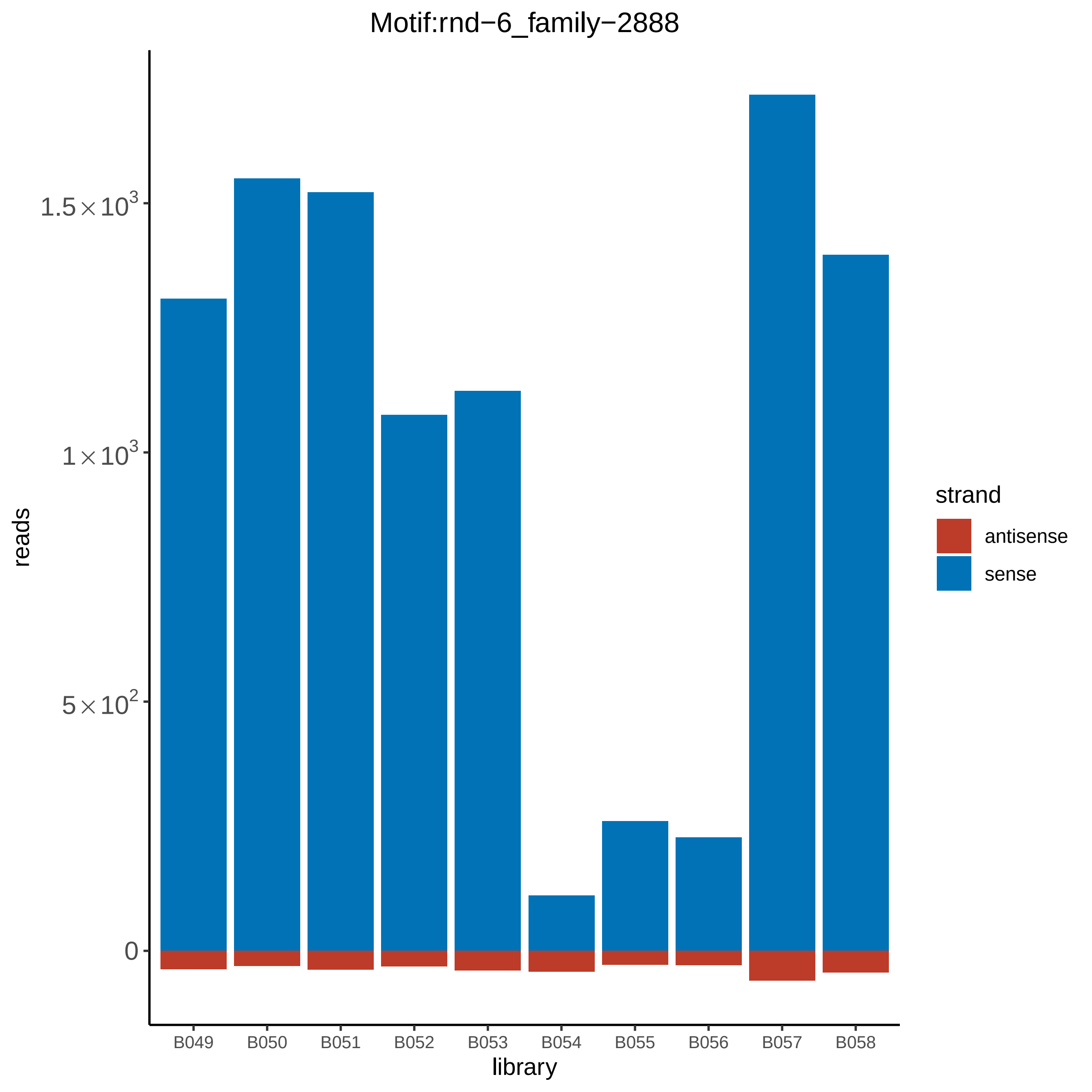
Motif:rnd−6_family−2888
reads
1.5×10^3
1×10^3
5×10^2
0
library
B049
B050
B051
B052
B053
B054
B055
B056
B057
B058
strand
antisense
sense

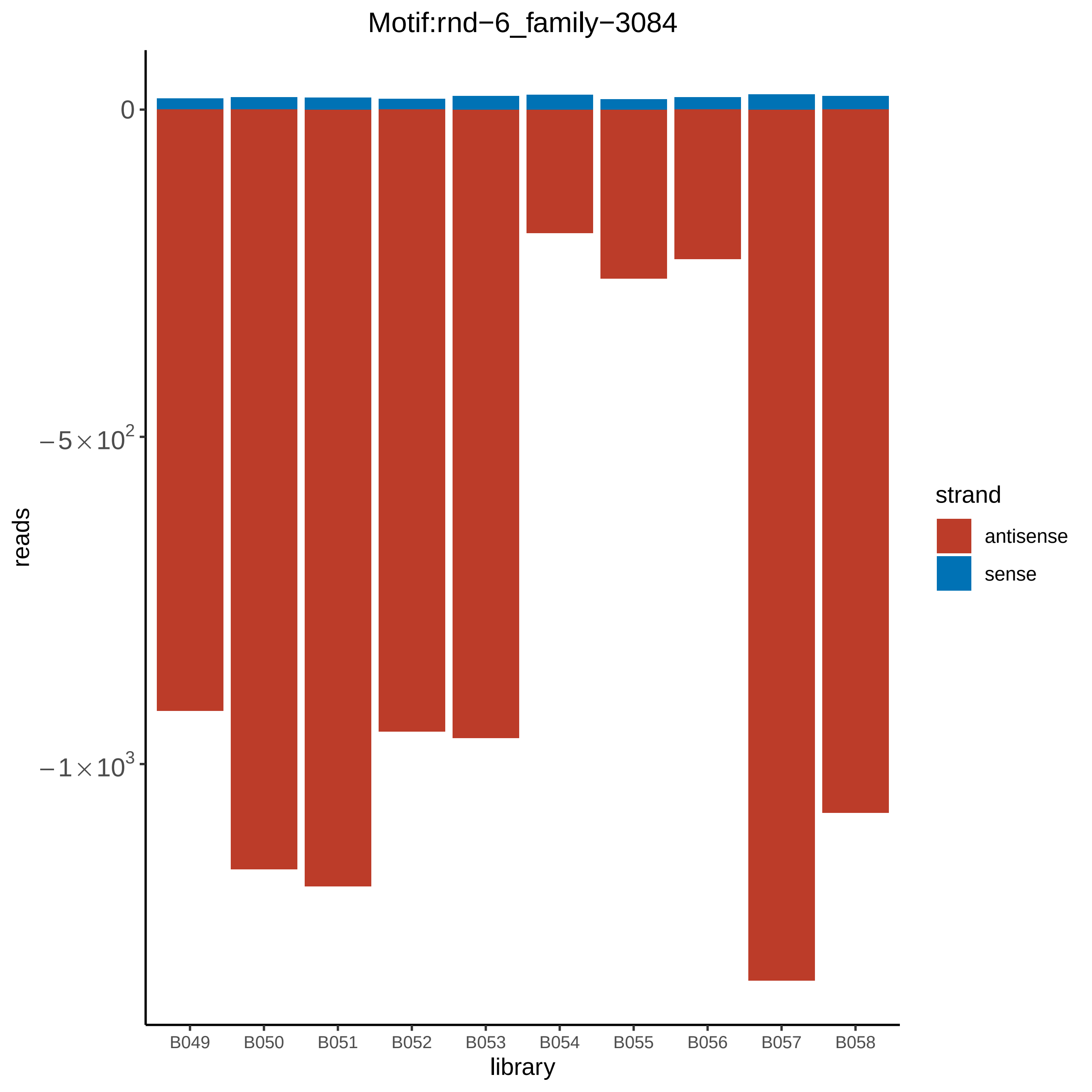

Motif:rnd−6_family−3084
0
-5×10^2
-1×10^3
reads
B049
B050
B051
B052
B053
B054
B055
B056
B057
B058
library
strand
antisense
sense

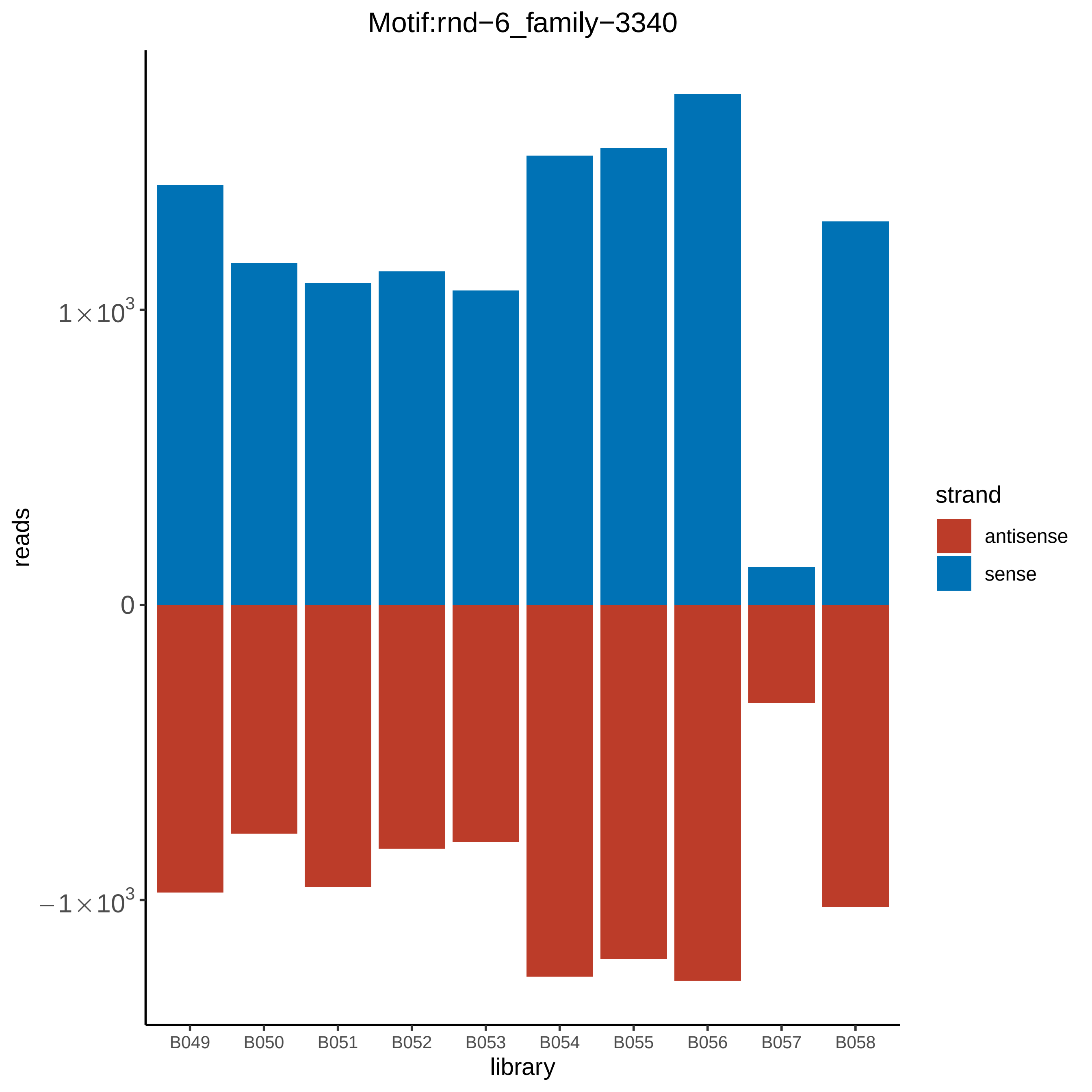
Motif:rnd−6_family−3340
reads
1×10^3
0
-1×10^3
B049
B050
B051
B052
B053
B054
B055
B056
B057
B058
library
strand
antisense
sense

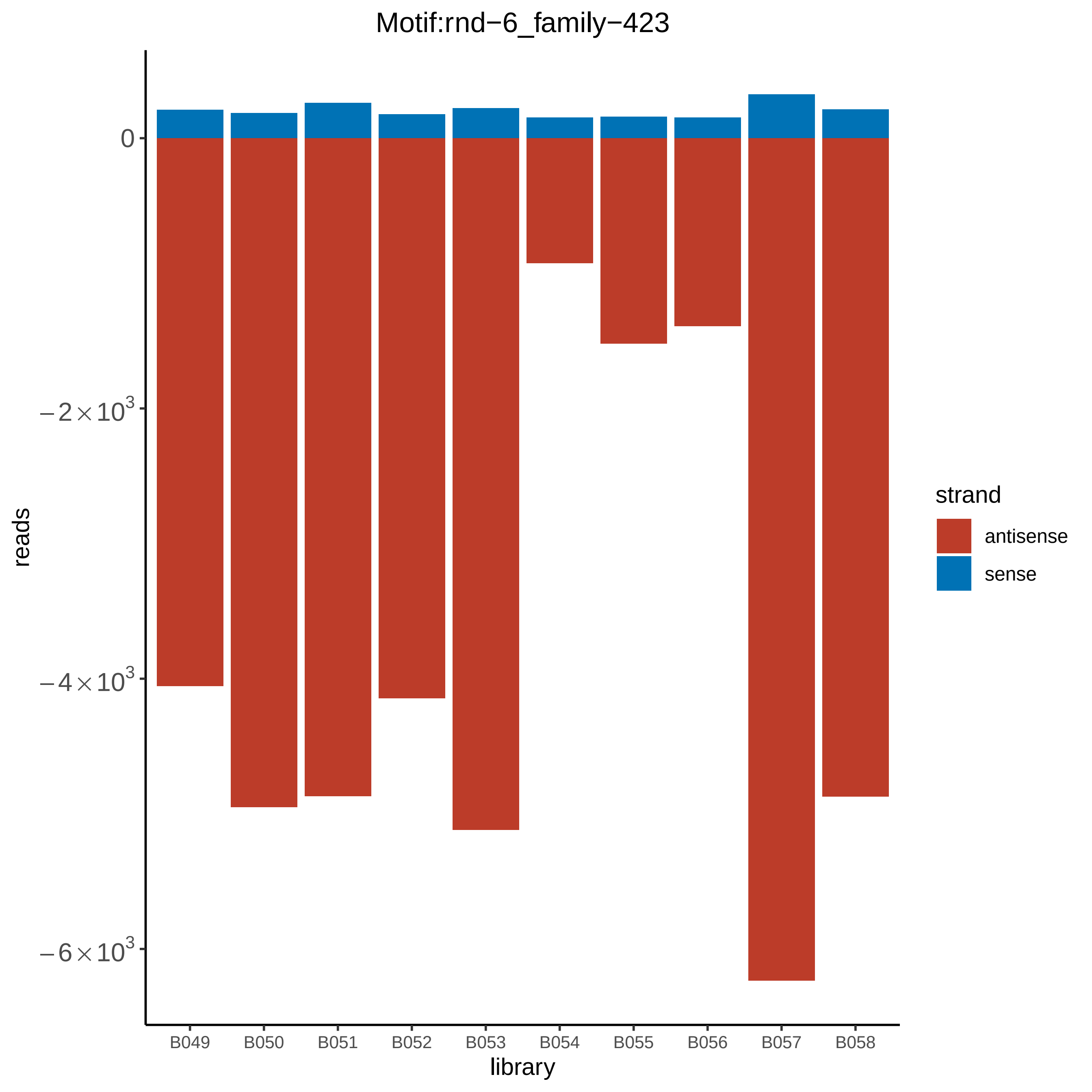

Motif:rnd−6_family−423
0
−2×10^3
−4×10^3
−6×10^3
reads
B049
B050
B051
B052
B053
B054
B055
B056
B057
B058
library
strand
antisense
sense

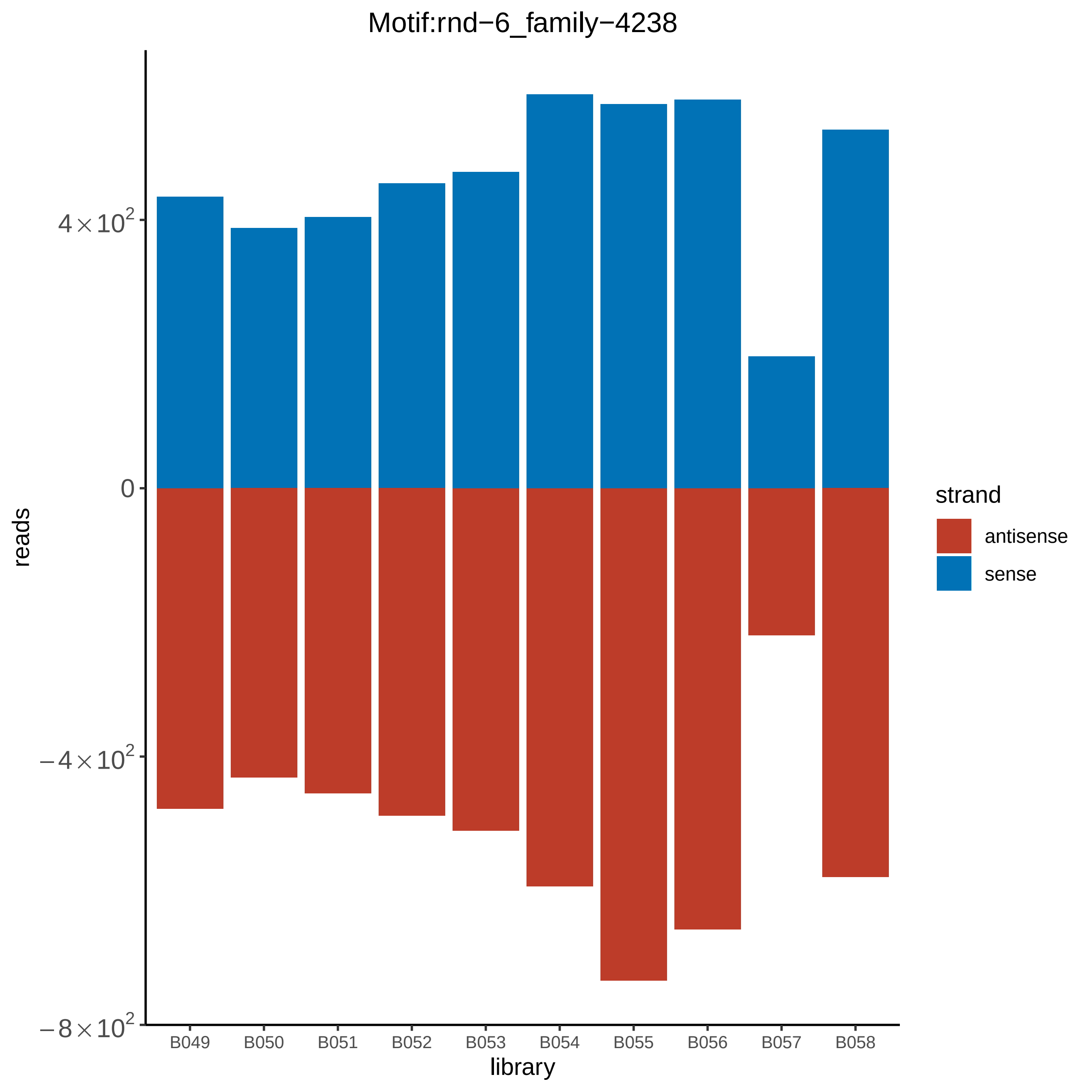

Motif:rnd−6_family−4238
reads
4×10^2
0
−4×10^2
−8×10^2
library
B049
B050
B051
B052
B053
B054
B055
B056
B057
B058
strand
antisense
sense

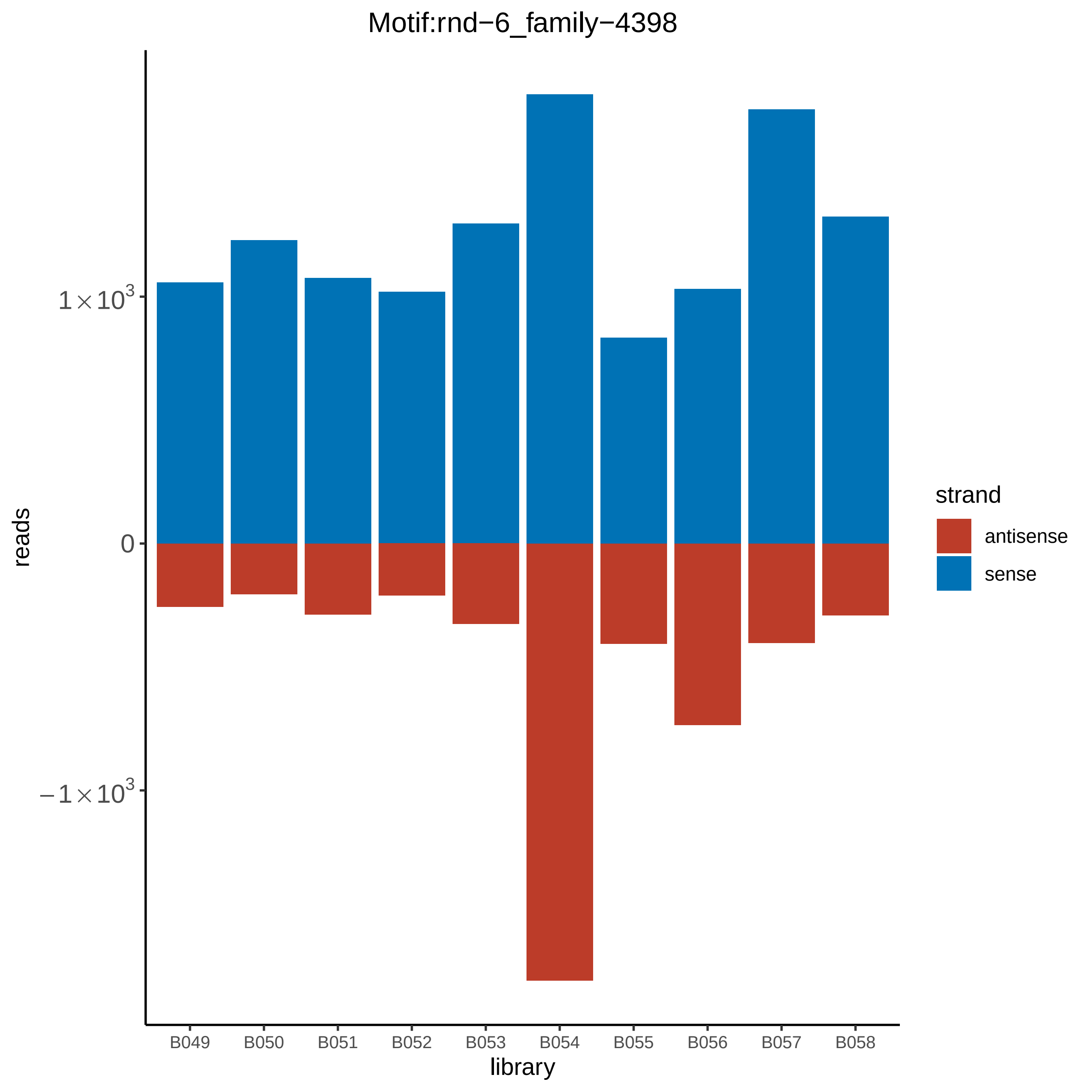
Motif:rnd−6_family−4398
reads
1×10^3
0
−1×10^3
B049
B050
B051
B052
B053
B054
B055
B056
B057
B058
library
strand
antisense
sense

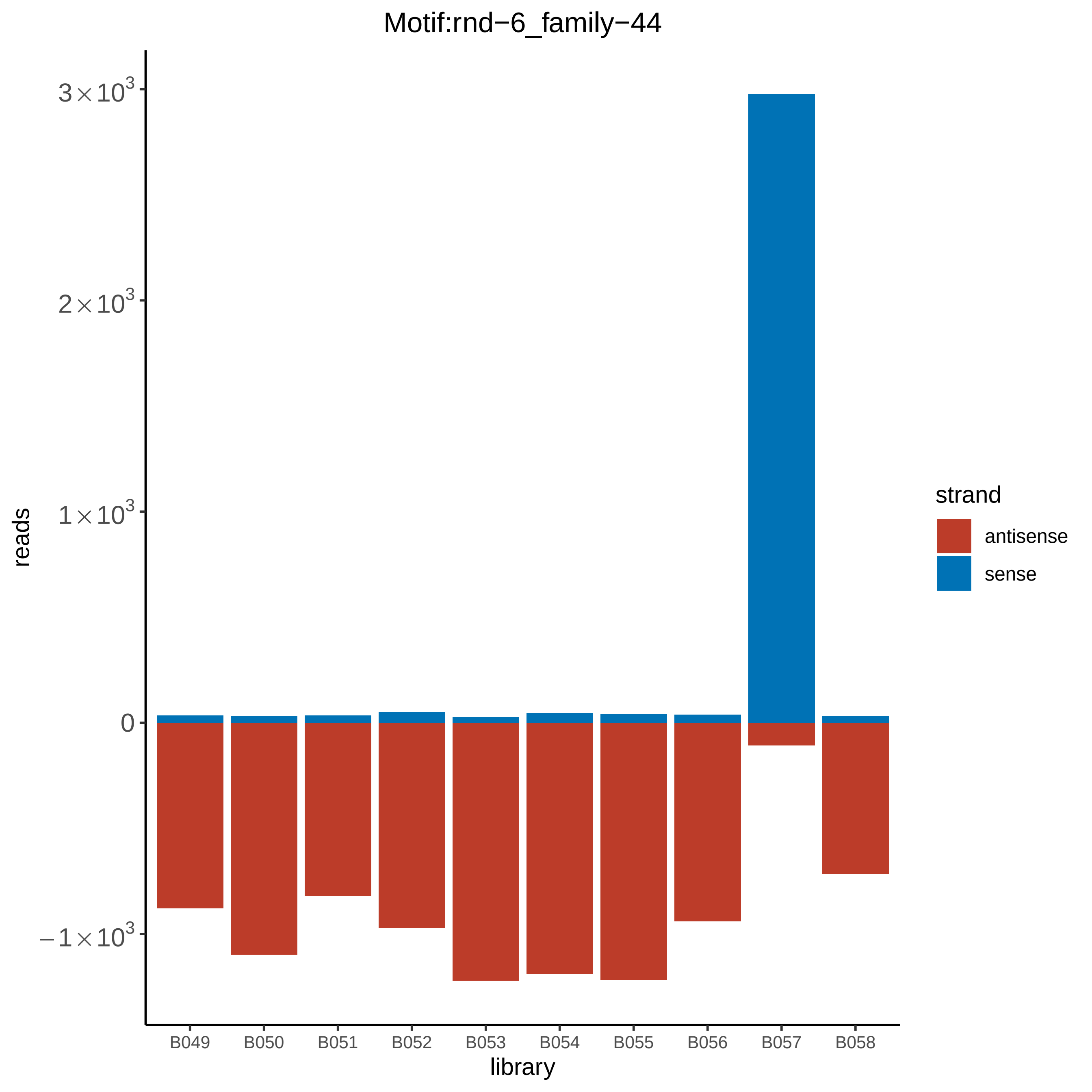

Motif:rnd−6_family−44
reads
3×10^3
2×10^3
1×10^3
0
−1×10^3
B049
B050
B051
B052
B053
B054
B055
B056
B057
B058
library
strand
antisense
sense

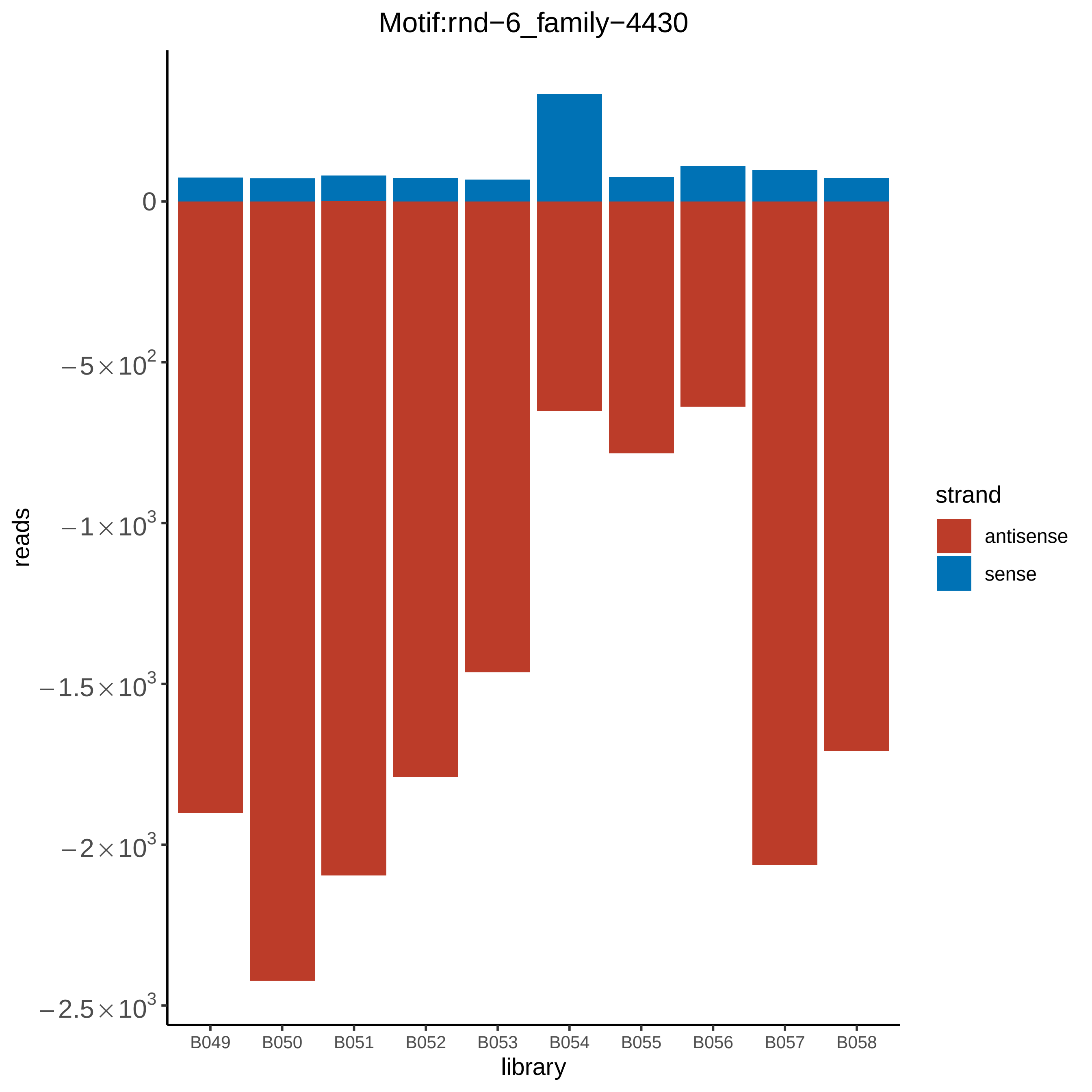
Motif:rnd−6_family−4430
reads
0
−5×10^2
−1×10^3
−1.5×10^3
−2×10^3
−2.5×10^3
B049
B050
B051
B052
B053
B054
B055
B056
B057
B058
library
strand
antisense
sense

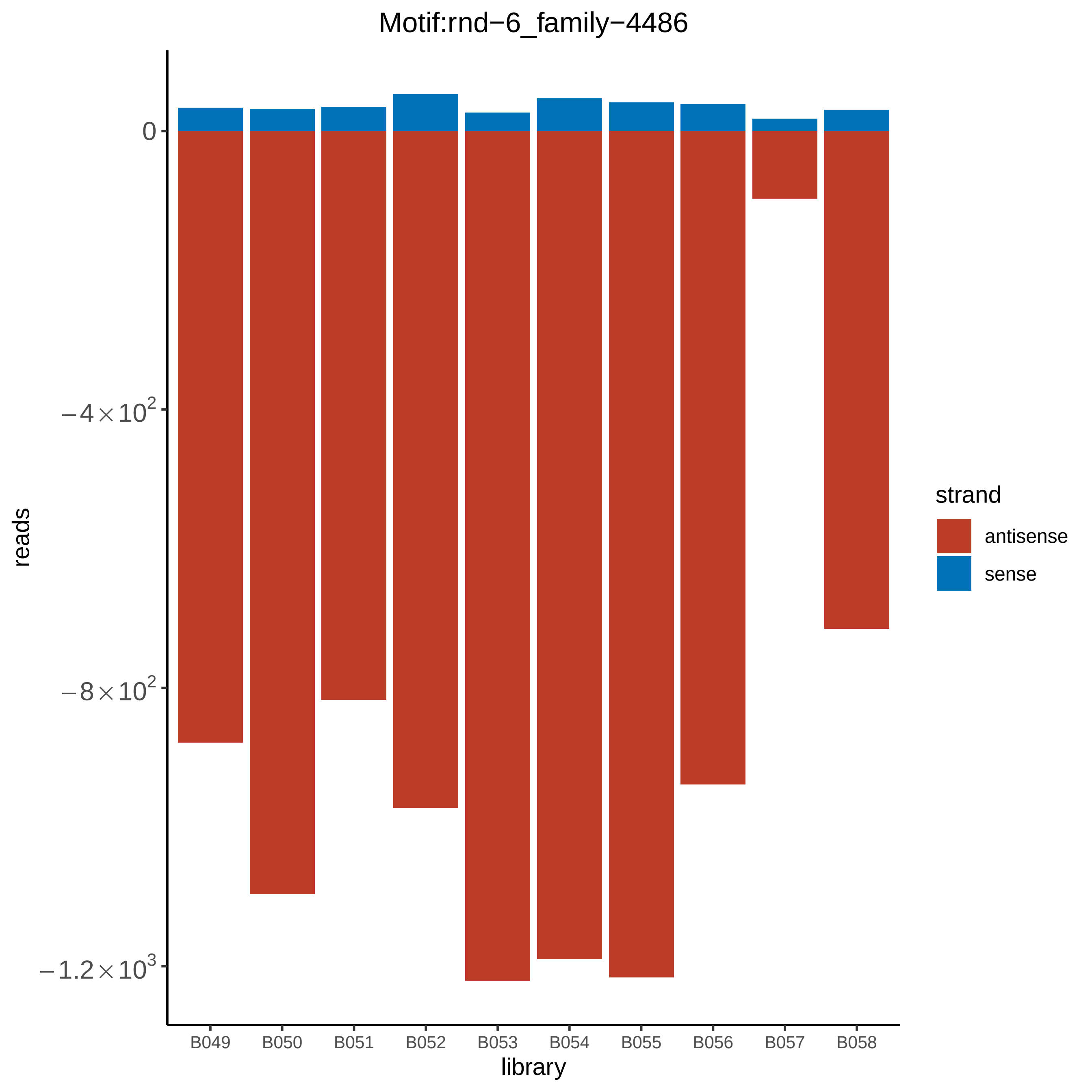

Motif:rnd−6_family−4486
reads
0
-4×10^2
-8×10^2
-1.2×10^3
B049
B050
B051
B052
B053
B054
B055
B056
B057
B058
library
strand
antisense
sense

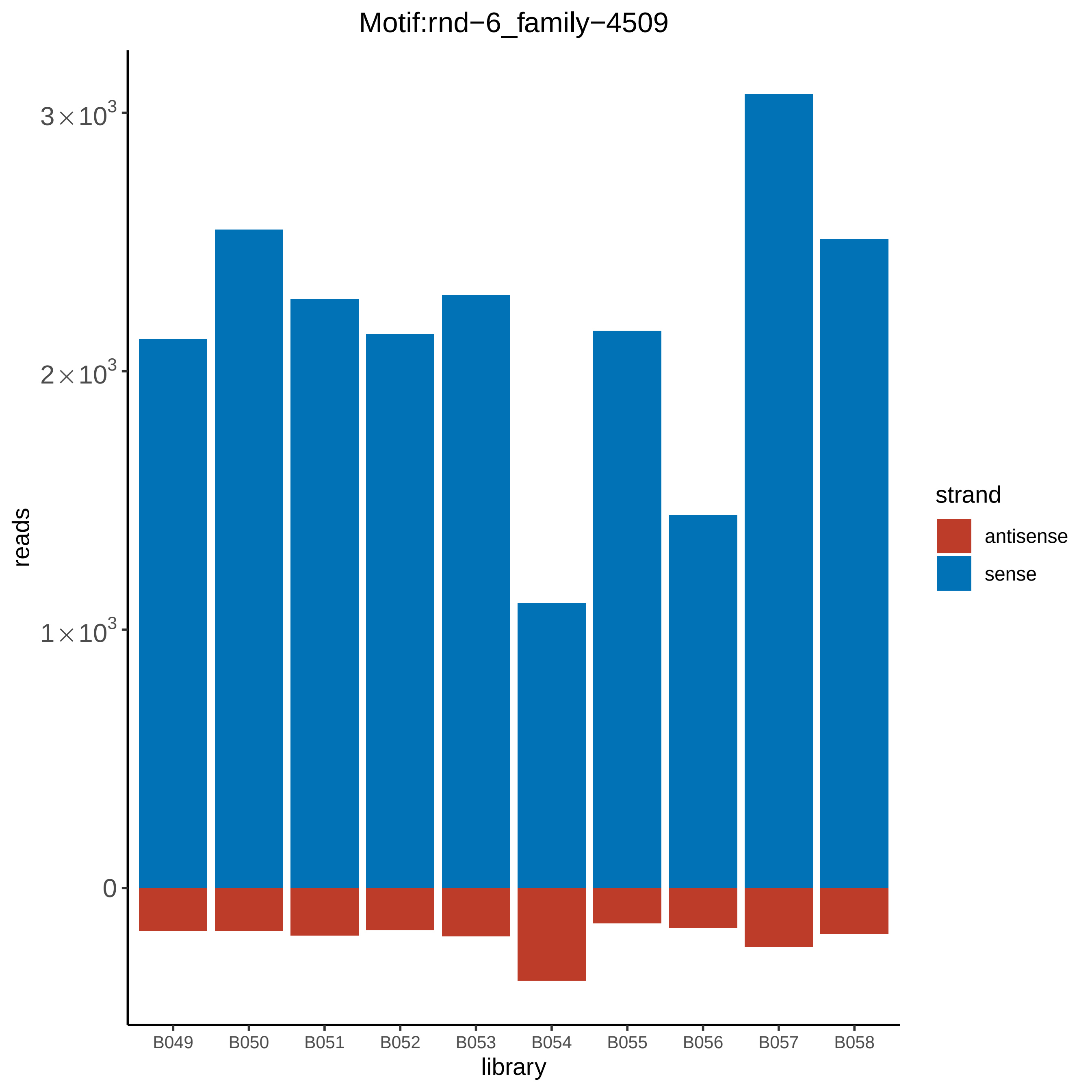

Motif:rnd−6_family−4509
reads
3×10^3
2×10^3
1×10^3
0
B049
B050
B051
B052
B053
B054
B055
B056
B057
B058
library
strand
antisense
sense

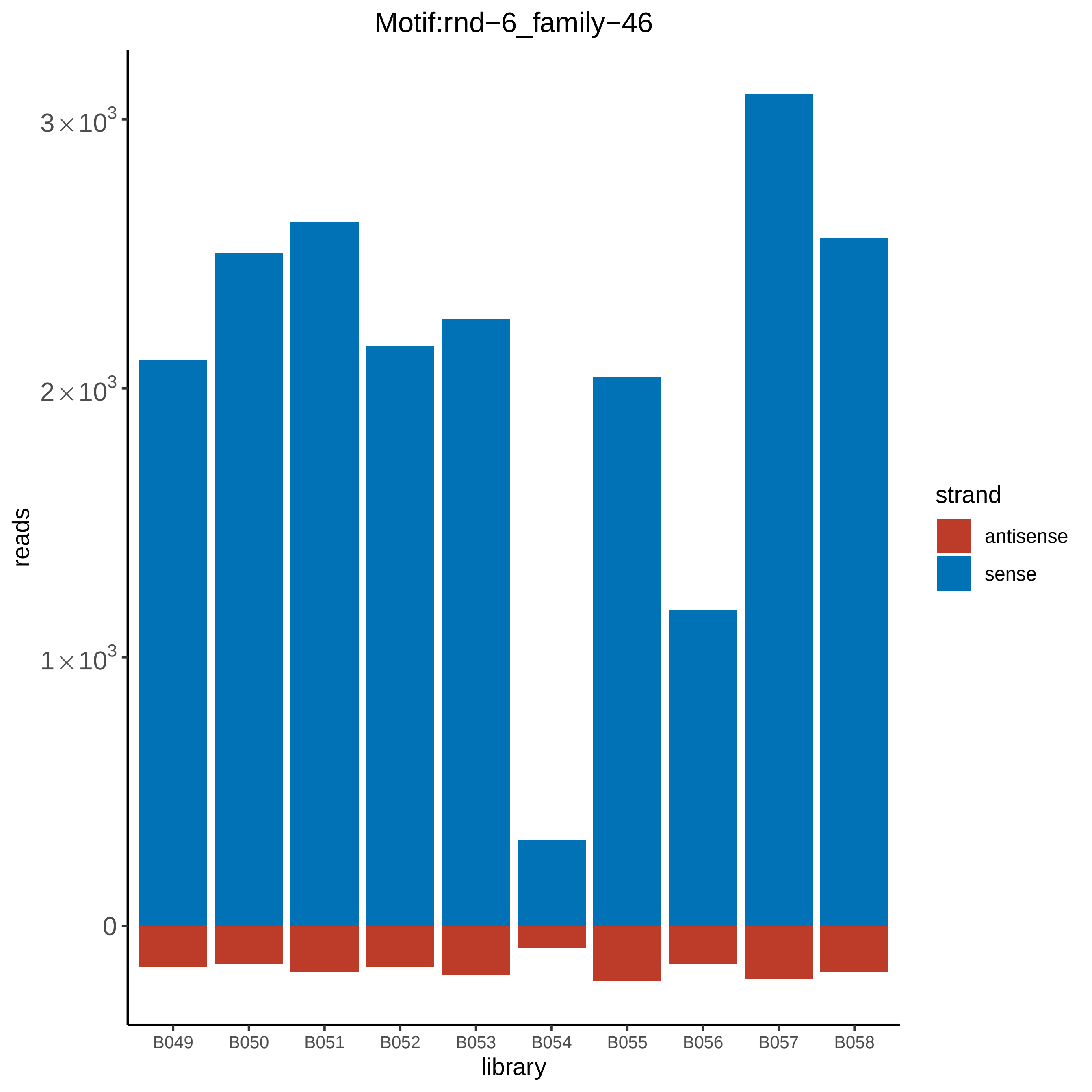

Motif:rnd−6_family−46
3×10³
2×10³
1×10³
0
reads
B049
B050
B051
B052
B053
B054
B055
B056
B057
B058
library
strand
antisense
sense

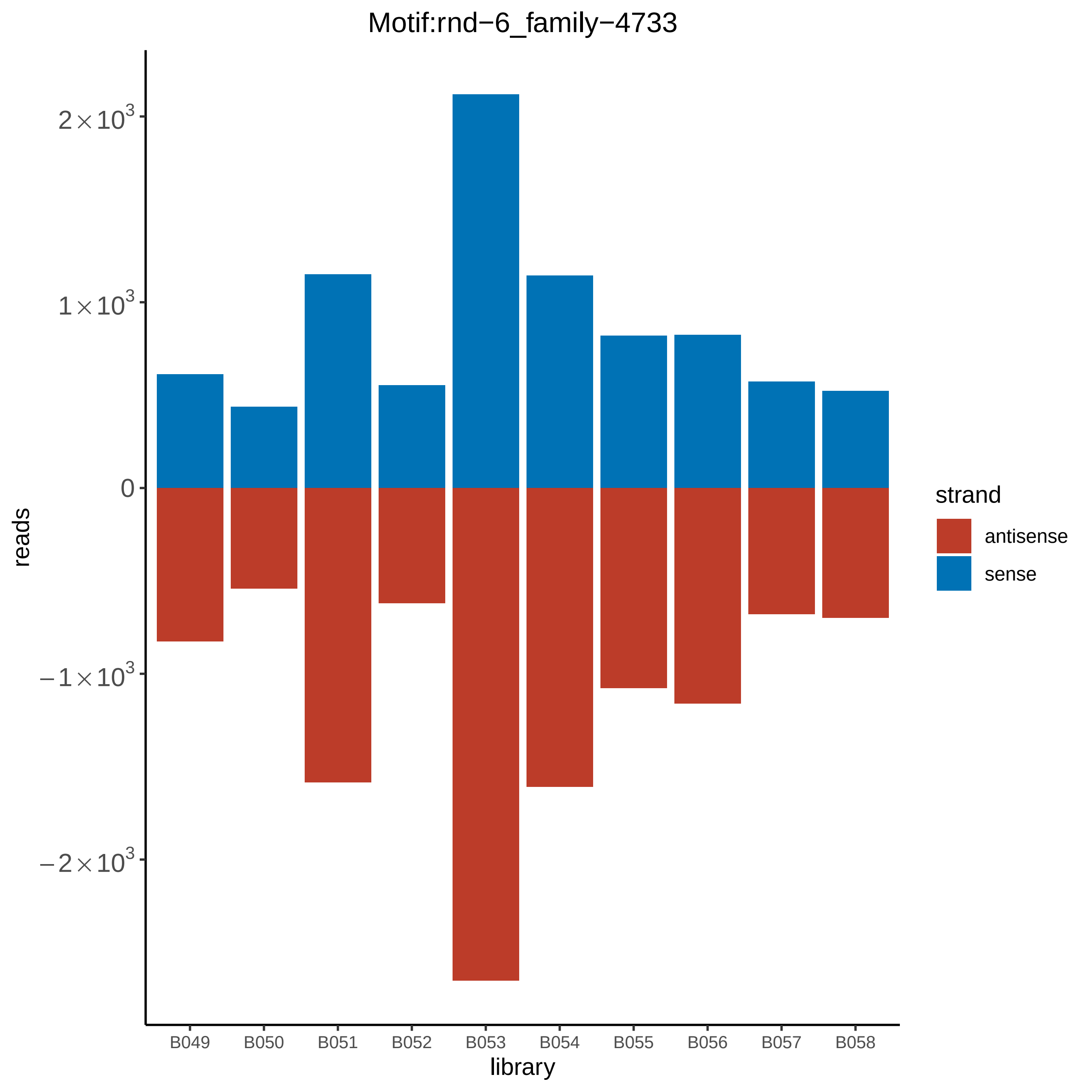
Motif:rnd−6_family−4733
2×10^3
1×10^3
0
-1×10^3
-2×10^3
reads
B049
B050
B051
B052
B053
B054
B055
B056
B057
B058
library
strand
antisense
sense

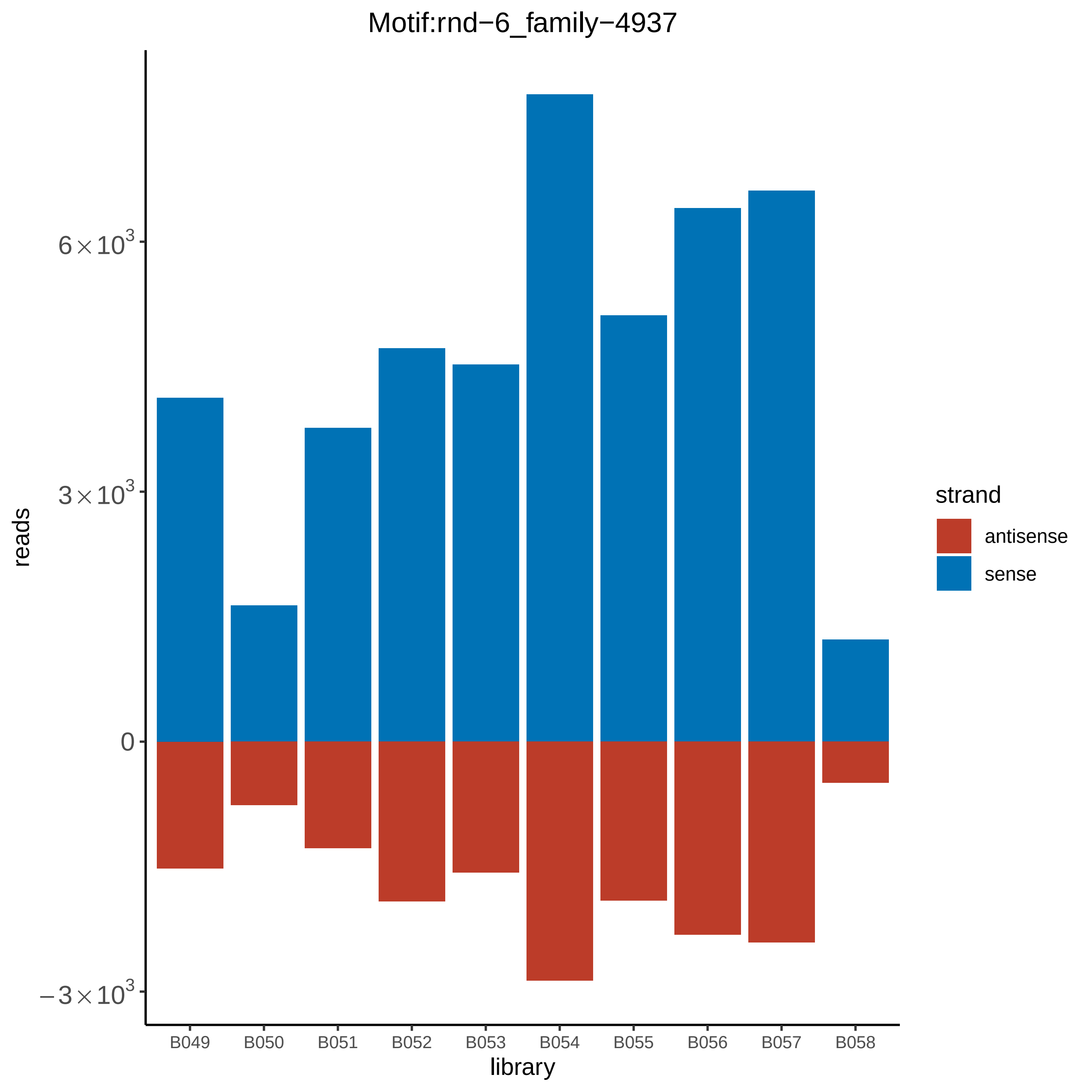

Motif:rnd−6_family−4937
reads
6×10^3
3×10^3
0
−3×10^3
library
B049
B050
B051
B052
B053
B054
B055
B056
B057
B058
strand
antisense
sense

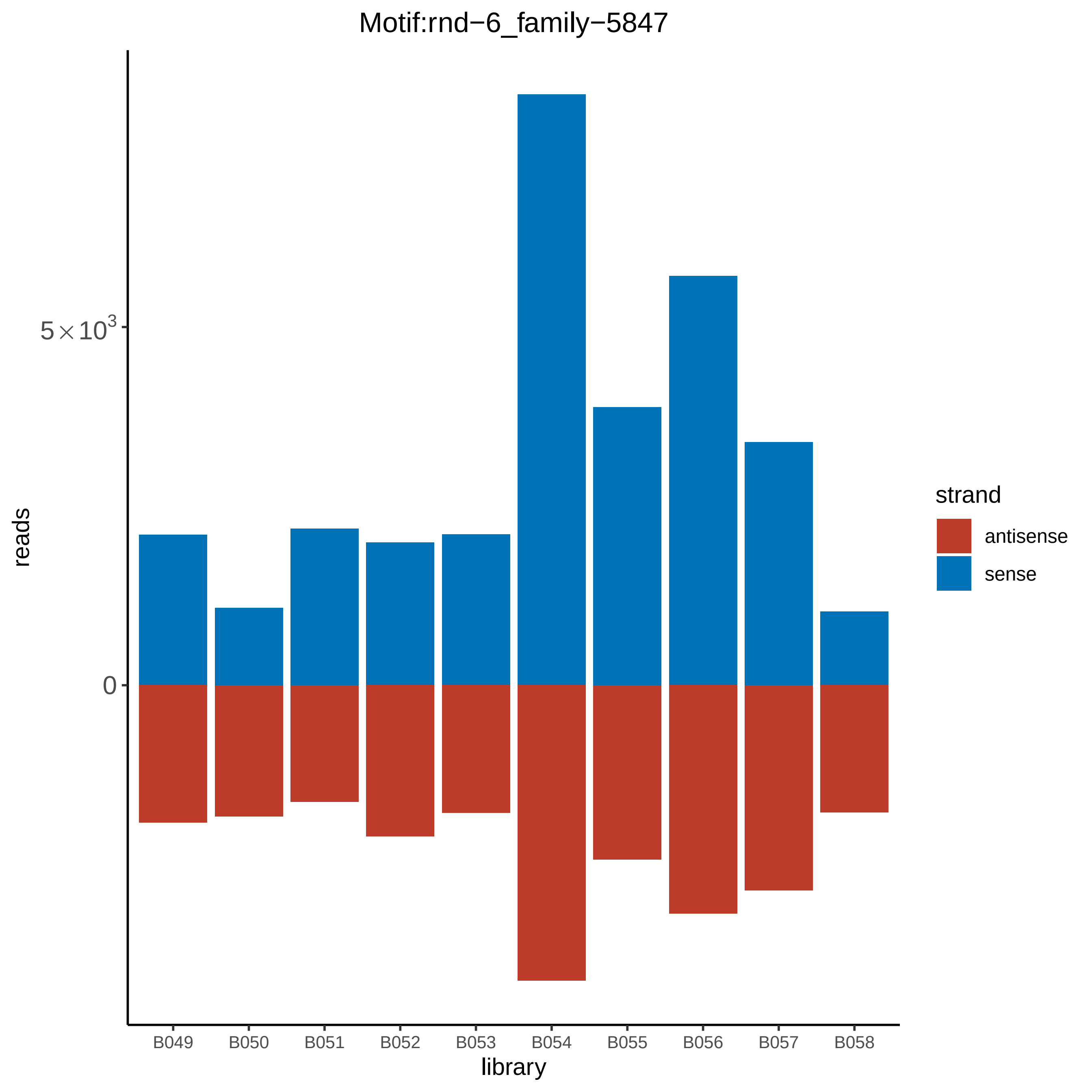

Motif:rnd−6_family−5847
5×10^3
0
reads
B049
B050
B051
B052
B053
B054
B055
B056
B057
B058
library
strand
antisense
sense

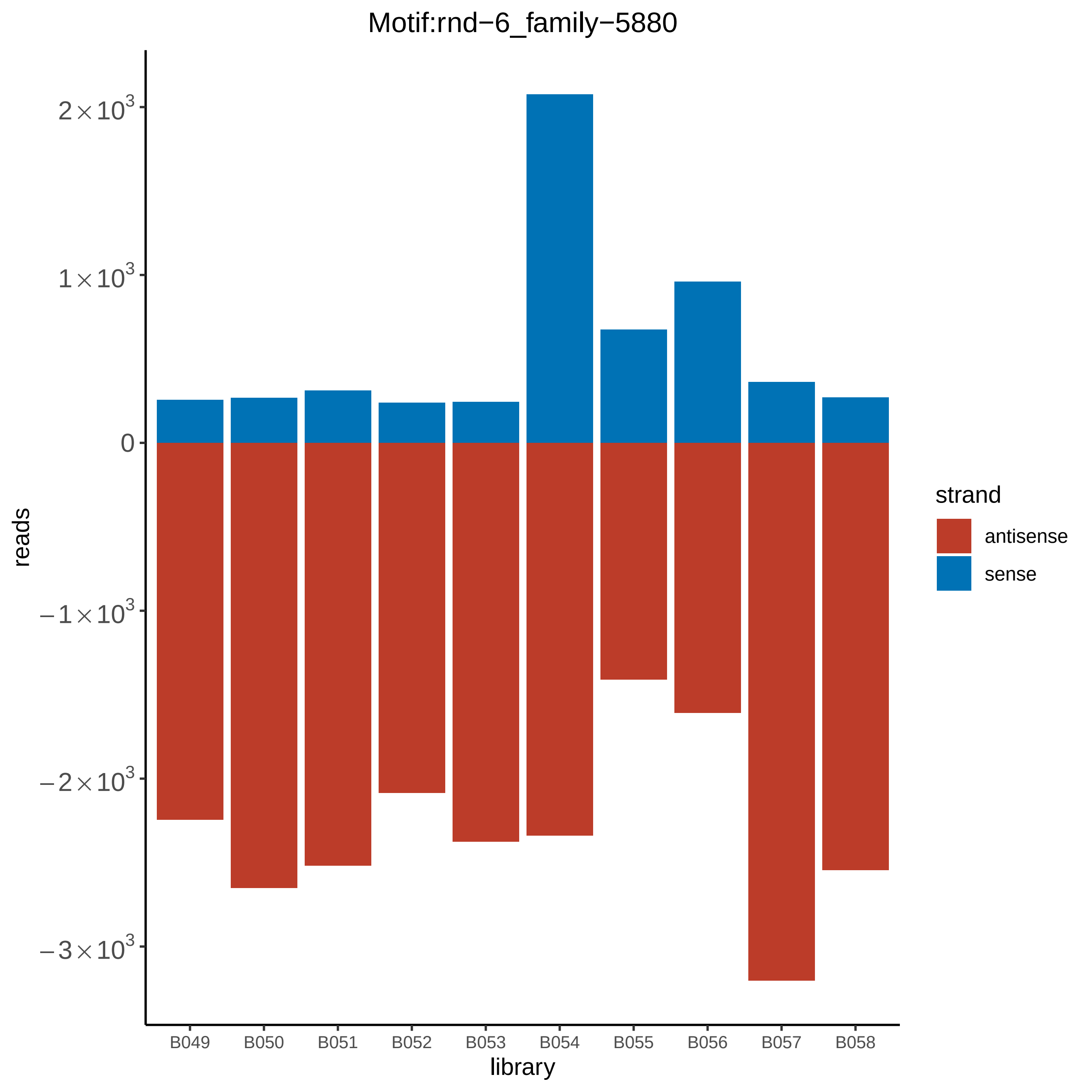

Motif:rnd−6_family−5880
2×10^3
1×10^3
0
−1×10^3
−2×10^3
−3×10^3
reads
B049
B050
B051
B052
B053
B054
B055
B056
B057
B058
library
strand
antisense
sense

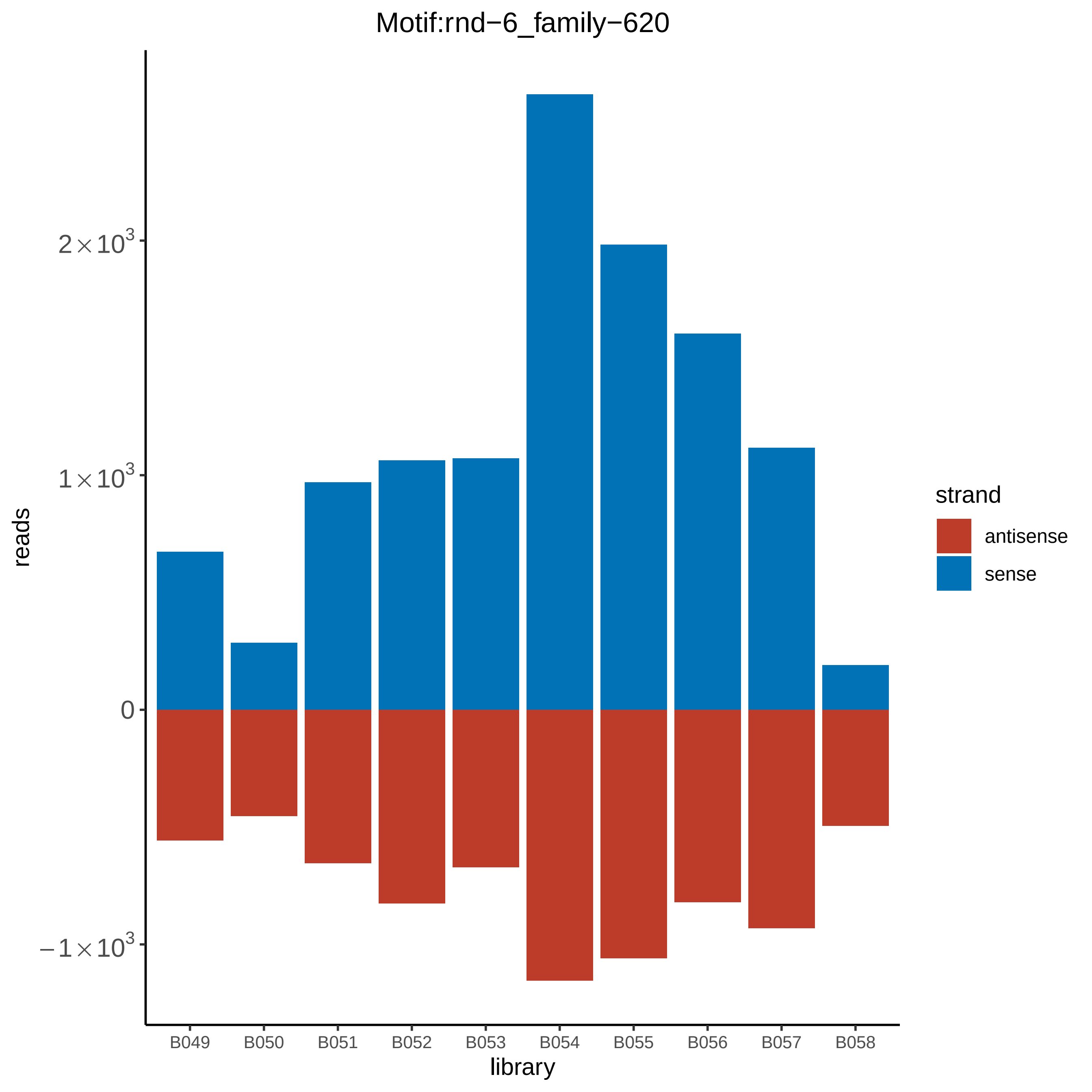

Motif:rnd−6_family−620
reads
2×10^3
1×10^3
0
−1×10^3
library
B049
B050
B051
B052
B053
B054
B055
B056
B057
B058
strand
antisense
sense

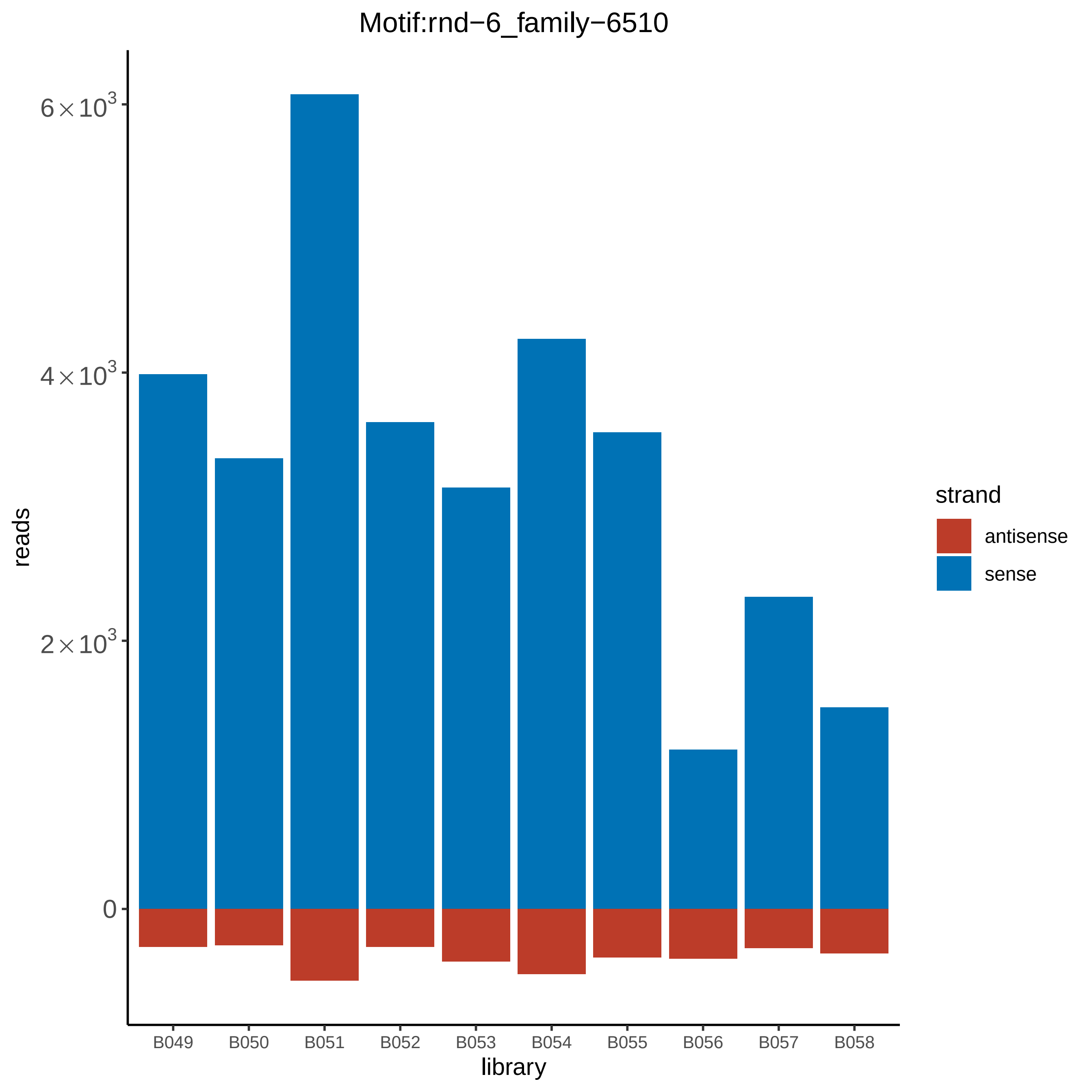

Motif:rnd−6_family−6510
reads
6×10^3
4×10^3
2×10^3
0
library
B049
B050
B051
B052
B053
B054
B055
B056
B057
B058
strand
antisense
sense

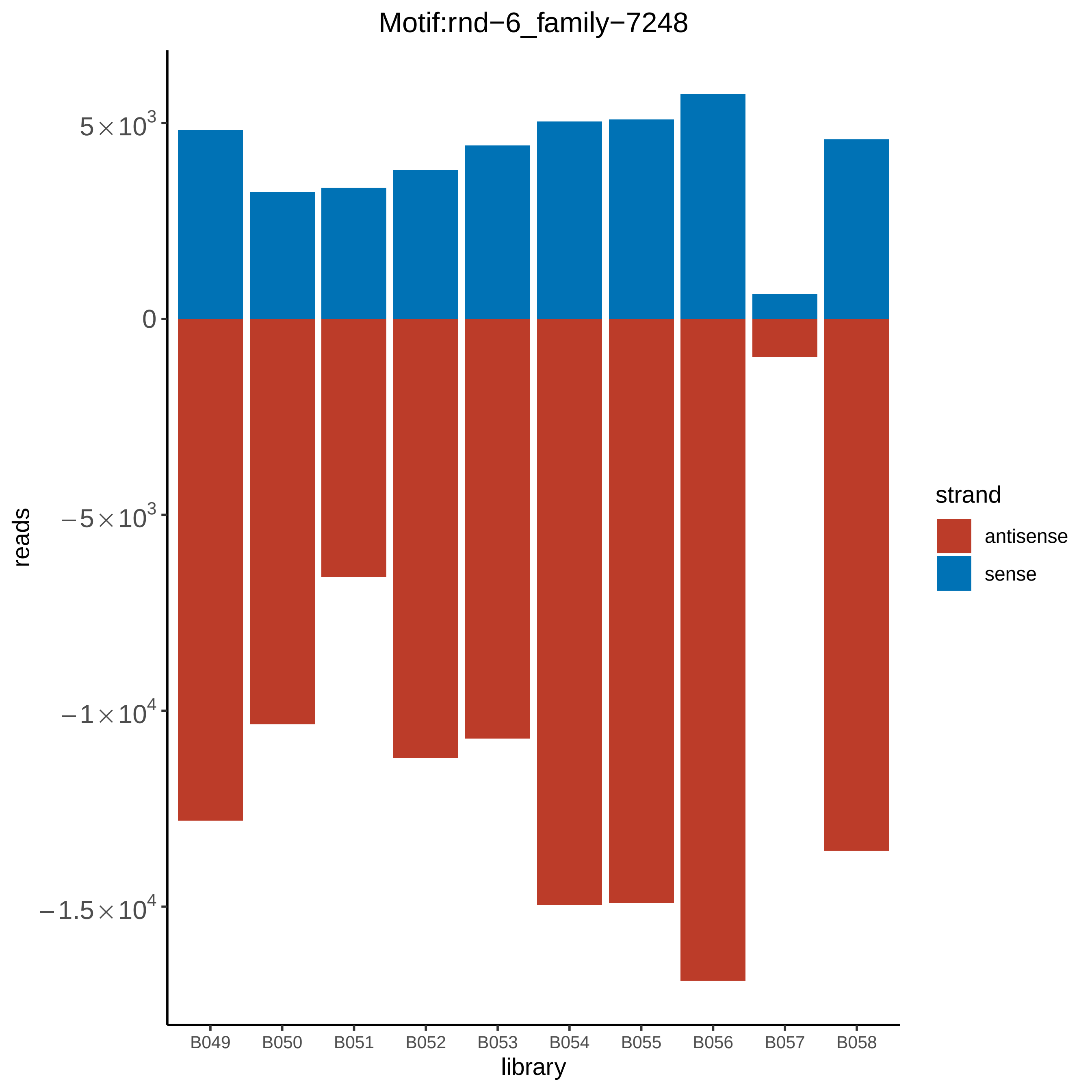

Motif:rnd−6_family−7248
5×10^3
0
−5×10^3
−1×10^4
−1.5×10^4
reads
B049
B050
B051
B052
B053
B054
B055
B056
B057
B058
library
strand
antisense
sense

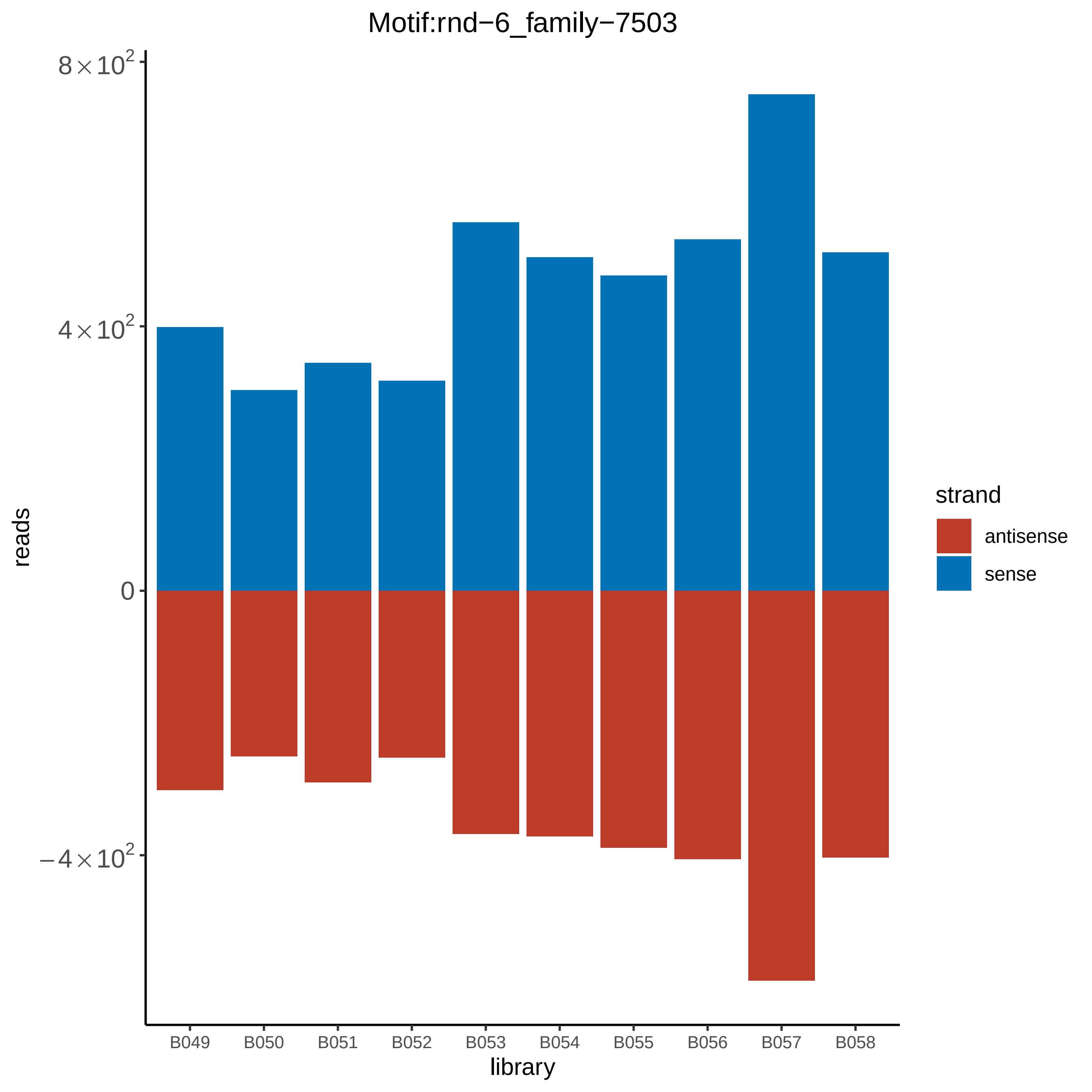

Motif:rnd−6_family−7503
8×10^2
4×10^2
0
−4×10^2
reads
B049
B050
B051
B052
B053
B054
B055
B056
B057
B058
library
strand
antisense
sense

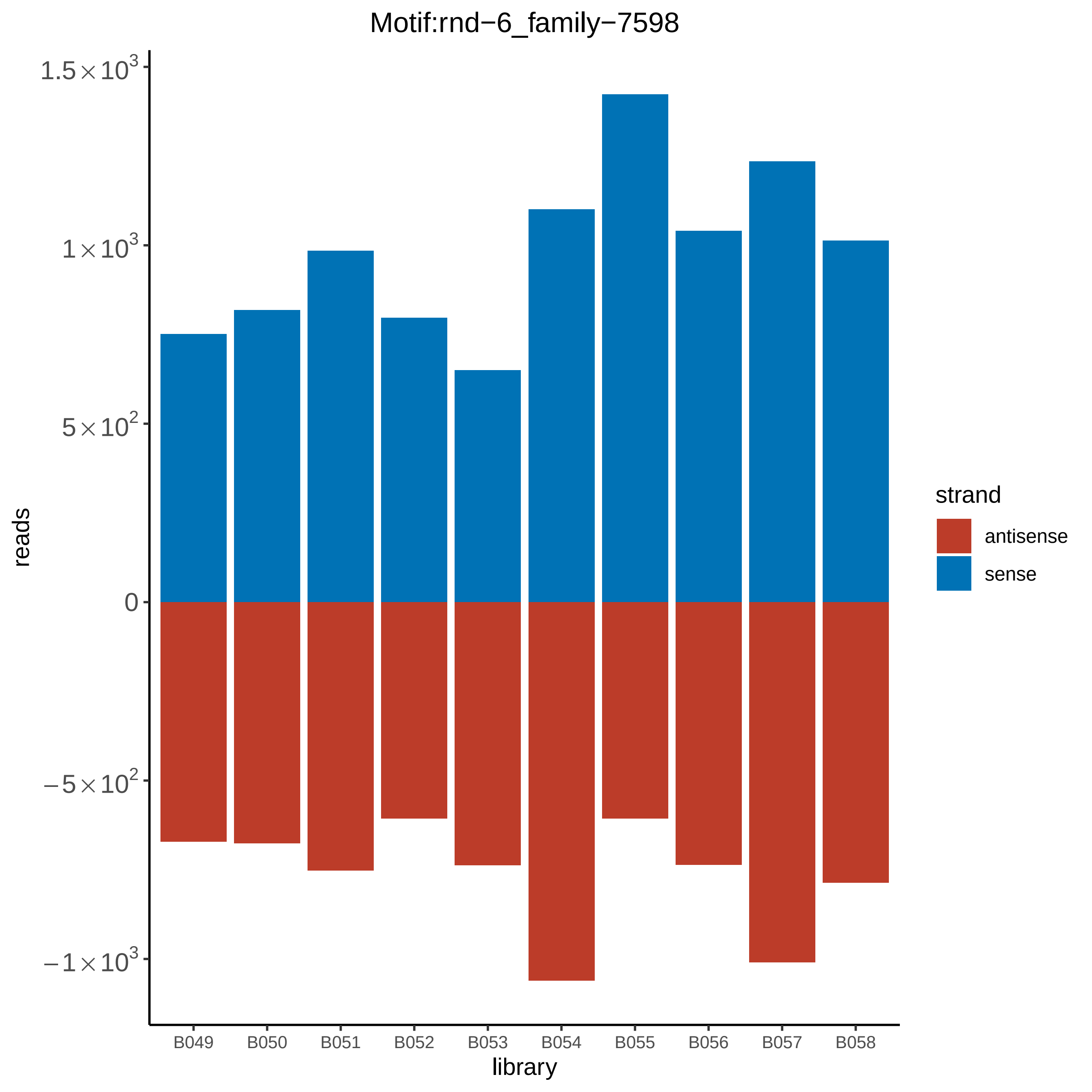

Motif:rnd−6_family−7598
reads
1.5×10^3
1×10^3
5×10^2
0
-5×10^2
-1×10^3
B049
B050
B051
B052
B053
B054
B055
B056
B057
B058
library
strand
antisense
sense

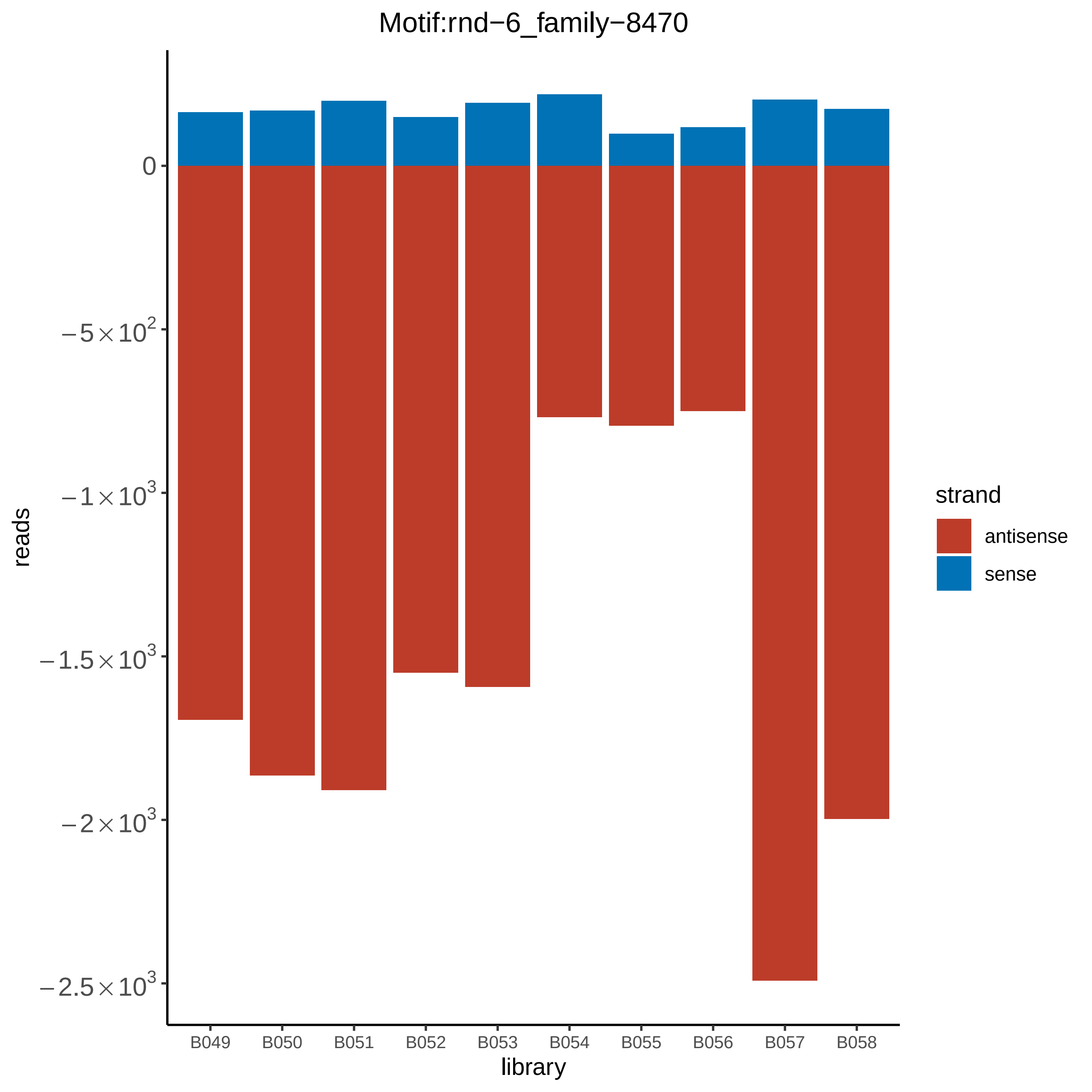

Motif:rnd−6_family−8470
0
−5×10^2
−1×10^3
−1.5×10^3
−2×10^3
−2.5×10^3
reads
B049
B050
B051
B052
B053
B054
B055
B056
B057
B058
library
strand
antisense
sense

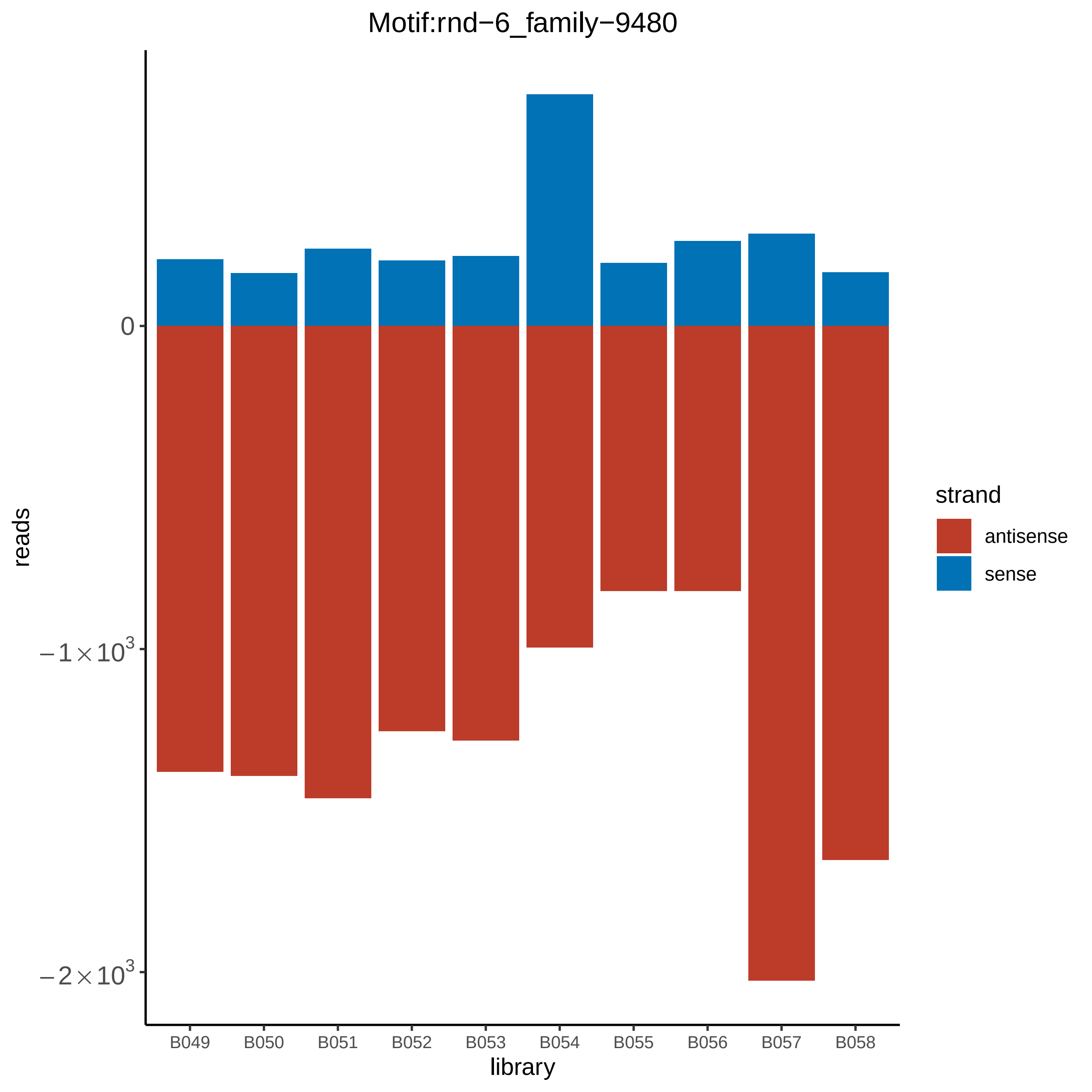

Motif:rnd−6_family−9480
reads
0
−1×10^3
−2×10^3
B049
B050
B051
B052
B053
B054
B055
B056
B057
B058
library
strand
antisense
sense

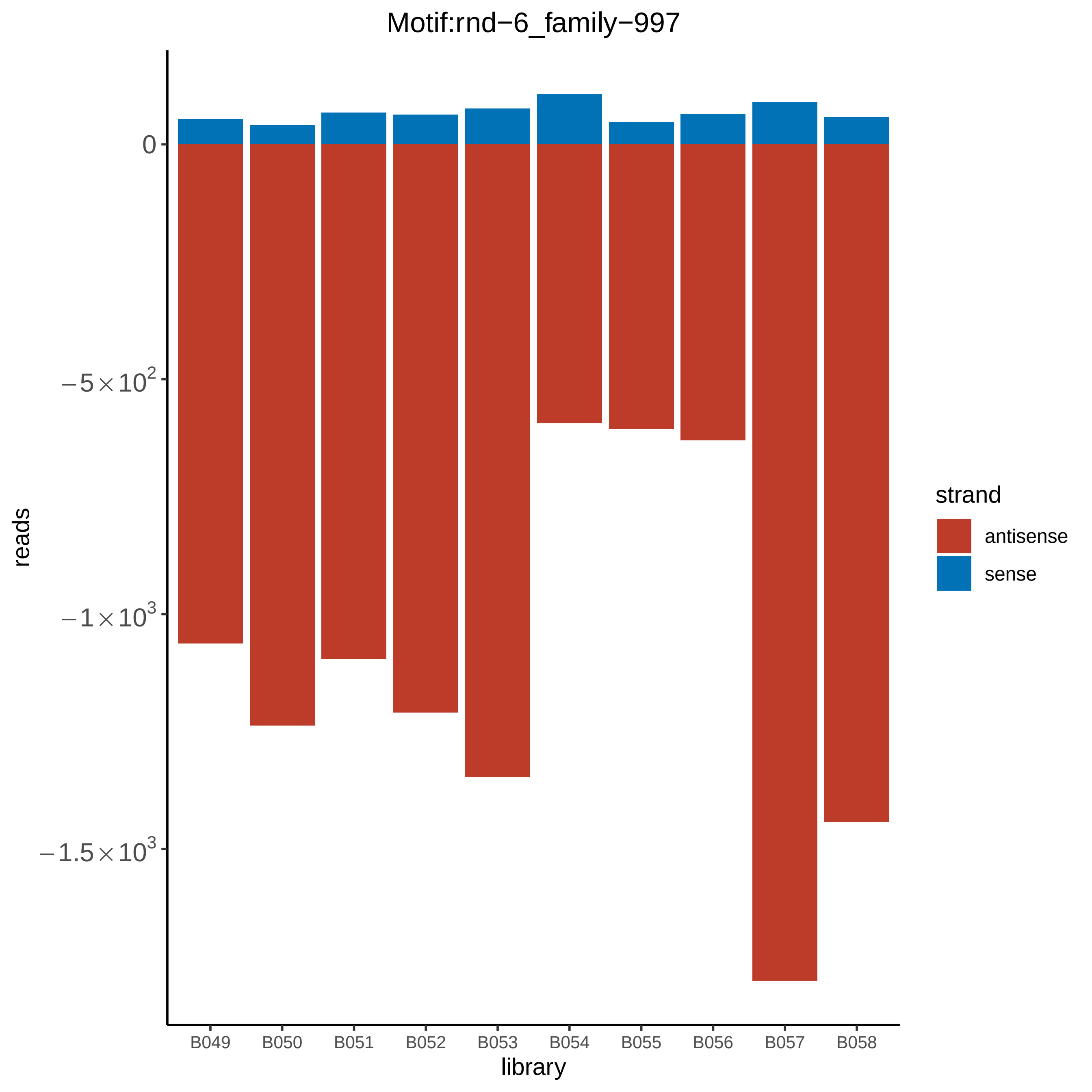

Motif:rnd−6_family−997
0
−5×10^2
−1×10^3
−1.5×10^3
reads
B049
B050
B051
B052
B053
B054
B055
B056
B057
B058
library
strand
antisense
sense
